# Functional Activity of Enantiomeric Oximes and Diastereomeric Amines and Cyano Substituents at C9 in 3-Hydroxy-*N*-phenethyl-5-phenylmorphans

**DOI:** 10.3390/molecules29091926

**Published:** 2024-04-23

**Authors:** Hudson G. Roth, Madhurima Das, Agnieszka Sulima, Dan Luo, Sophia Kaska, Thomas E. Prisinzano, Andrew T. Kerr, Arthur E. Jacobson, Kenner C. Rice

**Affiliations:** 1 Drug Design and Synthesis Section, Molecular Targets and Medications Discovery Branch, Intramural Research Program, National Institute on Drug Abuse and the National Institute on Alcohol Abuse and Alcoholism, National Institutes of Health, Department of Health and Human Services, 9800 Medical Center Drive, Bethesda, MD 20892-3373, USA; hudsogroth@gmail.com (H.G.R.); madhurima.das.91@gmail.com (M.D.); agnieszka.sulima@nih.gov (A.S.); 2 Department of Pharmaceutical Sciences, College of Pharmacy, University of Kentucky, 789 S. Limestone Street, Lexington, KY 40536, USA; dan.luo@uky.edu (D.L.); sophia.kaska.phd@gmail.com (S.K.); prisinzano@uky.edu (T.E.P.); 3 Center for Biomolecular Science and Engineering, Naval Research Laboratory, Washington, DC 20375-0001, USA; andrew.kerr@nrl.navy.mil

**Keywords:** enantiomeric, diastereomeric, stereochemical, chiral C9 amino, methylamino, cyanomethyl, methylaminomethyl-substituted 5-phenylmorphans, inhibition of forskolin-induced cAMP accumulation assay (cAMP assay), 3-(2-phenethyl-2-azabicyclo[3.3.1]nonan-5-yl)phenol

## Abstract

The synthesis of stereochemically pure oximes, amines, saturated and unsaturated cyanomethyl compounds, and methylaminomethyl compounds at the C9 position in 3-hydroxy-*N*-phenethyl-5-phenylmorphans provided μ-opioid receptor (MOR) agonists with varied efficacy and potency. One of the most interesting compounds, (2-((1*S*,5*R*,9*R*)-5-(3-hydroxyphenyl)-2-phenethyl-2-azabicyclo[3.3.1]nonan-9-yl)acetonitrile), was found to be a potent partial MOR agonist (EC_50_ = 2.5 nM, %E_max_ = 89.6%), as determined in the forskolin-induced cAMP accumulation assay. Others ranged in potency and efficacy at the MOR, from nanomolar potency with a C9 cyanomethyl compound (EC_50_ = 0.85 nM) to its totally inactive diastereomer, and three compounds exhibited weak MOR antagonist activity (the primary amine **3**, the secondary amine **8**, and the cyanomethyl compound **41**). Many of the compounds were fully efficacious; their efficacy and potency were affected by both the stereochemistry of the molecule and the specific C9 substituent. Most of the MOR agonists were selective in their receptor interactions, and only a few had δ-opioid receptor (DOR) or κ-opioid receptor (KOR) agonist activity. Only one compound, a C9-methylaminomethyl-substituted phenylmorphan, was moderately potent and fully efficacious as a KOR agonist (KOR EC_50_ = 18 nM (% E_max_ = 103%)).

## 1. Introduction

**Figure 1:**
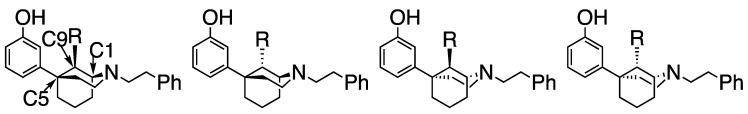
Four structural isomers for the C9 amino, cyanomethyl, and methylaminomethyl substituents (R).

Over the past several decades, opioid abuse in the United States has steadily increased, resulting in a large number of dependent individuals and opioid-related deaths [1,2]. These circumstances ultimately led to the declaration of an opioid crisis as a “public health emergency” by the United States Federal Government in 2017. We are pursuing several approaches to help combat this public health problem, one of which focuses on developing safer opioid alternatives that could also be used as medications for Opioid Use Disorder (OUD). To achieve this goal, we are synthesizing low-efficacy compounds based on the 5-phenylmorphan structure. Depending on the stereochemistry of the molecule and the specific substituent at C9 in the *N*-phenethyl-5-phenylmorphans (Figure 1), we found full or partial agonists with varied potency [3,4,5,6,7] at the μ-opioid receptor (MOR). Different ligands at the C9 position of the 5-phenylmorphan or compounds with a specific stereochemistry can interact with an active conformation of the receptor as a MOR agonist and turn on involved intracellular pathways [8] or act at the inactive conformation of the MOR as an opioid antagonist [5]. One signaling molecule recruited by the MOR in its active conformation is β-arrestin2, which has been proposed to play a role in manifesting the negative side effects associated with opioids [9]. However, the importance of β-arrestin2 has been challenged, and an attempt to reproduce studies that indicate biased ligands do not induce respiratory depression was unsuccessful [10]. Gillis et al. have proposed a different theory based on intrinsic efficacy. This concept indicates that agonists with higher intrinsic efficacy will produce increased downstream signaling and show well-known opioid-like side effects. As a result, an ideal opioid would need to provide a balance between inducing sufficient signaling, which may be correlated to its efficacy in the cAMP assay, to enable significant pain reduction in vivo but not so much as to cause negative side effects [11]. We attempt to measure that balance using in vitro assays (the forskolin-induced cAMP accumulation assay and the [^35^S]GTPgS assay) [5] and in vivo assays [12,13]. However, advocates for biased agonism have argued that the understanding of interactions at the MOR is still incomplete, and disregarding β-arrestin2 completely may not be correct.

The 5-phenylmorphan structure is the template for our new compounds. It contains a minimal structure of morphine that can induce antinociceptive activity [14]. The 5-phenylmorphans are sufficiently different from morphine and the opioid-like family of analgesics (e.g., oxymorphone, oxycodone, hydromorphone) that some of these 5-phenylmorphans, those with certain C9 substituents bearing specific stereochemistry, have been found to have a different pharmacological profile, enabling antinociception with less respiratory depression and minimal gastrointestinal effects. The side effects appear to be related to the efficacy of the compounds; those with minimal efficacy do not induce respiratory depression or show GI effects, but they retain some antinociceptive activity, depending on the in vitro or in vivo assay. Previous work by our group has shown that some C9-substituted derivatives containing an *N*-phenethyl moiety exhibit little or no β-arrestin2 recruitment [4]; others act as potent partial MOR agonists. Because of the promise this family of molecules holds as a new type of analgesic, we have designed 5-(3-hydroxphenyl)-*N*-phenethyl-phenylmorphan derivatives containing varied substituents at the C9 position and have found suitable synthetic paths to obtain sufficient material for pharmacological evaluation (Figure 1). The C9 position was chosen for our initial exploration because our early work indicated that potent MOR agonists could be obtained with chiral substituents at that position [3], and there appeared to be a minimal amount of previous synthetic work based on that area of the molecule.

In this work, we discuss the synthesis and in vitro functional activity of chiral 3-hydroxy-*N*-phenethyl-5-phenylmorphans with various nitrogen-containing substituents at C9. These substituents were oximes, amines, unsaturated and saturated cyanomethyl compounds, and methylaminomethyl substituted compounds (Figure 1).

## 2. Results and Discussion

### 2.1. Synthesis of Chiral C9-Substituted 5-Phenylmorphans

**Scheme 1:**
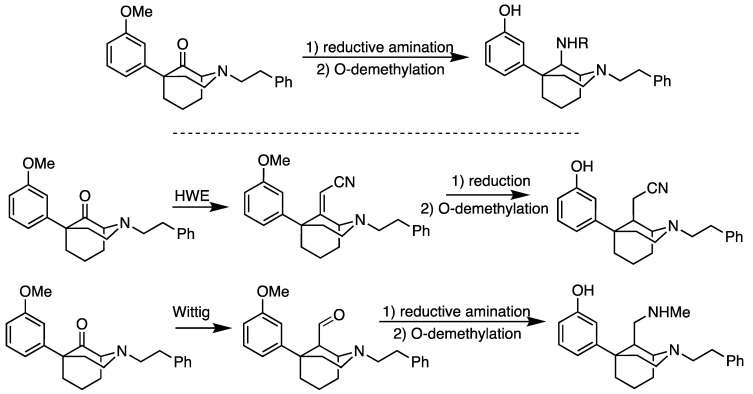
General derivatization strategy.

We have developed a library of *N*-phenethyl-5-(3-hydroxyphenyl)morphan stereoisomers with a variety of substituents at the C9 position [4,5,6,7]. As additions to this library, we have now synthesized amines separated from the C9 position by zero or one carbon atom, as well as two enantiomeric oximes at C9 and unsaturated and saturated cyano derivatives at C9. The general approach to the amine target compounds involved either a reductive amination or a Horner–Wadsworth–Emmons (HWE) reaction on 5-(3-methoxyphenyl)-9-oxo-2-phenylethyl-2-azabicyclo93.3.1)nonane (9-keto phenylmorphan) (Scheme 1) with known stereochemistry.

### 2.2. Synthesis of 1S,5S,9R- and 1R,5R,9S-C9 Amino and Methylamino Enantiomers

**Scheme 2:**
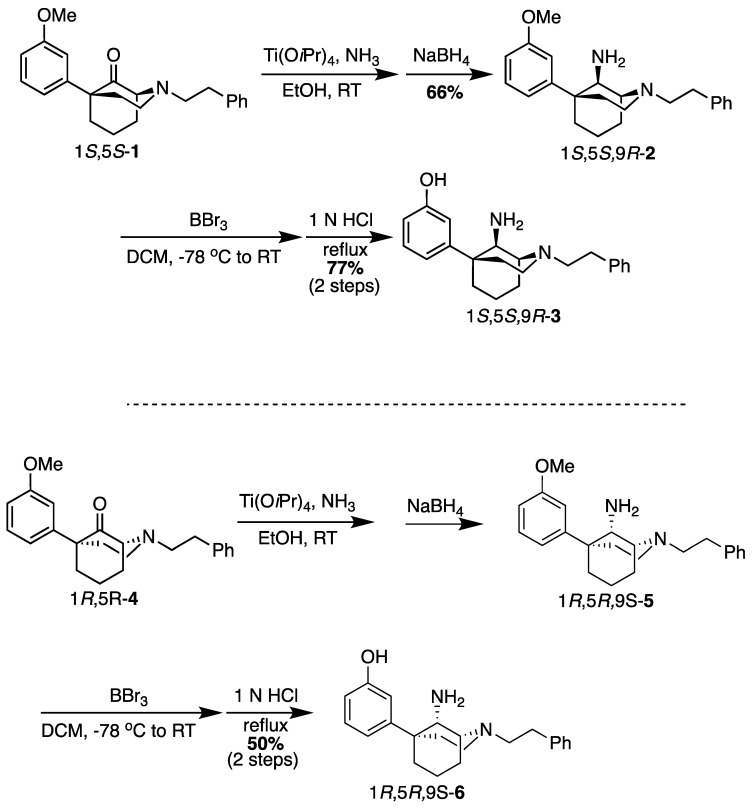
Titanium-mediated reductive amination to obtain enantiomeric C9 amino compounds **3** and **6**.

The enantiomeric (1*S*,5*S*- and 1*R*,5*R*-) 9-keto-*m*-methoxyphenylmorphans were obtained using the literature procedure [7,15]. Reductive amination was found to allow the synthesis of C9 amino derivatives with zero carbons at C9, while the HWE reaction facilitates the formation of unsaturated and saturated cyano derivatives. A Wittig reaction to the aldehyde, followed by reductive amination and O-demethylation, gave N-methyl compounds with one carbon at C9. We began with an exploration of the use of a titanium-mediated reductive amination on the enantiomeric 9-keto compounds 1*S*,5*S*-**1** and 1*R*,5*R*-**4** (Scheme 2).

**Scheme 3:**
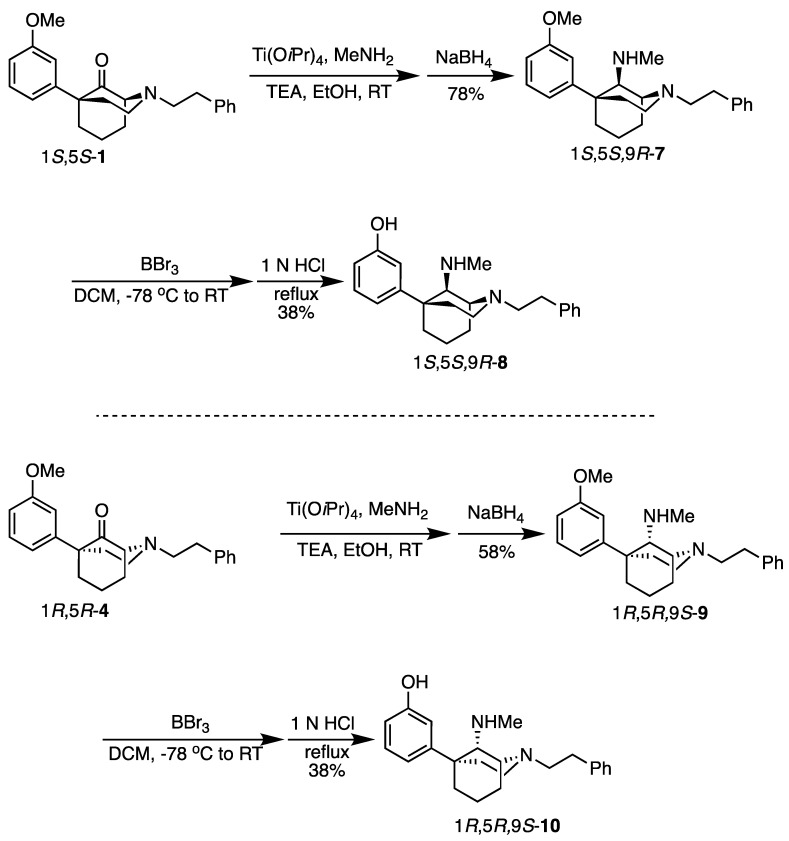
Titanium-mediated reductive amination to obtain enantiomeric C9 aminomethyl compounds **8** and **10**.

This approach was successful with both ammonia (Scheme 2) and methyl amine (Scheme 3) as the nucleophiles. This titanium-mediated reaction gave a product with the C9 amino group *cis* to the tertiary amine.

While the reaction with ammonia proceeded at room temperature, methylamine required heat. In addition, we discovered that a pressure tube is needed for the latter transformation, as the methylamine is lost to the atmosphere faster than it can react with **1** if the reaction is not performed in a sealed container.

Despite the success of these reactions, they only gave one of the diastereomers for each of the 9-keto enantiomers. This was likely due to the titanium (IV) coordinating with the tertiary nitrogen in **1**, effectively blocking one side of the molecule from the hydride approach during the reduction step.

**Scheme 4:**
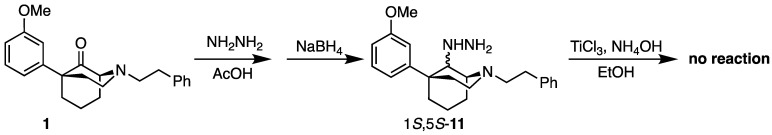
Attempted hydrazine reduction.

Based on the success of the transformation in Scheme 2 and Scheme 3, we considered accessing the two remaining diastereomers of the C9 amino compound via the reduction of the corresponding hydrazine (Scheme 4).

We found that reacting **1** with hydrazine followed by a reduction with NaBH_4_ gave a single isomer of **11**, as suggested by ^1^H NMR (Scheme 4). However, attempts at cleaving the nitrogen–nitrogen bond under basic conditions were unsuccessful.

**Scheme 5:**
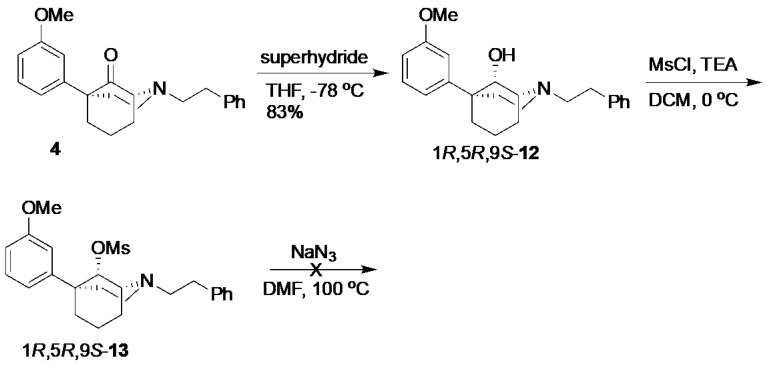
Attempted azide substitution.

We also considered the possibility of accessing other diastereomers via an azide. We began this process by performing a diastereoselective reduction of 1*R*,5*R*-**4** with superhydride to form the alcohol **12**, followed by mesylating the alcohol **12** to give **13** (Scheme 5).

Attempts at a substitution with sodium azide at 60 °C were unsuccessful (giving only unreacted starting material), while heating the transformation up to 100 °C was also unsuccessful, and the desired product was not obtained from this reaction. Next, we subjected the chiral alcohol 1*R*,5*R*,9*S*-**12** to the Mitsunobu reaction in the hope of obtaining the desired 1*R*,5*R*,9*R*-isomer via complete inversion of stereochemistry at the C9 center. This approach was not successful either.

To access the two remaining C9 amino diastereomers, several other reductive amination conditions were attempted. Unfortunately, various Brønsted acids, reductants, and ammonia sources gave no evidence for product formation. Possibly the steric hindrance around the C9 position in **1** required a strong Lewis acid catalyst to facilitate a nucleophilic attack. We next investigated the use of benzylamine as the nucleophile, hoping that this might force the opposite configuration at C9 due to its larger size. Unfortunately, that did not give us the desired chiral product.

### 2.3. Synthesis of Oxime Precursors to the Remaining Set of C9 Amino Enantiomers

**Scheme 6:**
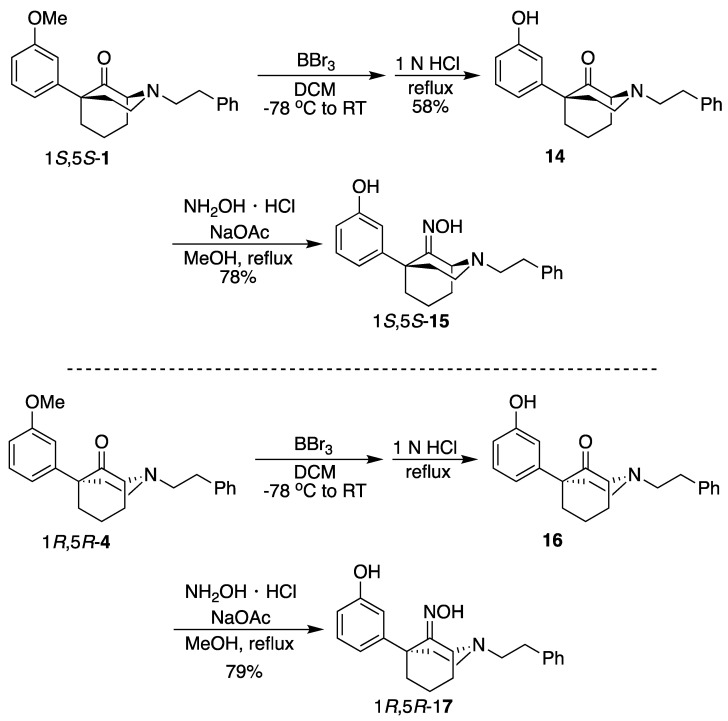
Synthesis of phenolic enantiomeric oximes *1S*,5*S*-**15**, and 1*R*,5*R*-**17** for functional assay.

We then considered accessing the two remaining C9 amino compounds through their oximes. This approach was successful, and we obtained the desired oximes **15** and **17** in sufficient quantity to enable an examination of their functional activity (Scheme 6).

**Scheme 7:**
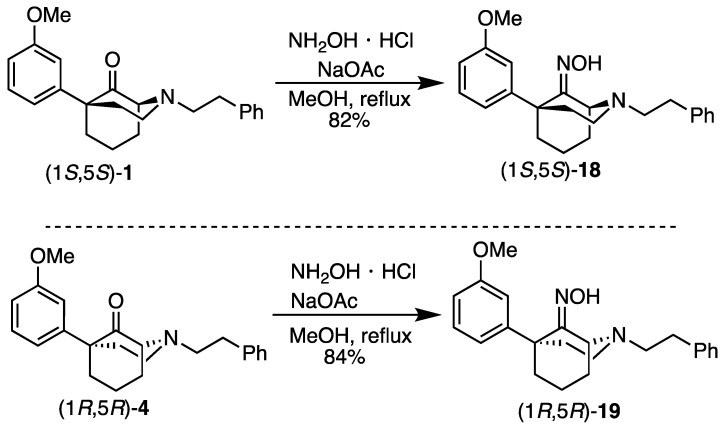
Synthesis of enantiomeric oximes **18** and **19**.

It was not necessary to convert the C9 keto methoxy compounds **1** and **4** to their phenolic relatives. Oximes could be prepared directly from the phenolic ether to obtain the methoxy-substituted oximes **18** and **19** (Scheme 7). The E/Z stereochemistry of the oximes **15**, **17**, **18**, and **19** was not established.

### 2.4. Synthesis of the 1S,5S,9S- and 1R,5R,9R-C9 Amino Enantiomers

The reduction of the methoxy ether oxime **19** was tested under three conditions: using Red-Al, hydrogenation via an H-cube flow reactor, and using LAH. While all three conditions were found to give a mixture of diastereomers, the yields and ratios between the products varied (Table 1).

**Scheme 8:**
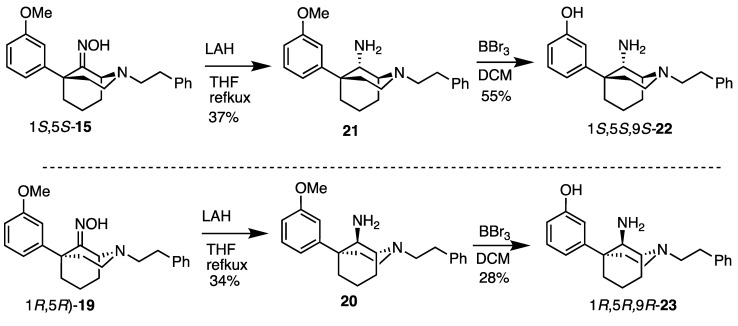
Synthesis of enantiomeric phenolic amines **22** and **23**—the diastereomers of **3** and **6**.

Despite the low yield that was observed in this initial LAH reduction, likely due to a loss of product from coordination with aluminum salt byproducts, we were able to access the desired phenolic amino compounds **22** and **23** after O-demethylation (Scheme 8).

### 2.5. Stereochemistry of the C9 Amino and Cyanomethyl Compounds

#### 2.5.1. Chirality Determined via X-ray Crystallography

**Figure 2:**
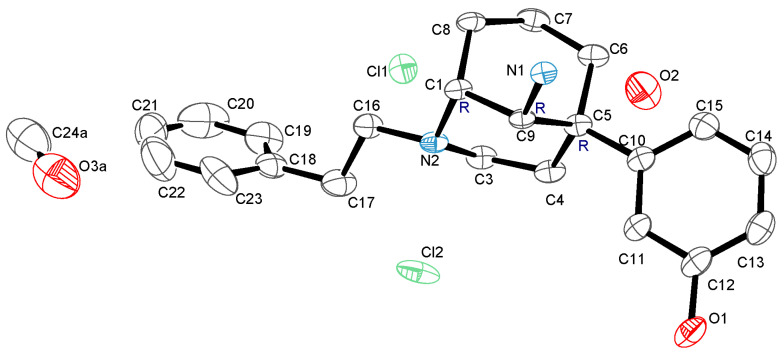
X-ray crystallographic structure of 3-((1*R*,5*R*,9*R*)-9-amino-2-phenethyl-2-azabicyclo[3.3.1]nonan-5-yl)phenol (**23**) dihydrochloride. The thermal ellipsoids are shown at the 50% probability level.

The stereochemistry of these C9-substituted phenylmorphans was confirmed by X-ray diffraction analysis (Figure 2).

#### 2.5.2. Chirality Determined via NMR Analysis

The ^1^H-NMR data were indicative of the stereochemistry shown in Table 1 and in the schemes. The rationale for this comes from the chemical shift of the proton at the C9 position in the formerly reported [3] diastereomeric compounds 1*R*,5*R*,9R- and 1*R*,5*R*,9S-9-hydroxy-5-(3-hydroxyphenyl-2-phenylethyl-2-azabicyclo[3.3.1]nonane, with stereochemistry determined by X-ray crystallography. The C9 hydroxyl group in the 1*R*,5*R*,9R compound was *trans* to the tertiary amine, and the C9 proton was found at 4.40 ppm. In comparison, in the diastereomeric 1*R*,5*R*,9S compound, where the C9 hydroxyl group was *cis* to the tertiary amine, the C9 proton was found upfield at 4.06 ppm. A similar effect was seen with the C9 *trans* and *cis* amino-containing compounds. When the C9 NH_2_ group was *trans* to the tertiary amine, the C9 proton was found at 3.58 ppm (in compounds **22** and **23**), and when *cis*, it was found at 3.50 ppm (in compounds **3** and **6**). The *trans* and *cis* C9 cyanomethyl compounds followed the same pattern. When the C9 cyanomethyl was *trans* to the tertiary amine, the C9 proton was found at 3.43 ppm (compounds **34** and **35**), and when the C9 cyanomethyl group was *cis* to the tertiary amine, the C9 proton was upfield at 3.31 ppm (compounds **38** and **41**).

The stereochemistry of the starting materials, the 5-(3-methoxyphenyl)-9-oxo-2-phenylethyl-2-azabicyclo[3.3.1]nonane (9-oxo phenylmorphans), has been established [15]. There is, then, only the stereochemistry of the C9 substituent to be determined. That became known via the X-ray spectroscopic data on compound **23** (Scheme 8). Compound **22** was its enantiomer—thus establishing the stereochemistry at C9 for compound **22**. The NMR chemical shifts of these known compounds were followed by all of the others. That is, the C9 substituent was *trans* to the nitrogen atom in the piperidine ring for the amino compounds in Scheme 8, and this gave us the chemical shift of the C9 proton in the *trans* series. The titanium reactions shown in Scheme 2 and Scheme 3 gave products that were *cis* for the C9 substituent and the nitrogen atom in the piperidine ring. This was established because the amino compounds **3** and **6** were different from **22** and **23**, and given the known stereochemistry of the starting material, they had to have the *cis* configuration. Similarly, for the cyanomethyl compounds, the *trans* configuration of **34** and **35** and the *cis* configuration of **38** and **41** were assigned tentatively based on the chemical shift of the C9 proton in the NMR. With these compounds, a clear and consistent correlation was found with the chemical shift of the C9 proton in the NMR. The exceptions to this were the methylaminomethyl compounds. The stereochemistry of these compounds was determined via their synthesis from the aldehyde in a series of reactions that led to known stereochemistry (proven by another X-ray [5]).

### 2.6. Synthesis of the Four C9 Cyanomethyl Diastereomers

**Scheme 9:**
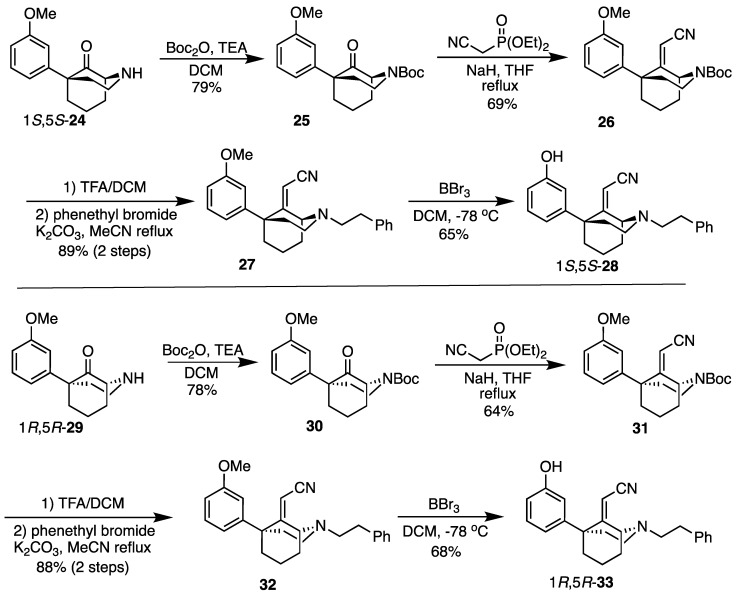
Synthesis of unsaturated nitrile enantiomers **28** and **33**.

To access the C9 cyanomethyl compounds, with the nitrogen atom at the end of the two-carbon chain derivative at C9, (1*S*,5*S*)-5-(3-methoxyphenyl)-2-methyl-2-azabicyclo[3.3.1]nonan-9-one [15] was N-demethylated to obtain 1*S*,5*S*-**24**. Subjecting the Boc-protected compound **25** to the Horner–Wadsworth–Emmons reaction gave rise to an unsaturated nitrile, **26**, that could be carried through Boc removal and alkylation to give **27**, followed by the O-demethylation of **27** to access the unsaturated nitrile **28**. Similar procedures using 1*S*,5*S*-**29** gave rise to the enantiomeric unsaturated nitrile **33** (Scheme 9).

**Scheme 10:**
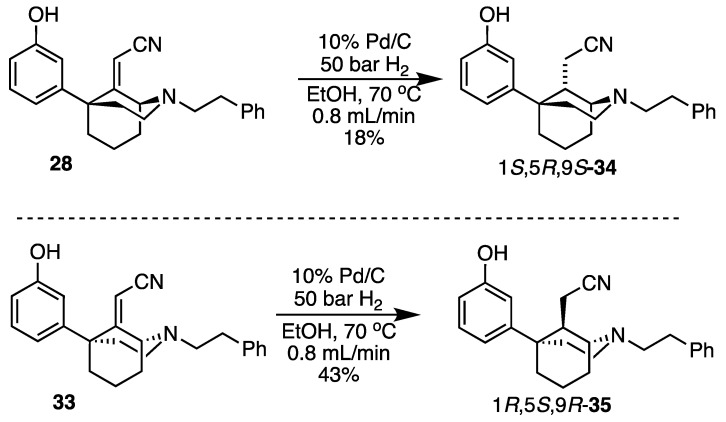
Reduction of unsaturated nitriles **28 and 33** to obtain the saturated cyanomethyl enantiomers 1*S*,5*R*,9*S*-**34** and 1*R*,5*S*,9*R*-**35**.

To obtain the saturated nitriles, we needed to reduce the alkene in **28** and **33** while leaving the nitrile intact. Employing traditional conditions with hydrogen and palladium on carbon as the catalyst resulted in a complex mixture of both alkene and partial nitrile reduction. However, using an H-cube hydrogenation flow reactor, we were able to develop conditions that gave the desired enantiomeric saturated nitriles 1*S*,5*R*,9*S*-**34** and 1*R*,5*S*,9*R*-**35** in low-to-moderate yield (Scheme 10).

**Scheme 11:**
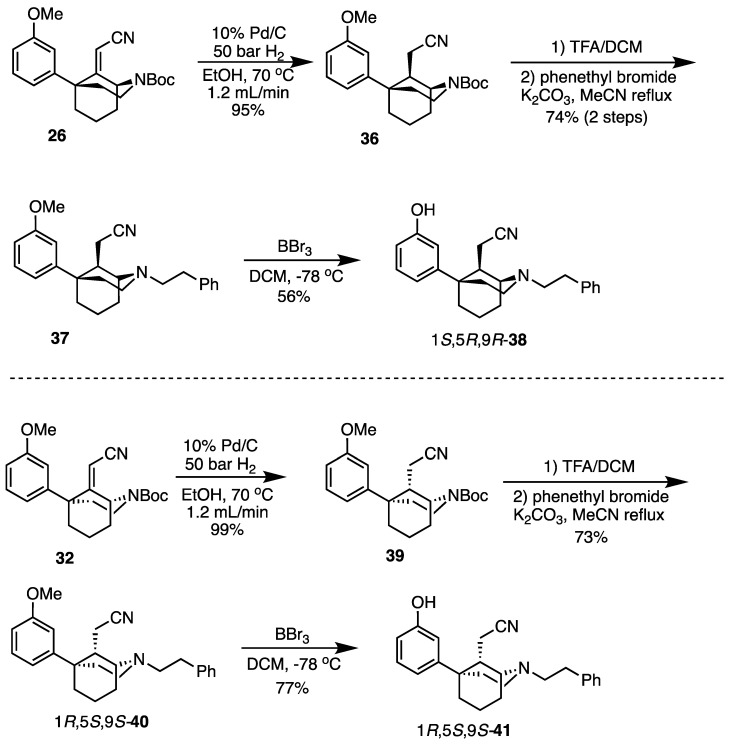
Synthesis of cyanomethyl enantiomers 1*S*,5*R*,9*R*-**38** and 1*R*,5*S*,9*S***-41**.

To access the enantiomers **38** and **41**, we used the Boc-protected amines **26** and **32** (Scheme 11).

The reduction of **26** using an H-cube hydrogenation flow reactor gave rise to the saturated nitrile **36** with the opposite stereochemistry at C9 to that seen in **34** (Scheme 10). The subsequent N-alkylation of **36** gave rise to the *N*-phenethyl ether **37**, and the O-demethylation of **37** provided the saturated cyanomethyl diastereomer 1*S*,5*R*,9*R*-**38**. Starting with **32**, the enantiomer of **26**, 1*R*,5*S*,9*S*-**41**, was obtained using similar procedures.

### 2.7. Synthesis of the C9 Methylaminomethyl Diastereomers

**Scheme 12:**
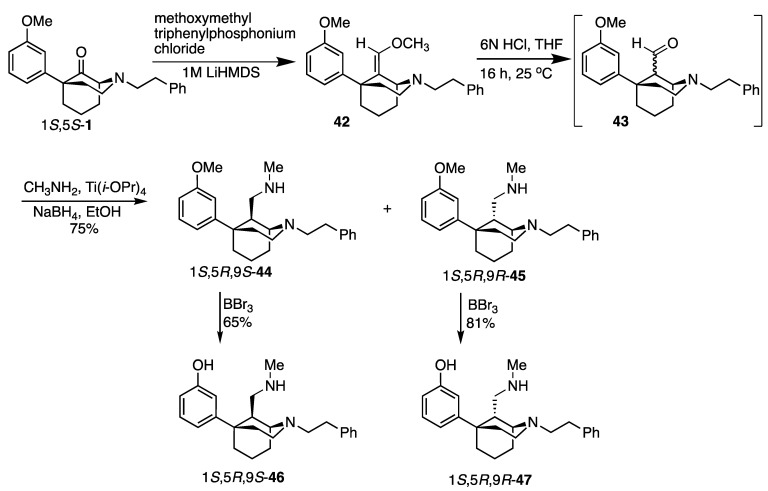
Synthesis of the 1*S*,5*R*,9*S*-C9 methylaminomethyl phenylmorphan (**46**) and 1*S*,5*R*,9*R*-C9 methylaminomethyl phenylmorphan (**47**) diastereomers.

Access to one pair of the C9 methylaminomethyl diastereomers was obtained from the *N*-phenylethyl ketone **1**. 1*S*,5*S*-**1** was subjected to a Witting reaction with methoxymethyl triphenylphosphonium chloride to give rise to the corresponding enol ether **42** (Scheme 12).

The two C9 methylaminomethyl analogs **44** and **45** were prepared by converting the enol ether **42** to the corresponding aldehyde **43** by treating it with 6 N HCl. The aldehyde **43** was not isolated. It was subjected to reductive amination with methylamine using titanium (IV) isopropoxide as the catalyst [16]. Sodium borohydride was added at 0 °C to reduce the formed imine. The reaction was carried out in a pressure vessel to prevent the methylamine from escaping. This resulted in a 1:1 diastereomeric mixture of the amines **44** and **45** with a 75% yield (Scheme 12). The chromatographic separation of the secondary amines and the O-demethylation of the phenolic ethers gave rise to the desired methylaminomethyl diastereomers 1*R*,5*S*,9*S*-**46** and 1*S*,5*R*,9*R*-**47** in 65% and 81% yields, respectively (Scheme 12).

**Scheme 13:**
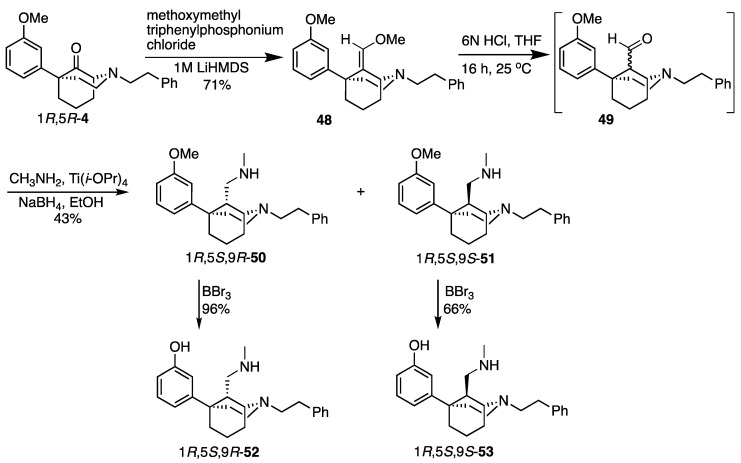
Synthesis of the 1*R*,5*S*,9*R*-C9 methylaminomethyl phenylmorphan (**52**) and 1*R*,5*S*,9*S*-C9 methylaminomethyl phenylmorphan (**53**) diastereomers, the enantiomers of **46** and **47**.

The enantiomers of **46** and **47** were prepared using the methods described above. The C9 secondary amines **50** and **51** were O-demethylated to give rise to 1*R*,5*S*,9*R-***52** and 1*R*,5*S*,9*S*-**53** in 96% and 66% yields, respectively (Scheme 13).

The assigned stereochemistry of the methylaminomethyl compounds came from the known stereochemistry of C9 analogs obtained from the same common aldehyde intermediate, **49**. The structure of the product of that reaction of the aldehyde was proven by X-ray crystallographic structure analysis [5,6].

### 2.8. Forskolin-Induced cAMP Accumulation Assay for In Vitro Determination of the Potency and Efficacy of the C9 Amino, Methylamino, Cyanomethyl, and Methylaminomethyl Compounds

Compounds were evaluated using the inhibition of the forskolin-induced *c*AMP accumulation assay: functional activity for *c*AMP (HitHunter Chinese hamster ovary cells (CHO-K1) that express the human μ-opioid receptor (OPRM1)) (Table 2).

The C9 amino compounds **3**, **6**, **22**, and **23** in Table 2 illustrate the importance of stereochemistry on MOR agonist activity. It is well known that enantiomers can greatly differ in their pharmacology, with one showing much more of an effect at the MOR than the other. Thus, 1*R*,5*R*,9*S*-**6** was a moderately potent and almost fully efficacious MOR agonist (MOR EC_50_ = 24 nM (% E_max_ = 91%)), and its enantiomer, 1*S*,5*S*,9*R*-**3** (MOR EC_50_ > 10,000 nM), had no effect as an agonist and little potency as an MOR antagonist (IC_50_ = 709 nM). Less difference was noted in the potencies and efficacies of the C9 amino enantiomeric pair 1*S*,5*S*,9*S*-**22** and 1*R*,5*R*,9*R*-**23**, where 1*R*,5*R*,9*R*-**23** was a moderately potent MOR partial agonist (EC_50_ = 26 nM (% E_max_ = 81%)) and was about three times as potent as 1*S*,5*S*,9*S*-**22**. The latter compound had much less potency as an MOR agonist and little efficacy (EC_50_ = 94 nM (%E_max_ = 53%)). There was a major difference in functional activity between 5-phenymorphans with a C9 amino moiety and those with a C9 hydroxyl substituent. The comparable chiral compound in the C9 hydroxy series, with 1*R*,5*R*,9*S* stereochemistry, was extremely potent in vitro and in vivo [3]. Although a direct comparison cannot be made because the amino compounds were examined using the cAMP assay and the hydroxy compounds were evaluated with MOR receptor binding in a [^35^S]GTPgS assay, it was obvious that the 1*R*,5*R*,9*S*-amino compound had much less potency and efficacy. Possibly the amino compound was less capable of the hydrogen bonding that apparently enabled the high potency of the hydroxy stereoisomer, as postulated through molecular modeling using energy-minimized structures [3].

An effect of the C9 substituent on activity could be clearly discerned in the C9 methylamino enantiomers 1*S*,5*S*,9*R*-**8** and 1*R*,5*R*,9*S*-**10**, where the former secondary amine was a weak antagonist with a high degree of antagonism (MOR IC_50_ = 178 nM (% I_max_ = 136%)), and the latter was a weak MOR agonist with slight efficacy (EC_50_ =83 nM (% E_max_ = 28%)).

The conversion of a primary amine at C9 in 1*S*,5*S*,9*R*-**3** to a secondary amine (1*S*,5*S*,9*R*-**8**) had little effect; both compounds were weak antagonists. However, the activity of a different secondary amine, the 1*R*,5*R*,9*S*-**10** stereochemical relative of the primary amine 1*R*,5*R*,9*S*-**6**, showed a clear difference in activity; the primary amine was more potent and efficacious than the secondary amine 1*R*,5*R*,9*S*-**10** (MOR EC_50_ = 83 nM (% E_max_ = 28%)). The secondary amine 1*R*,5*R*,9*S*-**10** had little efficacy as an MOR agonist. Obviously, the conversion to a secondary amine was not advantageous.

Unlike the secondary amines **8** and **10**, the enantiomeric oximes 1*S*,5*S*-**15** and 1*R*,5*R*-**17** were almost fully efficacious, and the latter was about 10-fold more potent than 1*S*,5*S*-**15** (EC_50_ =22 nM (% E_max_ = 99%)) and (EC_50_ =229 nM (% E_max_ = 91%)) for **17** and **15**, respectively. The oxime 1*R*,5*R*-**17** was a moderately potent, fully efficacious MOR agonist. Very much like the oximes in efficacy, the unsaturated cyano enantiomers 1*S*,5*S*-**28** and 1*R*,5*R*-**33** were fully efficacious, and 1*R*,5*R*-**33** was more than 10-fold more potent than 1*S*,5*S*-**28** and was about three times more potent than morphine (MOR EC_50_ = 1.8 nM (% E_max_ = 100%)) and (MOR EC_50_ = 27 nM (% E_max_ = 101%)) for **33** and **28**, respectively.

It was only among the saturated C9 cyano compounds that a potent partial MOR agonist was found. In the enantiomeric pair 1*S*,5*R*,9*R*-**38** and 1*R*,5*S*,9*S*-**41**, the former compound, 1*S*,5*R*,9*R*-**38**, was about twice as potent as morphine and had efficacy at or below 90% in this assay, making it a potentially interesting partial agonist (EC_50_ = 2.5 nM (% E_max_ = 89.6%)). 1*S*,5*R*,9*R*-**38** was a selective MOR agonist, having only little potency as a DOR partial agonist and KOR antagonist. Again, stereochemistry dictated agonist activity, and 1*R*,5*S*,9*S*-**41**, the enantiomer of 1*S*,5*R*,9*R*-**38**, was a weak antagonist (IC_50_ = 318 nM). In the enantiomeric pair 1*S*,5*R*,9*S*-**34** and 1*R*,5*S*,9*R*-**35**, both were fully efficacious, and 1*R*,5*S*,9*R*-**35** was the most potent compound of all the C9-nitrogen-containing compounds. It had nanomolar potency and was about seven times more potent than morphine (EC_50_ = 27 nM (% E_max_ = 101%)) and (EC_50_ = 0.85 nM (% E_max_ = 99%)) for **34** and **35**, respectively. It was uncommon in this series of compounds to find one diastereomer with no potency as an MOR agonist (1*R*,5*S*,9*S*-**41**), but that is not unusual among opioid enantiomers, where one of the enantiomers is found to have all of the potency and the other displays little or no potency at the MOR (e.g., (−) and (+)-naloxone) [18].

Three compounds exhibited weak MOR antagonist activity (the primary amine 1*S*,5*S*,9*R*-**3**, the secondary amine 1*S*,5*S*,9*R*-**8**, and the cyanomethyl compound 1*R*,5*S*,9*S*-**41**). One compound, the methylaminomethyl compound 1*S*,5*R*,9*S*-**46**, had more than a negligible effect at the KOR. It was a moderately potent full-efficacy KOR agonist (KOR EC_50_ = 18 nM (%E_max_ = 103%)). Two of the compounds, the unsaturated cyano compound 1*R*,5*R*-**33** and the saturated cyano compound 1*R*,5*S*,9*R*-**35**, were found to be potent at the DOR (DOR EC_50_ = 8 and 4 nM, respectively), but neither of them were efficacious (DOR %E_max_ = 69% and 35%, respectively).

Lastly, among the four diastereomers of the C9-methylaminomethyl-substituted compounds, the diastereomers 1*S*,5*R*,9*S*-**46** and 1*S*,5*R*,9*R*-**47** were both more potent than morphine and were fully efficacious (EC_50_ = 2.4 nM (%E_max_ = 103%)) and (EC_50_ = 3.7 nM (%E_max_ = 102%)) for 1*S*,5*R*,9*S*-**46** and 1*S*,5*R*,9*R***-47**, respectively. The remaining two diastereomers, 1*R*,5*S*,9*R*-**52** and 1*R*,5*S*,9*S*-**53**, were almost fully efficacious and had less potency at the MOR (EC_50_ =43 nM (%E_max_ = 94%)) and (EC_50_ = 16 nM (%E_max_ = 97%)), respectively. Except for 1*R*,5*S*,9*R*-**52**, all the methylaminomethy compounds were more potent and efficacious than the primary amines or the methylamines.

## 3. Materials and Methods

### 3.1. General Information

All reactions were performed in oven-dried glassware under an argon atmosphere, unless otherwise noted. Proton (^1^H NMR) and carbon (^13^C NMR) spectra were recorded on a Varian Gemini-400 spectrometer at 400 MHz for ^1^H NMR and 101 MHz for ^13^C NMR. The spectra have been shown in the Supplementary Materials. Mass spectra (HMS) were recorded on a Waters (Mitford, MA, USA) Xevo G2-XS QTof. Ions were produced using positive ion electrospray (ESI) at a capillary voltage of 2.8 KV. The ESI source temperature was 280 °C. The optical rotation data were obtained on a PerkinElmer polarimeter model 341, and melting points were obtained using Thomas Hoover capillary melting point (mp) apparatus. Thin-layer chromatography (TLC) was performed on a 250 mm Analtech GHLF. Visualization was accomplished under UV or by staining in an iodine chamber. Flash column chromatography was performed with Fluka silica gel 60 (mesh 220–400). The solvents used were CHCl_3_ and CMA (CHCl_3_:MeOH:NH4OH (50:45:5)), usually using a gradient of 0 → 10% or 15% CMA/CHCl_3_, or hexane and ethyl acetate at various gradients. Elemental analyses were performed by Robertson Microlit Laboratories, NJ, USA.

### 3.2. Synthesis

*(1S,5S,9R)-5-(3-Methoxyphenyl)-2-phenethyl-2-azabicyclo[3.3.1]nonan-9-amine* (**2**). In a dry flask, the ketone **1** (1.80 g, 5.15 mmol, 1 equiv), NH_4_OH (26 mL of a 2 M solution in EtOH, 51.5 mmol, 10 equiv), and Ti(O*i*Pr)_4_ (3.00 mL, 10.3 mmol, 2 equiv) were combined and stirred overnight at room temperature. The following morning, NaBH_4_ (292 mg, 7.73 mmol, 1.5 equiv) was added, and the reaction was stirred at room temperature for 1 h before it was quenched with NH_4_OH and filtered through celite with EtOAc. The filtrate was concentrated, and the residue was purified by flash chromatography with 0 → 10% CMA/CHCl_3_ to produce **2** as a colorless oil (1.20 g, 66%). ^1^H NMR (400 MHz; CDCl_3_): δ 7.27–7.18 (m, 6H), 7.03–6.98 (m, 2H), 6.74–6.72 (m, 1H), 3.76 (s, 3H), 3.56 (s, 1H), 3.05–2.85 (m, 7H), 2.19 (bs, 2H), 1.96–1.51 (m, 8H). ^13^C NMR (101 MHz; CDCl_3_): δ 159.77, 150.25, 140.32, 129.42, 128.74, 128.38, 126.07, 118.07, 112.24, 110.77, 57.64, 57.51, 55.32, 55.13, 49.70, 39.98, 39.95, 34.37, 27.77, 21.66, 18.12. HRMS-ESI (*m/z*): [M+H]^+^ calcd for C_23_H_31_N_2_O: 351.2436, found: 351.2434, [a]^20^_D_ +31.7° (*c* 1.04, CHCl_3_).

*3-((1S,5S,9R)-9-Amino-2-phenethyl-2-azabicyclo[3.3.1]nonan-5-yl)phenol* (**3**). In a dry flask, the ether **2** (200 mg, 0.571 mmol, 1 equiv) was dissolved in dichloromethane (13 mL) and cooled to −78 °C before BBr_3_ (274.6 mL, 2.85 mmol, 5 equiv) was added. After stirring the reaction for 15 min, the cooling bath was removed, and the contents were stirred for an additional 2 h at room temperature. The solvent was removed under vacuum conditions, and the residue was taken up in 1 N HCl and heated to reflux for 1 h. After cooling to room temperature, the pH was adjusted to 8 with NH_4_OH. The aqueous solution was extracted with dichloromethane (3x). The organic layers were dried over Na_2_SO_4_, filtered, and concentrated. The residue was purified by flash chromatography with 2 → 15% CMA/CHCl_3_, which produced **3** as a white foam (147 mg, 77%). The HCl salt was prepared by dissolving the solid (40 mg) in 400 μL of dichloromethane and adding 2 N HCl (4 equiv) in Et_2_O, resulting in a white solid (24 mg, 50% recovery). ^1^H-NMR (400 MHz; CDCl_3_): δ 7.29–6.82 (m, 6H), 6.79–6.70 (m, 2H), 6.69–6.68 (m, 1H), 3.50 (s, 1H), 3.12–2.95 (m, 3H), 2.85–2.74 (m, 4H), 2.33–2.21 (m, 2H), 2.10 (dd, *J* = 13.6, 4.1 Hz, 1H), 1.96–1.84 (m, 2H), 1.77–1.62 (m, 2H), 1.51–1.48 (m, 1H). ^13^C-NMR (101 MHz; CDCl_3_): δ 158.31, 149.57, 140.62, 130.39, 128.84, 128.46, 126.06, 116.70, 114.47, 111.41, 56.77, 56.27, 55.11, 48.50, 42.36, 40.87, 34.66, 29.04, 26.12, 23.31. HRMS-ESI (*m/z*): [M+H]^+^ calcd for C_22_H_29_N_2_O: calc: 337.2280, found: 337.2285, mp 70–73 °C, [α]^20^_D_ +8.74° (*c* 1.12, CHCl_3_), CHN calc. for C_22_H_30_Cl_2_N_2_O · 1 H_2_O: C, 61.82%; H, 7.55%; N, 6.55%; found: C, 61.50%; H, 7.24%; N, 6.30%.

*(1R,5R,9S)-5-(3-Methoxyphenyl)-2-phenethyl-2-azabicyclo[3.3.1]nonan-9-amine* (**5**). Using the ketone 1*R*,4*R*-**4**, the procedure followed the preparation of **2** (on a 473 mg scale) to obtain **5** as a yellow oil (235 mg, 50%). ^1^H-NMR (400 MHz; CDCl_3_): δ 7.28–7.14 (m, 6H), 6.95 (d, *J* = 7.9 Hz, 1H), 6.90 (t, *J* = 1.9 Hz, 1H), 6.73 (dd, *J* = 8.1, 2.3 Hz, 1H), 3.79 (s, 3H), 3.25 (d, *J* = 1.9 Hz, 1H), 3.06–2.84 (m, 3H), 2.84–2.76 (m, 4H), 2.36–2.25 (m, 2H), 1.98 (dd, *J* = 13.6, 4.9 Hz, 1H), 1.92–1.70 (m, 3H), 1.65–1.53 (m, 4H). ^13^C-NMR (101 MHz; CDCl_3_): δ 159.83, 150.97, 140.83, 129.48, 128.76, 128.28, 125.89, 117.99, 112.29, 110.52, 57.41, 56.81, 55.36, 55.19, 48.55, 41.49, 41.29, 34.64, 28.91, 25.76, 23.21. HRMS-ESI (*m/z*): [M+H]^+^ calcd for C_23_H_30_N_2_O: 351.2436, found: 251.2441, [α]^20^_D_ −31.3° (*c* 1.10, CHCl_3_).

*3-((1R,5R,9S)-9-Amino-2-phenethyl-2-azabicyclo[3.3.1]nonan-5-yl)phenol* (**6**). Using the ether **5**, the procedure followed the preparation of **3** on a 353 mg scale, obtaining the phenol as a white foam (233 mg, 66%). Salt formation to a hydrochloride produced a white solid (223 mg, 86% recovery). ^1^H-NMR (400 MHz; CDCl_3_): δ 7.29–6.82 (m, 6H), 6.79–6.70 (m, 2H), 6.69–6.68 (m, 1H), 3.50 (s, 1H), 3.12–2.95 (m, 3H), 2.85–2.74 (m, 4H), 2.33–2.21 (m, 2H), 2.10 (dd, *J* = 13.6, 4.1 Hz, 1H), 1.96–1.84 (m, 2H), 1.77–1.62 (m, 2H), 1.51–1.48 (m, 1H). ^13^C-NMR (101 MHz; CDCl_3_): δ 158.31, 149.57, 140.62, 130.39, 128.84, 128.46, 126.06, 116.70, 114.47, 111.41, 56.77, 56.27, 55.11, 48.50, 42.36, 40.87, 34.66, 29.04, 26.12, 23.31. HRMS-ESI (*m/z*): [M+H]^+^ calcd for C_22_H_29_N_2_O: 337.2280, found: 337.2284, mp 71–73 °C, [α]^20^_D_ −10.6° (*c* 1.08, CHCl_3_), CHN calc. for C_22_H_30_Cl_2_N_2_O · 0.25 H_2_O: C, 63.84%; H, 7.43%; N, 6.77%; found: C, 63.77%; H, 7.22%; N, 6.63%.

*(1S,5S,9R)-5-(3-Methoxyphenyl)-N-methyl-2-phenethyl-2-azabicyclo[3.3.1]nonan-9-amine* (**7**). In a pressure flask, the oxide **1** (300 mg, 0.858 mmol, 1 equiv), MeNH_2_·HCl (232 mg, 3.42 mmol, 4 equiv), triethylamine (480 mL, 3.43 mmol, 4 equiv), Ti(O*i*Pr)_4_ (1.01 mL, 3.42 mmol, 4 equiv), and EtOH (3 mL) were combined and heated to 50 °C overnight. The reaction was then cooled to room temperature, and NaBH_4_ (49 mg, 1.29 mmol, 1.5 equiv) was added. The mixture was stirred for 1 h at room temperature. The reaction was quenched with NH_4_OH and filtered through celite with EtOAc, and the filtrate was concentrated. The residue was purified by chromatography with 0 → 15% CMA/CHCl_3_ to produce **7** as a yellow oil (245 mg, 78%). ^1^H NMR (400 MHz; CDCl_3_): δ 7.27–7.14 (m, 6H), 6.92 (d, *J* = 7.9 Hz, 1H), 6.87 (t, *J* = 2.0 Hz, 1H), 6.71–6.68 (m, 1H), 3.78 (s, 3H), 3.12–3.03 (m, 3H), 2.98–2.75 (m, 5H), 2.35–2.24 (m, 2H), 2.20 (s, 3H), 2.04–1.99 (m, 1H), 1.91–1.69 (m, 3H), 1.65–1.58 (m, 1H), 1.48–1.44 (m, 1H). ^13^C-NMR (101 MHz; CDCl_3_): δ 159.51, 151.57, 140.85, 129.12, 128.65, 128.20, 125.81, 117.71, 111.97, 110.16, 63.84, 56.57, 55.08, 52.47, 48.67, 42.42, 40.49, 34.89, 34.31, 29.81, 25.92, 23.19. HRMS-ESI (*m/z*): [M+H]^+^ calcd for C_24_H_33_N_2_O: 365.2593, found: 365.2588, [α]^20^_D_ +15.1° (*c* 1.01, CHCl_3_).

*3-((1S,5S,9R)-9-(Methylamino)-2-phenethyl-2-azabicyclo[3.3.1]nonan-5-yl)phenol* (**8**). In a dry flask, ether **7** (245 mg, 0.672 mmol, 1 equiv) was dissolved in dichloromethane (15 mL) and cooled to −78 °C before BBr_3_ (320 uL, 3.36 mmol, 5 equiv) was added dropwise. After the reaction was stirred at −78 °C for 15 min, the cooling bath was removed, and the contents were allowed to stir at room temperature for 2 h. The solvent was removed in vacuo, and the residue was taken up in 1 N HCl (10 mL) and heated to reflux for 1 h. After cooling to room temperature, the pH was adjusted to 8 with NH_4_OH, and the aqueous mixture was extracted with CHCl_3_ (3x). The combined organic layers were dried over Na_2_SO_4_, filtered, and concentrated. The crude material was purified by flash chromatography with 2 → 20% CMA/CHCl_3_ to produce **8** as a yellow oil, which solidified upon drying (89 mg, 38%). The HCl salt was prepared by dissolving the free base (80 mg) in a minimal amount of dichloromethane and adding 2 M HCl (4 equiv) in Et_2_O, resulting in a pink solid (77 mg, 80% recovery). ^1^H NMR (400 MHz; CDCl_3_): δ 7.29–7.15 (m, 5H), 6.98 (t, *J* = 7.9 Hz, 1H), 6.73 (d, *J* = 7.7 Hz, 1H), 6.53 (s, 1H), 6.20–6.18 (m, 1H), 3.09–3.04 (m, 3H), 2.89–2.79 (m, 5H), 2.38–2.31 (m, 2H), 2.14 (s, 3H), 2.01–1.97 (m, 1H), 1.88–1.71 (m, 3H), 1.61–1.58 (m, 1H), 1.49–1.44 (m, 1H). ^13^C NMR (101 MHz; CDCl_3_): δ 157.28, 149.90, 140.79, 128.99, 128.74, 128.37, 126.06, 115.68, 114.04, 113.28, 64.61, 56.63, 53.19, 48.14, 42.45, 39.86, 34.41, 34.23, 29.49, 26.06, 23.09. HRMS-ESI (*m/z*): [M+H]+ calcd for C_23_H_31_N_2_O: 351.2436, found: 351.2430, mp 60–65 °C, [α]^20^_D_ +3.79° (*c* 1.12, CHCl_3_), CHN calc. for C_23_H_32_Cl_2_N_2_O · 1.38 H_2_O: C, 61.62%; H, 7.82%; N, 6.25%; found: C, 61.81%; H, 7.73%; N, 6.04%.

*(1R,5R,9S)-5-(3-Methoxyphenyl)-N-methyl-2-phenethyl-2-azabicyclo[3.3.1]nonan-9-amine* (**9**). Using the oxide **4**, the procedure followed the preparation of **7** on a 300 mg scale, obtaining **9** as a colorless oil (182 mg, 58%). ^1^H NMR (400 MHz; CDCl_3_): δ 7.27–7.14 (m, 6H), 6.92 (d, *J* = 7.9 Hz, 1H), 6.87 (t, *J* = 2.0 Hz, 1H), 6.71–6.68 (m, 1H), 3.78 (s, 3H), 3.12–3.03 (m, 3H), 2.98–2.75 (m, 5H), 2.35–2.24 (m, 2H), 2.20 (s, 3H), 2.04–1.99 (m, 1H), 1.91–1.69 (m, 3H), 1.65–1.58 (m, 1H), 1.48–1.44 (m, 1H). ^13^C-NMR (101 MHz; CDCl_3_): δ 159.51, 151.57, 140.85, 129.12, 128.65, 128.20, 125.81, 117.71, 111.97, 110.16, 63.84, 56.57, 55.08, 52.47, 48.67, 42.42, 40.49, 34.89, 34.31, 29.81, 25.92, 23.19. HRMS-ESI (*m/z*): [M+H]^+^ calcd for C_24_H_33_N_2_O: 365.2593, found: 365.2587, [α]^20^_D_ −19.1° (*c* 0.99, CHCl_3_).

*3-((1R,5R,9S)-9-(Methylamino)-2-phenethyl-2-azabicyclo[3.3.1]nonan-5-yl)phenol* (**10**). Using the ether **9**, the procedure followed the preparation of **8** on a 148 mg scale, obtaining an off-white foam (54 mg, 38%). Hydrochloride salt formation produced a white solid (55 mg, 85% recovery). ^1^H NMR (400 MHz; CDCl_3_): δ 7.29–7.15 (m, 5H), 6.98 (t, *J* = 7.9 Hz, 1H), 6.73 (d, *J* = 7.7 Hz, 1H), 6.53 (s, 1H), 6.20–6.18 (m, 1H), 3.09–3.04 (m, 3H), 2.89–2.79 (m, 5H), 2.38–2.31 (m, 2H), 2.14 (s, 3H), 2.01–1.97 (m, 1H), 1.88–1.71 (m, 3H), 1.61–1.58 (m, 1H), 1.49–1.44 (m, 1H). ^13^C NMR (101 MHz; CDCl_3_): δ 157.28, 149.90, 140.79, 128.99, 128.74, 128.37, 126.06, 115.68, 114.04, 113.28, 64.61, 56.63, 53.19, 48.14, 42.45, 39.86, 34.41, 34.23, 29.49, 26.06, 23.09. HRMS-ESI (*m/z*): [M+H]^+^ calcd for C_23_H_31_N_2_O: 351.2436, found: 351.2433, mp 62–65 °C, [α]^20^_D_ −1.86° (*c* 1.01, CHCl_3_), CHN calc. for C_23_H_32_Cl_2_N_2_O · 0.8 H_2_O: C, 63.09%; H, 7.74%; N, 6.40%; found: C, 63.09%; H, 7.55%; N, 6.22%.

*(1S,5S)-9-Hydrazineyl-5-(3-methoxyphenyl)-2-phenethyl-2-azabicyclo[3.3.1]nonane* (**11**). The combined oxide **1** (100 mg, 0.286 mmol, 1 equiv), hydrazine hydrate (28 mL, 0.572 mmol, 2 equiv), acetic acid (8.2 mL, 0.143 mmol, 0.5 eq) and benzene (4 mL) were combined in a flask. The flask was heated to reflux under Dean–Stark conditions overnight. The following day, TLC showed no starting material. After cooling to room temperature, the reaction was concentrated; the residue was taken up in EtOH (4 mL); and NaBH_4_ was added (11 mg, 0.286 mmol, 1 equiv). Upon the addition of the reductant, hydrogen gas evolution was observed, and once this ceased, the reaction was allowed to stir for 30 min longer to ensure the complete reduction of the hydrazone. The reaction was quenched with NH_4_OH and filtered through celite with EtOH, and the filtrate was concentrated into a yellow oil. It was used directly in the next step without purification. Hydrazine **11** did not react with titanium trichloride in basic media. ^1^H-NMR (400 MHz; CDCl_3_): δ 7.31–7.12 (m, 5H), 6.98–6.94 (m, 2H), 6.61 (dd, *J* = 8.1, 2.4 Hz, 1H), 4.24 (t, *J* = 3.3 Hz, 1H), 3.66 (s, 3H), 3.19–3.14 (m, 1H), 2.76–2.72 (m, 3H), 2.63–2.58 (m, 2H), 2.52–2.45 (m, 1H), 2.22–2.13 (m, 5H), 1.99 (d, *J* = 13.7 Hz, 1H), 1.67–1.63 (m, 1H), 1.48–1.43 (m, 1H).

*(1R,5R,9S)-5-(3-Methoxyphenyl)-2-phenethyl-2-azabicyclo[3.3.1]nonan-9-ol* (**12**) [3]. The compound **4** (500 mg, 1.43 mmol, 1 equiv) was dissolved in anhydrous THF (12.5 mL) in a flame-dried flask. After cooling to −78 °C, superhydride (2.16 mL of a 1 M solution, 2.16 mmol, 1.51 equiv) was added dropwise. After stirring for 1 h, TLC showed no remaining starting material. The mixture was quenched with MeOH, concentrated, and purified by chromatography with 0 to 2% MeOH/dichloromethane, yielding the alcohol **12** as a yellow oil (416 mg, 83%). Characterization data agreed with the literature. The product was used in the next step without further purification.

*(1R,5R,9S)-5-(3-Methoxyphenyl)-2-phenethyl-2-azabicyclo[3.3.1]nonan-9-yl methanesulfonate* (**13**). We dissolved **12** in dichloromethane (4 mL) in a dry flask and cooled the contents to 0 °C before adding TEA (181 mL, 1.30 mmol, 1.1 equiv), followed by MsCl (138 mL, 1.78 mmol, 1.5 equiv). After stirring for 1 h at room temperature, TLC showed no starting material. The reaction was quenched with H_2_O; the aqueous layer was extracted with dichloromethane (3x); and the combined organic layers were washed with NaHCO_3_ and dried over Na_2_SO_4_, filtered, and concentrated into an orange oil. The product **13** was used in the next step without further purification. ^1^H-NMR (400 MHz; CDCl_3_): δ 7.29–7.16 (m, 6H), 7.00 (d, *J* = 7.9 Hz, 1H), 6.95 (t, *J* = 1.8 Hz, 1H), 6.75 (dd, *J* = 8.1, 2.3 Hz, 1H), 5.12 (d, *J* = 2.5 Hz, 1H), 3.81 (s, 3H), 3.46 (d, *J* = 8.1, 2.4 Hz, 1H), 3.16–3.13 (m, 2H), 2.89–2.78 (m, 4H), 2.57–2.49 (m, 1H), 2.41 (s, 3H), 2.36–2.30 (m, 1H), 2.11–2.08 (m, 1H), 1.98–1.85 (m, 2H), 1.67–1.60 (m, 3H). ^13^C NMR (101 MHz; CDCl_3_): δ 159.61, 148.41, 140.56, 129.29, 128.78, 128.29, 125.94, 117.82, 111.98, 111.28, 84.68, 56.81, 56.11, 55.23, 48.20, 41.18, 39.80, 38.59, 34.12, 29.54, 25.69, 21.91.

***(****1S,5S)-5-(3-Hydroxyphenyl)-2-phenethyl-2-azabicyclo[3.3.1]nonan-9-one* (**14**). In a dry flask, ketone **1** (250 mg, 0.743 mmol, 1 equiv) was dissolved in dichloromethane (17 mL), and the solution was cooled to –78 °C. To this mixture was added BBr_3_ (360 mL, 3.71 mmol, 5 equiv), and the reaction was stirred for 15 min before the cooling bath was removed. After 2 h at room temperature, the reaction was analyzed by TLC (5% CMA/CHCl_3_), and no starting material was present. The solvent was removed in vacuo, and the residue was taken up in 1 N HCl (10 mL) and heated to reflux for 1 h. After cooling to room temperature, the pH was adjusted to 8 with NH_4_OH, and the aqueous solution was extracted with CHCl_3_ (3x). The combined organic layers were dried over Na_2_SO_4_, filtered, and concentrated. The crude product was purified by flash chromatography with 0 → 5% CMA/CHCl_3_ to produce **14** as a yellow solid (145 mg, 58%). Characterization data agreed with the literature [4].

*(1S,5S)-5-(3-Hydroxyphenyl)-2-phenethyl-2-azabicyclo[3.3.1]nonan-9-one oxime* (**15**). In a flask, **14** (145 mg, 0.432 mmol, 1 equiv), NaOAc (71.0 mg, 0.519 mmol, 1.2 equiv), hydroxylamine hydrochloride (36.0 mg, 0.519 mmol, 1.2 equiv), and MeOH (4.3 mL) were combined and heated to reflux for 2 h. After cooling to room temperature, the reaction was quenched with saturated aqueous NaHCO_3_ and extracted with EtOAc (3x). The combined organic layers were dried over Na_2_SO_4_, filtered, and concentrated. The crude product **15** was purified by flash chromatography with 2 → 15% CMA/CHCl_3_ to produce a white solid (119 mg, 79%). The HCl salt was prepared by dissolving the free base (90 mg) in minimal EtOAc and adding 2 M HCl in Et_2_O (2 equiv), resulting in a white solid (70 mg, 70% recovery). ^1^H NMR (400 MHz; DMSO-d_6_): δ 10.57 (s, 1H), 9.12 (s, 1H), 7.30–7.24 (m, 4H), 7.20–7.16 (m, 1H), 7.06–7.02 (m, 1H), 6.75–6.74 (m, 2H), 6.56–6.54 (m, 1H), 4.42–4.41 (m, 1H), 3.12–2.83 (m, 1H), 2.83–2.64 (m, 5H), 2.29–2.23 (m, 1H), 2.18–2.11 (m, 1H), 2.09–1.98 (m, 3H), 1.97–1.89 (m, 1H), 1.59–1.48 (m, 1H), 1.41–1.37 (m, 1H). ^13^C NMR (101 MHz; DMSO-d_6_): δ 162.20, 156.88, 148.58, 140.85, 129.09, 128.62, 128.48, 126.23, 118.23, 115.38, 112.92, 58.66, 56.47, 51.36, 48.44, 43.90, 38.89, 34.22, 30.55, 19.77. HRMS-ESI (*m/z*): [M+H]^+^ calcd for C_22_H_27_N_2_O_2_: 351.2073, found: 351.2075, mp 206–208 °C, [α]^20^_D_ +26.2° (*c* 1.02, MeOH). CHN calc. for C_22_H_27_ClN_2_O_2_ · 0.7 H_2_O: C, 66.14%; H, 7.16%; N, 7.01%; found C, 66.25%; H, 6.97%; N, 6.76%.

*(1R,5R)-5-(3-Hydroxyphenyl)-2-phenethyl-2-azabicyclo[3.3.1]nonan-9-one* (**16**). Using **4**, the procedure followed that of **14** on a 2.01 g scale to obtain **16** as a yellow oil (664 mg, 34%). Characterization data agreed with the literature [3].

*(1R,5R)-5-(3-Hydroxyphenyl)-2-phenethyl-2-azabicyclo[3.3.1]nonan-9-one oxime* (**17**). Using **16**, the procedure followed that of **14** on a 664 mg scale to obtain **17** as a white solid (549 mg, 79%). The HCl salt was prepared by dissolving the free base (200 mg) in minimal EtOAc and adding 2 M HCl in Et_2_O (2 equiv), resulting in a white solid (121 mg, 55% recovery). ^1^H NMR (400 MHz; DMSO-d_6_): δ 10.57 (s, 1H), 9.12 (s, 1H), 7.30–7.24 (m, 4H), 7.20–7.16 (m, 1H), 7.06–7.02 (m, 1H), 6.75–6.74 (m, 2H), 6.56–6.54 (m, 1H), 4.42–4.41 (m, 1H), 3.12–2.83 (m, 1H), 2.83–2.64 (m, 5H), 2.29–2.23 (m, 1H), 2.18–2.11 (m, 1H), 2.09–1.98 (m, 3H), 1.97–1.89 (m, 1H), 1.59–1.48 (m, 1H), 1.41–1.37 (m, 1H). ^13^C NMR (101 MHz; DMSO-d_6_): δ 162.20, 156.88, 148.58, 140.85, 129.09, 128.62, 128.48, 126.23, 118.23, 115.38, 112.92, 58.66, 56.47, 51.36, 48.44, 43.90, 38.89, 34.22, 30.55, 19.77. HRMS-ESI (*m/z*): [M+H]^+^ calcd for C_22_H_27_N_2_O_2_: 351.2073, found: 351.2076, mp 208–210 °C. CHN calc. for C_22_H_27_ClN_2_O_2_ · 0.25 H_2_O: C, 67.51%; H, 7.08%; N, 7.16%; found: C, 67.65%; H, 7.05%; N, 6.98%.

*(1S,5S)-5-(3-Methoxyphenyl)-2-phenethyl-2-azabicyclo[3.3.1]nonan-9-one oxime* (**18**). Using ketone **1**, the procedure followed that of compound **19** on a 1.34 g scale to obtain **18** as a white solid (1.18 g, 84%). ^1^H NMR (400 MHz; CDCl_3_): δ 7.89 (bs, 1H), 7.31–7.27 (m, 2H), 7.24–7.18 (m, 4H), 6.93 (d, *J* = 7.9 Hz, 1H), 6.89 (d, *J* = 2.0 Hz, 1H), 6.72 (dd, *J* = 8.1, 2.4 Hz, 1H), 4.54 (bs, 1H), 3.74 (s, 3H), 3.20 (bs, 1H), 2.86–2.74 (m, 5H), 2.45–2.39 (m, 1H), 2.23–2.02 (m, 5H), 1.69–1.66 (m, 1H), 1.60–1.55 (m, 1H). ^13^C NMR (101 MHz; CDCl_3_): δ 159.01, 147.31, 140.32, 128.78, 128.62, 128.34, 126.01, 119.84, 113.82, 110.95, 58.66, 55.13, 50.92, 48.62, 44.05, 39.27, 38.50, 34.30, 30.37, 19.64. HRMS-ESI (*m/z*): [M+H]^+^ calcd for C_23_H_29_N_2_O_2_: 365.2229, found: 365.2231, mp 122.9 °C, [α]^20^_D_ +31.6° (*c* 1.03, CHCl_3_).

*(1R,5R)-5-(3-Methoxyphenyl)-2-phenethyl-2-azabicyclo[3.3.1]nonan-9-one oxime* (**19**). Ketone **4** (361 mg, 1.03 mmol, 1 equiv), hydroxylamine hydrochloride (86.1 mg, 1.24 mmol, 1.2 equiv), and sodium acetate trihydrate (169 mg, 1.24 mmol, 1.2 equiv) were placed in a flask and suspended in MeOH (10 mL). The contents were heated to reflux, during which time all of the solids dissolved. After heating for 2 h, no starting material remained as seen through TLC. The reaction was cooled to room temperature and quenched with saturated aqueous NaHCO_3_, and the aqueous layer was extracted with dichloromethane (3x). The combined organic layers were dried over Na_2_SO_4_, filtered, and concentrated. The crude product was purified by flash chromatography with 40% EtOAc/hexane to produce **19** as a white solid (307 mg, 82%). ^1^H NMR (400 MHz; CDCl_3_): δ 8.35 (bs, 1H), 7.32–7.28 (m, 2H), 7.24–7.18 (m, 4H), 6.93 (d, *J* = 7.9 Hz, 1H), 6.89 (t, *J* = 2.0 Hz, 1H), 6.72 (dd, *J* = 8.1, 2.4 Hz, 1H), 4.51 (bs, 1H), 3.74 (s, 3H), 3.18–3.17 (m, 1H), 2.88–2.83 (m, 4H), 2.79–2.73 (m, 1H), 2.45–2.38 (m, 1H), 2.22–2.08 (m, 4H), 2.04–2.00 (m, 1H), 1.68–1.64 (m, 1H), 1.59–1.52 (m, 1H). ^13^C NMR (101 MHz; CDCl_3_): δ 164.60, 159.03, 147.33, 140.33, 128.79, 128.62, 128.34, 125.99, 119.83, 113.81, 110.97, 58.68, 55.13, 50.89, 48.61, 44.06, 39.27, 38.44, 34.32, 30.40, 19.63. HRMS-ESI (*m/z*): [M+H]^+^ calcd for C_23_H_29_N_2_O_2_: 365.2229, found: 365.2225, mp 119–121 °C, [α]^20^_D_ –30.7° (*c* 1.08, CHCl_3_).

*(1R,5R,9R)-5-(3-Methoxyphenyl)-2-phenethyl-2-azabicyclo[3.3.1]nonan-9-amine* (**20**). Using **19**, the procedure followed the preparation of **21** on a 1.09 g scale; the only change was in the reaction workup procedure. The reaction was cooled to 0 °C and diluted with Et_2_O, followed by the addition of H_2_O (460 mL), 15% NaOH (460 uL), and an additional portion of H_2_O (1.40 mL). After stirring for 15 min, 2.30 g of Na_2_SO_4_ · 10 H_2_O was added, and the contents were stirred for an additional 15 min. The solids were filtered off and washed with Et_2_O, and the filtrate was concentrated and purified as described previously to obtain **20** as a yellow oil (361 mg, 34%). ^1^H-NMR (400 MHz; CDCl_3_): δ 7.33–7.20 (m, 6H), 7.06–6.99 (m, 2H), 6.77–6.75 (m, 1H), 3.81 (s, 3H), 3.66 (bs, 1H), 3.13 (bs, 1H), 3.13–3.05 (m, 2H), 3.05–2.91 (m, 4H), 2.28–2.22 (m, 2H), 2.02–1.77 (m, 6H). ^13^C-NMR (101 MHz; CDCl_3_): δ 159.76, 150.36, 140.46, 129.41, 128.73, 128.36, 126.03, 118.08, 112.24, 110.74, 57.69, 57.46, 55.46, 55.15, 49.71, 40.09, 40.02, 34.50, 27.81, 21.70, 18.17. HRMS-ESI (*m/z*): [M+H]^+^ calcd for C_23_H_31_N_2_O: 351.2436, found: 351.2435, [α]^20^_D_ –37.3° (*c* 1.02, CHCl_3_).

*(1S,5S,9S)-5-(3-Methoxyphenyl)-2-phenethyl-2-azabicyclo[3.3.1]nonan-9-amine* (**21**). We dissolved the oxime **15** (292 mg, 0.801 mmol, 1 equiv) in anhydrous THF (15 mL) and added LAH (122 mg, 3.20 mmol, 4 equiv) gradually to avoid excessive foaming. The contents were heated to reflux overnight then cooled to room temperature the following day and quenched slowly with 1 N HCl to pH 1. The pH was then adjusted to 9 by the addition of NH_4_OH, and the suspension was filtered through celite with EtOAc. The filtrate was concentrated and purified by flash chromatography with a gradual gradient of 1 → 8% CMA/CHCl_3_ (the slower the gradient and the longer the column, the better the separation of the diastereomers) to produce **21** as a pale-yellow oil (105 mg, 37%).^1^H-NMR (400 MHz; CDCl_3_): δ 7.33–7.20 (m, 6H), 7.06–6.99 (m, 2H), 6.77–6.75 (m, 1H), 3.81 (s, 3H), 3.66 (bs, 1H), 3.13 (bs, 1H), 3.13–3.05 (m, 2H), 3.05–2.91 (m, 4H), 2.28–2.22 (m, 2H), 2.02–1.77 (m, 6H). ^13^C-NMR (101 MHz; CDCl_3_): δ 159.76, 150.36, 140.46, 129.41, 128.73, 128.36, 126.03, 118.08, 112.24, 110.74, 57.69, 57.46, 55.46, 55.15, 49.71, 40.09, 40.02, 34.50, 27.81, 21.70, 18.17. HRMS-ESI (*m/z*): [M+H]^+^ calcd for C_23_H_31_N_2_O: 351.2436, found: 351.2433, [α]^20^_D_ –32.4° (*c* 0.98, CHCl_3_).

*3-((1S,5S,9S)-9-Amino-2-phenethyl-2-azabicyclo[3.3.1]nonan-5-yl)phenol* (**22**). Using the ether **21**, the procedure followed the preparation of **23** on a 361 mg scale. The phenol **22** was obtained as a white foam (189 mg, 55%). Hydrochloride salt formation produced a white powder (208 mg, 90% recovery). ^1^H-NMR (400 MHz; CDCl_3_): δ 7.28–7.15 (m, 6H), 6.89–6.87 (m, 2H), 6.69–6.67 (m, 1H), 3.58 (d, *J* = 2.9 Hz, 1H), 3.14 (s, 1H), 3.02–2.99 (m, 2H), 2.89–2.80 (m, 4H), 2.25–2.11 (m, 2H), 1.98–1.74 (m, 5H), 1.68–1.62 (m, 1H). ^13^C-NMR (101 MHz; CDCl_3_): δ 157.33, 149.26, 139.79, 129.76, 128.72, 128.41, 126.15, 117.29, 114.33, 112.61, 57.51, 57.04, 55.00, 49.56, 39.53, 39.44, 33.78, 27.57, 21.28, 18.05. HRMS-ESI (*m/z*): [M+H]^+^ calcd for C_22_H_29_N_2_O: 337.2280, found: 337.2281, mp 72–73 °C, [α]^20^_D_ −21.4° (*c* 1.00, CHCl_3_).

*3-((1R,5R,9R)-9-Amino-2-phenethyl-2-azabicyclo[3.3.1]nonan-5-yl)phenol* (**23**). We dissolved the ether **20** (538 mg, 1.53 mmol, 1 equiv) in dichloromethane (9 mL) and cooled the solution to −78 °C before BBr_3_ (739 mL, 7.67 mmol, 5 equiv) was added. After 15 min, the cooling bath was removed, and the reaction was allowed to stir at room temperature for 2 h, at which point TLC showed no starting material. The reaction was quenched with MeOH, concentrated, and refluxed in 9 mL of 1 N HCl for 1 h to break up boron complexes. After cooling to room temperature, the pH was adjusted to 9 with NH_4_OH, and the aqueous layer was extracted with dichloromethane (3x). The organic layers were combined, dried over Na_2_SO_4_, filtered, and concentrated. Purification by flash chromatography with 2 → 15% CMA/CHCl_3_ afforded 142 mg (28% yield) of **23** as a white foam. A salt was made by dissolving 142 mg of the free base in a minimal amount of MeOH and adding concentrated HCl (2 equiv per amine, 4 equiv total). After the solution was stirred for 1 h, the solvent was removed under vacuum conditions, and the pasty material recrystallized from EtOH to yield **23** as a fluffy white solid (163 mg, 94% recovery). ^1^H-NMR (400 MHz; CDCl_3_): δ 7.28–7.15 (m, 6H), 6.89–6.87 (m, 2H), 6.69–6.67 (m, 1H), 3.58 (d, *J* = 2.9 Hz, 1H), 3.14 (s, 1H), 3.02–2.99 (m, 2H), 2.89–2.80 (m, 4H), 2.25–2.11 (m, 2H), 1.98–1.74 (m, 5H), 1.68–1.62 (m, 1H). ^13^C-NMR (101 MHz; CDCl_3_): δ 157.33, 149.26, 139.79, 129.76, 128.72, 128.41, 126.15, 117.29, 114.33, 112.61, 57.51, 57.04, 55.00, 49.56, 39.53, 39.44, 33.78, 27.57, 21.28, 18.05. HRMS-ESI (*m/z*): [M+H]^+^ calcd for C_22_H_29_N_2_O: 337.2280, found: 337.2280, mp 79.6–81.5 °C, [α]^20^_D_ +20.9° (*c* 1.01, CHCl_3_). CHN calc. for C_22_H_30_Cl_2_N_2_O · 3.8 H_2_O: C, 55.3%; H, 7.93%; N, 5.86%; found: C, 55.24%; H, 7.80%; N, 5.75%.

*(1S,5S)-5-(3-Methoxyphenyl)-2-azabicyclo[3.3.1]nonan-9-one* (**25**). In a flask, the secondary amine **24** [4] (3.32 g, 13.5 mmol, 1 equiv) and DMAP (165 mg, 1.35 mmol, 0.100 equiv) were dissolved in dichloromethane (34 mL). The contents were cooled to 0 °C, and TEA (2.83 mL, 20.3 mmol, 1.5 equiv) was added, followed by Boc_2_O (4.66 mL, 20.3 mmol, 1.5 equiv). After stirring overnight, a spatula tip’s worth of imidazole was added, and the solution was stirred for another 1 h to quench excess Boc_2_O. The contents were transferred to a separatory funnel and washed with dilute HCl and H_2_O; the aqueous layer was back extracted with dichloromethane (2x); and the organic layers dried over Na_2_SO_4_. The removal of the solvent in vacuo, followed by purification by flash chromatography with 0 to 40% EtOAc/hexane, produced **25** as a viscous yellow oil (3.69 g, 79%). ^1^H-NMR (400 MHz; CDCl_3_): δ 7.30–7.26 (m, 1H), 6.82–6.76 (m, 3H), 4.31 (bs, 1H), 4.24–4.19 (m, 1H), 3.80 (s, 3H), 3.22–3.15 (m, 1H), 2.56–2.48 (m, 2H), 2.39–2.31 (m, 2H), 2.21–2.18 (m, 2H), 1.80–1.72 (m, 2H), 1.49 (s, 9H). ^13^C-NMR (101 MHz; CDCl_3_): δ 211.81, 159.25, 154.62, 145.59, 129.05, 119.36, 113.52, 111.31, 80.30, 63.62, 55.09, 52.81, 40.98, 40.64, 38.09, 35.54, 28.39, 17.56. HRMS-ESI (*m/z*): [M+Na]^+^ calcd for C_20_H_27_NO_4_Na: 368.1838, found: 368.1833 [α]^20^_D_ −37.1° (*c* 1.08, CHCl_3_).

*tert-Butyl (1S,5S)-9-(cyanomethylene)-5-(3-methoxyphenyl)-2-azabicyclo[3.3.1]nonane-2-carboxylate* (**26**). To a flame-dried flask was added NaH (452 mg, 11.3 mmol, 3 equiv, 60% dispersion in mineral oil). The solid was washed with hexane 3 times under nitrogen conditions before THF (10 mL) was added. To this suspension, diethyl (cyanomethyl)phosphonate (1.83 mL, 11.3 mmol, 3 equiv) was added dropwise. Once gas evolution ceased and a clear solution formed, *N*-Boc ketone **25** (1.3 g, 3.76 mmol, 1 equiv) as a solution in THF (7 mL) was added, and the reaction mixture was heated to reflux overnight. After cooling to room temperature, EtOH was added dropwise to quench excess NaH, and the contents were concentrated in vacuo. The residual oil was purified by chromatography with 0 to 20% EtOAc/hexane to yield **26** as a white solid (961 mg, 69^1^H-NMR (400 MHz; CDCl_3_): δ 7.23 (t, *J* = 7.9 Hz, 1H), 6.78–6.74 (m, 3H), 5.11–5.05 (m, 1H), 4.55 (s, 1H), 4.03–3.94 (m, 1H), 3.74 (s, 3H), 3.14–3.08 (m, 1H), 2.37–2.34 (m, 1H), 2.24–2.20 (m, 2H), 2.06–1.97 (m, 3H), 1.67–1.66 (m, 1H), 1.46 (s, 10H). ^13^C-NMR (101 MHz; CDCl_3_): δ 171.58, 159.77, 155.04, 147.40, 129.75, 119.59, 116.06, 113.94, 111.56, 94.92, 80.61, 55.64, 55.38, 45.79, 40.55, 39.89, 38.45, 34.22, 28.59, 18.06. HRMS-ESI (*m/z*): [M+Na]^+^ calcd for C_22_H_28_N_2_O_3_Na: 391.1998, found: 391.1997, mp 124–126 °C, [α]^20^_D_ –44.3° (*c* 1.10, CHCl_3_).

*2-((1S,5S)-5-(3-methoxyphenyl)-2-phenethyl-2-azabicyclo[3.3.1]nonan-9-ylidene)acetonitrile* (**27**). A solution of **26** (500 mg, 1.36 mmol, 1 equiv) in dichloromethane (4.6 mL) was prepared and cooled to 0 °C before TFA (2.09 mL, 27.1 mmol, 20 equiv) was added dropwise. After 30 min, TLC showed no evidence of starting material. The pH was adjusted to 9 with NH_4_OH, and the aqueous layer was extracted with CHCl_3_ (3x). The combined organic layers were dried over Na_2_SO_4_, filtered, and concentrated to provide 2-((1*S*,5*S*)-5-(3-methoxyphenyl)-2-azabicyclo[3.3.1]nonan-9-ylidene)acetonitrile as a golden oil. This secondary amine was used directly in the next step without purification. ^1^H-NMR (400 MHz; CDCl_3_): δ 7.27–7.23 (m, 1H), 6.84 (d, *J* = 7.9 Hz, 1H), 6.80 (t, *J* = 2.0 Hz, 1H), 6.76 (dd, *J* = 8.1, 2.3 Hz, 1H), 4.58 (s, 1H), 4.29 (bs, 1H), 3.76 (s, 3H), 3.45–3.38 (m, 1H), 2.93 (dt, *J* = 13.1, 6.3 Hz, 1H), 2.37–2.22 (m, 3H), 2.15–2.07 (m, 4H), 1.88–1.78 (m, 1H), 1.75–1.69 (m, 1H). ^13^C-NMR (101 MHz; CDCl_3_): δ 175.31, 159.51, 147.07, 129.36, 119.51, 116.54, 113.80, 111.26, 92.18, 55.22, 53.91, 46.09, 41.81, 41.51, 39.51, 34.61, 20.04. HRMS-ESI (*m/z*): [M+H]^+^ calcd for C_17_H_21_N_2_O: 269.1654, found: 269.1653, [α]^20^_D_ −69.2° (*c* 1.11, CHCl_3_).

To a flask were added K_2_CO_3_ (375 mg, 2.71 mmol, 2 equiv), the secondary amine obtained above (364 mg, 1.36 mmol, 1 equiv), phenethyl bromide (278 mL, 2.03 mmol, 1.5 equiv), and MeCN (3.2 mL). The suspension was heated to reflux overnight, and the solids were filtered off and washed with MeCN. The filtrate was concentrated and purified by chromatography with 0 to 20% EtOAc/hexane to obtain the tertiary amine **27** as a colorless oil (449 mg, 89^1^H-NMR (400 MHz; CDCl_3_): δ 7.31–7.18 (m, 6H), 6.89–6.79 (m, 3H), 4.62 (s, 1H), 4.11–4.09 (m, 1H), 3.80 (s, 3H), 3.16–3.10 (m, 1H), 2.93–2.84 (m, 4H), 2.78–2.72 (m, 1H), 2.39–2.35 (m, 1H), 2.24–2.05 (m, 5H), 1.70–1.66 (m, 1H), 1.65–1.54 (m, 1H). ^13^C-NMR (101 MHz; CDCl_3_): δ 173.79, 159.51, 147.46, 140.15, 129.35, 128.77, 128.36, 126.03, 119.68, 116.75, 113.92, 111.27, 93.40, 60.44, 58.82, 55.24, 48.28, 45.79, 39.58, 38.77, 34.49, 32.58, 19.49. HRMS-ESI (*m/z*): [M+H]^+^ calcd for C_25_H_29_N_2_O: 373.2280, found: 373.2281, [α]^20^_D_ –11.6° (*c* 1.00, CHCl_3_).

*2-((1S,5S)-5-(3-Hydroxyphenyl)-2-phenethyl-2-azabicyclo[3.3.1]nonan-9-ylidene)acetonitrile* (**28**). A solution of **27** (782 mg, 2.10 mmol, 1 equiv) in dichloromethane (47 mL) was prepared and cooled to −78 °C before BBr_3_ (1.01 mL, 10.5 mmol, 5 equiv) was added dropwise. After 15 min, the cooling bath was removed, and the solution was allowed to stir at room temperature for an additional 2 h. The solvent was removed in vacuo, and the residue was taken up in H_2_O. The pH was adjusted to 9 with NH_4_OH, and the contents were stirred for 30 min to break up borane complexes. The aqueous layer was extracted with CHCl_3_ (3x), and the combined organic layers were dried over Na_2_SO_4_. The solids were filtered off, and the filtrate was concentrated then purified by chromatography using 0 to 20% EtOAc/hexane to produce a white foam (487 mg, 65%). Crystallization was obtained by dissolving 100 mg of product in minimal aq MeOH then adding 2 N HCl in Et_2_O (2 equiv) to produce **28** as a white solid (65 mg, 63% recovery). ^1^H-NMR (400 MHz; CDCl_3_): δ 7.32–7.19 (m, 6H), 6.86–6.84 (m, 1H), 6.77 (t, *J* = 2.1 Hz, 1H), 6.74 (dd, *J* = 7.8, 2.2 Hz, 1H), 4.65 (s, 1H), 4.13–4.12 (m, 1H), 3.19–3.13 (m, 1H), 2.94–2.85 (m, 4H), 2.81–2.74 (m1H), 2.42–2.36 (m, 1H), 2.24–2.08 (m, 5H), 1.72–1.63 (m, 1H), 1.61–1.57 (m, 1H). ^13^C-NMR (101 MHz; CDCl_3_): δ 173.76, 155.83, 147.79, 140.15, 129.72, 128.91, 128.54, 126.24, 119.66, 116.85, 114.65, 114.08, 93.65, 60.51, 58.89, 48.39, 45.86, 39.70, 38.71, 34.54, 32.47, 19.74. HRMS-ESI (*m/z*): [M+H]^+^ calcd for C_24_H_27_N_2_O: 359.2123, found: 359.2122, mp 152–153 °C, [α]^20^_D_ –11.0° (*c* 0.99, CHCl_3_). CHN calc. for C_24_H_27_ClN_2_O · 0.15 H_2_O: C, 72.49%; H, 6.92%; N, 7.04%; found C, 72.48%; H, 6.90%; N, 7.07%.

*tert-Butyl (1R,5R)-5-(3-methoxyphenyl)-9-oxo-2-azabicyclo[3.3.1]nonane-2-carboxylate* (**30**). The procedure followed that of **25** on a 3.88 g scale to obtain **30** as a yellow oil (4.26 g, 78%). ^1^H-NMR (400 MHz; CDCl_3_): δ 7.30–7.26 (m, 1H), 6.82–6.76 (m, 3H), 4.31 (bs, 1H), 4.24–4.19 (m, 1H), 3.80 (s, 3H), 3.22–3.15 (m, 1H), 2.56–2.48 (m, 2H), 2.39–2.31 (m, 2H), 2.21–2.18 (m, 2H), 1.80–1.72 (m, 2H), 1.49 (s, 9H). ^13^C-NMR (101 MHz; CDCl_3_): δ 211.81, 159.25, 154.62, 145.59, 129.05, 119.36, 113.52, 111.31, 80.30, 63.62, 55.09, 52.81, 40.98, 40.64, 38.09, 35.54, 28.39, 17.56. HRMS-ESI (m/z): [M+Na]^+^ calcd for C_20_H_27_NO_4_Na: 368.1838, found: 368.1833 [α]^20^_D_ +42.9° (*c* 1.13, CHCl_3_).

*tert-Butyl (1R,5R)-9-(cyanomethylene)-5-(3-methoxyphenyl)-2-azabicyclo[3.3.1]nonane-2-carboxylate* (**31**). The procedure followed that of **26** on a 4.26 g scale to obtain **31** as a white solid (2.91 g, 64%). ^1^H-NMR (400 MHz; CDCl_3_): δ 7.23 (t, *J* = 7.9 Hz, 1H), 6.78–6.74 (m, 3H), 5.11–5.05 (m, 1H), 4.55 (s, 1H), 4.03–3.94 (m, 1H), 3.74 (s, 3H), 3.14–3.08 (m, 1H), 2.37–2.34 (m, 1H), 2.24–2.20 (m, 2H), 2.06–1.97 (m, 3H), 1.67–1.66 (m, 1H), 1.46 (s, 10H). ^13^C-NMR (101 MHz; CDCl_3_): δ 171.58, 159.77, 155.04, 147.40, 129.75, 119.59, 116.06, 113.94, 111.56, 94.92, 80.61, 55.64, 55.38, 45.79, 40.55, 39.89, 38.45, 34.22, 28.59, 18.06. HRMS-ESI (*m/z*): [M+Na]^+^ calcd for C_22_H_28_N_2_O_3_Na: 391.1998, found: 391.1998, mp 123–126 °C, [α]^20^_D_ +44.0° (*c* 1.02, CHCl_3_).

*2-((1R,5R)-5-(3-methoxyphenyl)-2-phenethyl-2-azabicyclo[3.3.1]nonan-9-ylidene)acetonitrile* (**32**). Using the *N*-Boc **31** to obtain the secondary amine (1*R*,5*R*)-5-(3-methoxyphenyl)-2-azabicyclo[3.3.1]nonan-9-ylidene)acetonitrile on a 2.50 g scale, the procedure followed that of **26** to give rise to the secondary amine, leading to **27**. The secondary amine 2-((1*R*,5*R*)-5-(3-methoxyphenyl)-2-azabicyclo[3.3.1]nonan-9-ylidene)acetonitrile was obtained as a yellow oil and was used directly in the next step without purification. ^1^H-NMR (400 MHz; CDCl_3_): δ 7.27–7.23 (m, 1H), 6.84 (d, *J* = 7.9 Hz, 1H), 6.80 (t, *J* = 2.0 Hz, 1H), 6.76 (dd, *J* = 8.1, 2.3 Hz, 1H), 4.58 (s, 1H), 4.29 (bs, 1H), 3.76 (s, 3H), 3.45–3.38 (m, 1H), 2.93 (dt, *J* = 13.1, 6.3 Hz, 1H), 2.37–2.22 (m, 3H), 2.15–2.07 (m, 4H), 1.88–1.78 (m, 1H), 1.75–1.69 (m, 1H). ^13^C-NMR (101 MHz; CDCl_3_): δ 175.31, 159.51, 147.07, 129.36, 119.51, 116.54, 113.80, 111.26, 92.18, 55.22, 53.91, 46.09, 41.81, 41.51, 39.51, 34.61, 20.04. HRMS-ESI (*m/z*): [M+H]^+^ calcd for C_17_H_21_N_2_O: 269.1654, found: 269.1654, [α]^20^_D_ +66.0° (*c* 0.98, CHCl_3_).

Using the 1*R*,5*R* secondary amine above, the procedure for the tertiary amine **32** followed that of the preparation of **27** on a 1.89 g scale. A colorless oil (**32**, 2.32 g, 88%) was obtained. ^1^H-NMR (400 MHz; CDCl_3_): δ 7.31–7.18 (m, 6H), 6.89–6.79 (m, 3H), 4.62 (s, 1H), 4.11–4.09 (m, 1H), 3.80 (s, 3H), 3.16–3.10 (m, 1H), 2.93–2.84 (m, 4H), 2.78–2.72 (m, 1H), 2.39–2.35 (m, 1H), 2.24–2.05 (m, 5H), 1.70–1.66 (m, 1H), 1.65–1.54 (m, 1H). ^13^C-NMR (101 MHz; CDCl_3_): δ 173.79, 159.51, 147.46, 140.15, 129.35, 128.77, 128.36, 126.03, 119.68, 116.75, 113.92, 111.27, 93.40, 60.44, 58.82, 55.24, 48.28, 45.79, 39.58, 38.77, 34.49, 32.58, 19.49. HRMS-ESI (*m/z*): [M+H]^+^ calcd for C_25_H_29_N_2_O: 373.2280, found: 373.2275, [α]^20^_D_ +12.3° (*c* 1.09, CHCl_3_).

*2-((1R,5R)-5-(3-hydroxyphenyl)-2-phenethyl-2-azabicyclo[3.3.1]nonan-9-ylidene)acetonitrile* (**33**). Using **32**, the procedure followed that of the preparation of **28** on a 500 mg scale. A white foam (292 g, 61%) was obtained. Crystallization produced **33** as a white solid (76 mg, 68% recovery). ^1^H-NMR (400 MHz; CDCl_3_): δ 7.30–7.17 (m, 6H), 6.84–6.82 (m, 1H), 6.76 (t, *J* = 2.0 Hz, 1H), 6.72 (dd, *J* = 8.0, 1.9 Hz, 1H), 4.64 (s, 1H), 4.13–4.11 (m, 1H), 3.19–3.13 (m, 1H), 2.91–2.83 (m, 4H), 2.80–2.74 (m, 1H), 2.41–2.34 (m, 1H), 2.23–2.07 (m, 5H), 1.71–1.66 (m, 1H), 1.63–1.55 (m, 1H). ^13^C-NMR (101 MHz; CDCl_3_): δ 173.58, 155.79, 147.52, 139.93, 129.55, 128.74, 128.39, 126.1, 119.39, 116.69, 114.54, 113.98, 93.49, 60.29, 58.67, 48.22, 45.69, 39.54, 38.47, 34.33, 32.17, 19.64. HRMS-ESI (*m/z*): [M+H]+ calcd for C_24_H_27_N_2_O: 359.2123, found: 359.2122, mp 154 °C, [α]^20^_D_ +11.5° (*c* 1.02, CHCl_3_). CHN calc. for C_24_H_27_ClN_2_O · 1.45 H_2_O: C, 68.46%; H, 7.16%; N, 6.65%; found C, 68.19%; H, 6.87%; N, 6.44%.

*2-((1S,5R,9S)-5-(3-Hydroxyphenyl)-2-phenethyl-2-azabicyclo[3.3.1]nonan-9-yl)acetonitrile* (**34**). We dissolved the unsaturated nitrile **28** (170 mg, 0.474 mmol, 1 equiv) in EtOH (33 mL) and passed it through the H-cube flow reactor under the following conditions: 70 °C, 50 bar H_2_, 10% Pd/C, 1.2 mL/min flow rate. The reaction mixture was concentrated and purified by chromatography with 0 to 8% CMA/CHCl_3_ to give rise to a white foam (30 mg, 18%). Crystallization was performed by dissolving 30 mg of product in minimal aq MeOH, then adding 2 N HCl in Et_2_O (2 equiv) to produce **34** as a white solid (23 mg, 70% recovery). ^1^H-NMR (400 MHz; CDCl_3_): δ 7.31–7.16 (m, 6H), 6.92 (bs, 1H), 6.84 (d, *J* = 8.0 Hz, 1H), 6.75 (dd, *J* = 8.0, 2.2 Hz, 1H), 3.34 (d, *J* = 1.3 Hz, 1H), 3.19–3.17 (m, 1H), 3.17–3.01 (m, 1H), 2.99–2.89 (m, 5H), 2.41–2.39 (m, 1H), 2.39–2.38 (m, 1H), 2.13–1.61 (m, 7H), 1.55–1.49 (m, 1H). ^13^C-NMR (101 MHz; CDCl_3_): δ 156.49, 149.10, 139.63, 129.89, 128.72, 128.48, 126.25, 118.73, 117.20, 113.83, 112.86, 57.82, 54.76, 49.46, 42.09, 40.25, 37.62, 33.90, 27.93, 21.09, 18.11, 16.52. HRMS-ESI (*m/z*): [M+H]^+^ calcd for C_24_H_29_N_2_O: 361.2280, found: 361.2282, mp 63–66 °C, [α]^20^_D_ −2.43° (*c* 1.30, CHCl_3_). CHN calc. for C_24_H_29_ClN_2_O: C, 72.62%; H, 7.36%; N, 7.06%; found C, 72.69%; H, 7.24%; N, 7.11%.

*2-((1R,5S,9R)-5-(3-hydroxyphenyl)-2-phenethyl-2-azabicyclo[3.3.1]nonan-9-yl)acetonitrile* (**35**). Using **33**, the saturated cyanomethyl compound was prepared analogously to **34** on a 479 mg scale to obtain **35** as a white foam (205 mg, 43%). Hydrochloride salt formation produced the 1*R*,5*S*,9*R*-saturated nitrile **35** as a white solid (180 mg, 80% recovery). ^1^H-NMR (400 MHz; CDCl_3_): δ 7.31–7.16 (m, 6H), 6.92 (bs, 1H), 6.84 (d, *J* = 8.0 Hz, 1H), 6.75 (dd, *J* = 8.0, 2.2 Hz, 1H), 3.34 (d, *J* = 1.3 Hz, 1H), 3.19–3.17 (m, 1H), 3.17–3.01 (m, 1H), 2.99–2.89 (m, 5H), 2.41–2.39 (m, 1H), 2.39–2.38 (m, 1H), 2.13–1.61 (m, 7H), 1.55–1.49 (m, 1H). ^13^C-NMR (101 MHz; CDCl_3_): δ 156.49, 149.10, 139.63, 129.89, 128.72, 128.48, 126.25, 118.73, 117.20, 113.83, 112.86, 57.82, 54.76, 49.46, 42.09, 40.25, 37.62, 33.90, 27.93, 21.09, 18.11, 16.52. HRMS-ESI (*m/z*): [M+H]^+^ calcd for C_24_H_29_N_2_O: 361.2280, found: 361.2280, mp 63–66 °C, [α]^20^_D_ +3.75° (*c* 0.99, CHCl_3_). CHN calc. for C_24_H_29_ClN_2_O · 0.1 H_2_O: C, 72.14%; H, 7.20%; N, 6.99%; found C, 72.29%; H, 7.38%; N, 7.03%.

*tert-Butyl (1S,5R,9R)-9-(cyanomethyl)-5-(3-methoxyphenyl)-2-azabicyclo[3.3.1]nonane-2-carboxylate* (**36**). The procedure followed that of the preparation of **34** on a 475 mg scale to obtain **36** as a white solid (453 mg, 95%). ^1^H-NMR (400 MHz; CDCl_3_): δ 7.29–7.24 (m, 1H), 6.84 (t, *J* = 6.7 Hz, 1H), 6.80–6.74 (m, 2H), 4.56 (d, *J* = 2.6 Hz, 1H), 4.15–4.09 (m, 1H), 3.79 (s, 3H), 3.58–3.49 (m, 1H), 2.43 (d, *J* = 11.7 Hz, 1H), 2.28–2.21 (m, 1H), 2.11–1.69 (m, 9H), 1.47 (s, 9H). ^13^C-NMR (101 MHz; CDCl_3_): δ 159.93, 155.77, 148.94, 129.87, 119.19, 117.14, 111.92, 110.79, 80.21, 55.19, 48.96, 42.46, 41.76, 40.54, 38.22, 31.17, 28.42, 27.34, 21.69, 16.64. HRMS-ESI (*m/z*): [M+Na]^+^ calcd for C_22_H_30_N_2_O_3_Na: 393.2154, found: 393.2152, mp 143–146 °C, [α]^20^_D_ −63.8° (*c* 1.09, CHCl_3_).

*2-((1S,5R,9R)-5-(3-Methoxyphenyl)-2-phenethyl-2-azabicyclo[3.3.1]nonan-9-yl)acetonitrile* (**37**). We combined **36** (453 mg, 1.22 mmol, 1 equiv), dichloromethane (4.5 mL), and TFA (1.88 mL, 24.5 mmol, 20 equiv) in a flask and stirred them at room temperature. After 30 min, TLC showed no starting material. The reaction was quenched with NaHCO_3_; the aqueous layer was extracted with dichloromethane 3 times; and the combined organic layers were dried over Na_2_SO_4_. The drying agent was filtered off, and the filtrate was concentrated to obtain the secondary amine, 2-((1*S*,5*R*,9*R*)-5-(3-methoxyphenyl)-2-azabicyclo[3.3.1]nonan-9-yl)acetonitrile, as a golden oil, which was used directly in the next step without purification. ^1^H-NMR (400 MHz; CDCl_3_): δ 7.24 (t, *J* = 8.0 Hz, 1H), 6.86 (dd, *J* = 7.6, 1.3 Hz, 1H), 6.79 (t, *J* = 2.1 Hz, 1H), 6.72 (dd, *J* = 8.1, 2.4 Hz, 1H), 3.78 (s, 3H), 3.63 (td, *J* = 12.5, 5.5 Hz, 1H), 3.32 (d, *J* = 3.1 Hz, 1H), 3.04 (dd, *J* = 12.4, 7.6 Hz, 1H), 2.76 (dd, *J* = 16.7, 11.6 Hz, 1H), 2.35 (d, *J* = 11.5 Hz, 1H), 2.17–1.72 (m, 10H). ^13^C-NMR (101 MHz; CDCl_3_): δ 159.82, 150.24, 129.66, 120.12, 117.28, 111.89, 110.55, 55.18, 48.97, 42.46, 41.98, 41.67, 38.53, 34.04, 28.85, 22.95, 16.72. HRMS-ESI (*m/z*): [M+H]^+^ calcd for C_17_H_23_N_2_O: 271.1810, found: 271.1806, [α]^20^_D_ −8.6° (*c* 1.03, CHCl_3_).

The conversion of the secondary amine to the tertiary amine followed the preparation of **27** on a 331 mg scale to obtain **37** as a yellow oil (345 mg, 75%). ^1^H-NMR (400 MHz; CDCl_3_): δ 7.29–7.15 (m, 6H), 6.87–6.85 (m, 1H), 6.80 (s, 1H), 6.74 (d, *J* = 8.0 Hz, 1H), 3.79 (s, 3H), 3.15 (s, 1H), 3.09–2.96 (m, 2H), 2.86–2.72 (m, 5H), 2.40 (d, *J* = 10.8 Hz, 1H), 2.34–2.30 (m, 1H), 2.08–1.66 (m, 7H), 1.56–1.49 (m, 1H). ^13^C-NMR (101 MHz; CDCl_3_): δ 159.83, 150.13, 140.56, 129.66, 128.67, 128.29, 125.91, 120.48, 117.44, 112.00, 110.56, 56.48, 55.19, 54.13, 48.25, 43.85, 42.29, 38.45, 34.35, 29.17, 25.57, 23.06, 16.37. HRMS-ESI (*m/z*): [M+H]^+^ calcd for C_25_H_31_N_2_O: 375.2436, found: 375.2437 [α]^20^_D_ −9.5° (*c* 1.05, CHCl_3_).

*2-((1S,5R,9R)-5-(3-Hydroxyphenyl)-2-phenethyl-2-azabicyclo[3.3.1]nonan-9-yl)acetonitrile* (**38**). Using **37**, the procedure followed that of the preparation of **8** on a 345 mg scale to obtain **38** as a white foam (156 mg, 47%). Crystallization was performed by dissolving 100 mg of product in minimal aqueous MeOH and then adding 2 N HCl in Et_2_O (2 equiv) to produce a white solid (82 mg, 74% recovery). ^1^H-NMR (400 MHz; CDCl_3_): δ 7.32–7.19 (m, 6H), 6.88 (d, *J* = 7.9 Hz, 1H), 6.82 (s, 1H), 6.71 (dd, *J* = 7.9, 2.2 Hz, 1H), 3.31 (s, 1H), 3.08–3.04 (m, 2H), 2.94–2.82 (m, 4H), 2.73–2.69 (m, 1H), 2.29–2.22 (m, 2H), 2.14–1.77 (m, 7H), 1.50–1.49 (m, 1H). ^13^C-NMR (101 MHz; CDCl_3_): δ 156.83, 149.29, 139.88, 130.03, 128.74, 128.50, 126.25, 118.95, 117.03, 114.09, 113.39, 57.92, 54.59, 49.41, 42.27, 40.43, 37.68, 33.99, 27.99, 21.29, 18.11, 16.58.HRMS-ESI (*m/z*): [M+H]^+^ calcd for C_24_H_29_N_2_O: 361.2280, found: 361.2282, mp 73–76 °C, [α]^20^_D_ +14.0° (*c* 1.01, CHCl_3_). CHN calc. for C_24_H_29_ClN_2_O · 0.5 H_2_O: C, 71.01%; H, 7.45%; N, 6.90%; found C, 71.10%; H, 7.33%; N, 6.76%.

*tert-Butyl (1R,5S,9S)-9-(cyanomethyl)-5-(3-methoxyphenyl)-2-azabicyclo[3.3.1]nonane-2-carboxylate* (**39**). The procedure followed that of the preparation of **34** on a 155 mg scale to obtain **39** as a white foam (156 mg, 99%). ^1^H-NMR (400 MHz; CDCl_3_): δ 7.28–7.24 (m, 1H), 6.84 (t, *J* = 6.6 Hz, 1H), 6.79–6.73 (m, 2H), 4.56 (d, *J* = 2.5 Hz, 1H), 4.14–4.09 (m, 1H), 3.78 (s, 3H), 3.57–3.49 (m, 1H), 2.43 (d, *J* = 11.6 Hz, 1H), 2.27–2.20 (m, 1H), 2.19–1.69 (m, 9H), 1.47 (s, 9H). ^13^C-NMR (101 MHz; CDCl_3_): δ 159.93, 155.78, 148.95, 129.87, 119.20, 117.14, 111.92, 110.79, 80.22, 55.19, 48.97, 42.47, 41.77, 40.55, 38.23, 31.17, 28.42, 27.34, 21.69, 16.65. HRMS-ESI (*m/z*): [M+Na]^+^ calcd for C_22_H_30_N_2_O_3_Na: 393.2154, found: 393.2151, mp 144–146 °C, [α]^20^_D_ +64.6° (*c* 0.99, CHCl_3_).

*2-((1R,5S,9S)-5-(3-Methoxyphenyl)-2-phenethyl-2-azabicyclo[3.3.1]nonan-9-yl)acetonitrile* (**40**). The procedure to obtain the intermediate secondary amine, 2-((1*R*,5*S*,9*S*)-5-(3-methoxyphenyl)-2-azabicyclo[3.3.1]nonan-9-yl)acetonitrile, followed that of the preparation of **37** on a 156 mg scale to obtain a golden oil, which was used directly in the next step without purification. ^1^H-NMR (400 MHz; CDCl_3_): δ 7.24 (t, *J* = 8.0 Hz, 1H), 6.86 (dd, *J* = 7.6, 1.3 Hz, 1H), 6.79 (t, *J* = 2.1 Hz, 1H), 6.72 (dd, *J* = 8.1, 2.4 Hz, 1H), 3.78 (s, 3H), 3.63 (td, *J* = 12.5, 5.5 Hz, 1H), 3.32 (d, *J* = 3.1 Hz, 1H), 3.04 (dd, *J* = 12.4, 7.6 Hz, 1H), 2.76 (dd, *J* = 16.7, 11.6 Hz, 1H), 2.35 (d, *J* = 11.5 Hz, 1H), 2.17–1.72 (m, 10H). ^13^C-NMR (101 MHz; CDCl_3_): δ 159.82, 150.24, 129.66, 120.12, 117.28, 111.89, 110.55, 55.18, 48.97, 42.46, 41.98, 41.67, 38.53, 34.04, 28.85, 22.95, 16.72. HRMS-ESI (*m/z*): [M+H]^+^ calcd for C_17_H_23_N_2_O: 271.1810, found: 271.1810 [α]^20^_D_ +9.81° (*c* 1.03, CHCl_3_).

The procedure to convert the secondary amine to the tertiary amine **40** followed that of the preparation of **37** on a 114 mg scale to obtain the tertiary amine **40** as a yellow oil (116 mg, 73%). ^1^H-NMR (400 MHz; CDCl_3_): δ 7.32–7.18 (m, 6H), 6.88 (d, *J* = 7.9 Hz, 1H), 6.83 (s, 1H), 6.76 (dd, *J* = 8.1, 2.1 Hz, 1H), 3.81 (s, 3H), 3.18 (s, 1H), 3.13–2.99 (m, 2H), 2.89–2.74 (m, 5H), 2.43 (m, *J* = 11.1 Hz, 1H), 2.34 (dd, *J* = 14.0, 3.4 Hz, 1H), 2.10–1.69 (m, 7H), 1.59–1.52 (m, 1H). ^13^C-NMR (101 MHz; CDCl_3_): δ 159.84, 150.13, 140.56, 129.67, 128.68, 128.29, 125.92, 120.47, 117.44, 112.00, 110.58, 56.47, 55.20, 54.13, 48.26, 43.85, 42.28, 38.45, 34.35, 29.17, 25.56, 23.06, 16.38. HRMS-ESI (*m/z*): [M+H]^+^ calcd for C_25_H_31_N_2_O: 375.2436, found: 375.2436, [α]^20^_D_ +8.8° (*c* 1.07, CHCl_3_).

*2-((1R,5S,9S)-5-(3-Hydroxyphenyl)-2-phenethyl-2-azabicyclo[3.3.1]nonan-9-yl)acetonitrile* (**41**). Using **40**, the procedure followed that of the preparation of **8** on a 116 mg scale to obtain the phenol **41** as a white foam (53 mg, 77%). Crystallization was performed by dissolving 63 mg of product in minimal aqueous MeOH and then adding 2 N HCl in Et_2_O (2 equiv) to afford a white solid (65 mg, 63% recovery). ^1^H-NMR (400 MHz; CDCl_3_): δ 7.32–7.19 (m, 6H), 6.88 (d, *J* = 7.9 Hz, 1H), 6.82 (s, 1H), 6.71 (dd, *J* = 7.9, 2.2 Hz, 1H), 3.31 (s, 1H), 3.08–3.04 (m, 2H), 2.94–2.82 (m, 4H), 2.73–2.69 (m1H), 2.29–2.22 (m, 2H), 2.14–1.77 (m, 7H), 1.50–1.49 (m, 1H). ^13^C-NMR (101 MHz; CDCl_3_): δ 156.83, 149.29, 139.88, 130.03, 128.74, 128.50, 126.25, 118.95, 117.03, 114.09, 113.39, 57.92, 54.59, 49.41, 42.27, 40.43, 37.68, 33.99, 27.99, 21.29, 18.11, 16.58. HRMS-ESI (*m/z*): [M+H]^+^ calcd for C_24_H_29_N_2_O: 361.2280, found: 361.2283, mp 73–76 °C, [α]^20^_D_ −10.5° (*c* 0.93, CHCl_3_). CHN calc. for C_24_H_29_ClN_2_O · 0.35 H_2_O: C, 71.48%; H, 7.42%; N, 6.95%; found C, 71.49%; H, 7.28%; N, 6.83%.

1*-(1S,5R,9S)-5-(3-Methoxyphenyl)-2-phenethyl-2-azabicyclo[3.3.1]nonan-9-yl)-N-methylmethanamine* (**44**) *and 1-(1S,5R,9R)-5-(3-methoxyphenyl)-2-phenethyl-2-azabicyclo[3.3.1]nonan-9-yl)-N-methylmethanamine* (**45**). Enol ether **42** (1.00 g, 1 equiv, 2.65 mmol) was dissolved in tetrahydrofuran (7.5 mL) and 6 M HCl (10 mL). The reaction was stirred under argon at room temperature overnight. The reaction was cooled down to 0 °C and basified with 7 N NH_4_OH in methanol (pH 8.5). The aqueous solution was then extracted with CHCl_3_ (5 × 20 mL). The organic layers were combined and washed with water (20 mL) and brine (20 mL), dried over anhydrous Na_2_SO_4_, and filtered. The solvent was removed under vacuum conditions to give rise to the mixture of aldehydes (**43**) as a bluish green oil. The aldehydes were dried under high vacuum for 30 min and used immediately in the next step without purification. The mixture of aldehydes was dissolved in ethanol (10 mL) and transferred to a pressure flask. Titanium(IV) isopropoxide (1.51 g, 1.61 mL, 2 equiv, 5.30 mmol) and 33% methylamine solution in ethanol (2.49 g, 3.30 mL, 10 equiv, 26.5 mmol) were added to the flask. The pressure flask was flushed with argon, sealed, and stirred at 50 °C overnight. The reaction was cooled to 0 °C. Sodium borohydride (200 mg, 2 equiv, 5.30 mmol) was then added portionwise, and the reaction was stirred for 2 h. Water was added to quench the reaction, and the pH was adjusted to 9 by adding NH_4_OH solution. The reaction mixture was filtered through celite and washed several times with ethyl acetate. The filtrate was transferred to a separatory funnel. The aqueous phase was extracted with ethyl acetate (3 × 20 mL). The organic layer was washed with brine (20 mL), dried over anhydrous Na_2_SO_4_, and filtered. The solvent was removed under vacuum conditions to yield a dark-yellow residue. The residue was separated and purified by flash chromatography (slow gradient 0–20% CHCl_3_/MeOH/NH_4_OH) to give rise to the diastereomers *1S,5R,9S*-**44** and *1S,5R,9R*-**45** in a 75% yield for the combined diastereomers.

For 1*S*,*5R*,9*S*-**44**, the product was isolated as a pale-yellow oil (437.5 mg). ^1^H NMR (400 MHz; CDCl_3_) δ: 7.27–7.24 (m, 3H), 7.20 (dd, *J* = 7.1, 1.9 Hz, 2H), 7.15 (t, *J* = 7.1 Hz, 1H), 6.92 (d, *J* = 7.9 Hz, 1H), 6.86 (t, *J* = 1.9 Hz, 1H), 6.70 (dd, *J* = 8.1, 2.3 Hz, 1H), 3.79 (s, 3H), 3.05 (dd, *J* = 11.4, 4.8 Hz, 2H), 2.98 (d, *J* = 2.8 Hz, 1H), 2.85–2.66 (m, 5H), 2.30–2.25 (m, 2H), 2.10 (s, 3H), 2.08 (d, *J* = 3.8 Hz, 1H), 1.95 (dt, *J* = 11.6, 4.4 Hz, 2H), 1.87 (dt, *J* = 12.5, 6.1 Hz, 1H), 1.82–1.77 (m, 1H), 1.70–1.62 (m, 3H), 1.49–1.42 (m, 1H). ^13^C NMR (101 MHz; CDCl_3_): δ 159.46, 151.76, 141.96, 129.02, 128.67, 128.10, 125.76, 117.86, 112.03, 109.94, 56.69, 55.11, 52.96, 49.37, 48.81, 45.83, 42.86, 38.21, 36.87, 34.04, 30.14, 25.81, 23.31. HRMS-ESI (m/z): [M+H]^+^ calc for C_25_H_35_N_2_O: 379.2749; found, 379.2747.

For 1*S*,5*R*,9*R*-**45**, the product was obtained as a pale-yellow oil (314.5 mg). ^1^H NMR (400 MHz; CDCl_3_) δ: 7.30–7.17 (m, 6H), 7.01 (d, *J* = 8.0 Hz, 1H), 6.95 (s, 1H), 6.72 (dd, *J* = 8.1, 2.2 Hz, 1H), 3.79 (s, 3H), 3.20 (bs, 1H), 3.01 (dd, *J* = 10.1, 3.8 Hz, 2H), 2.88–2.77 (m, 4H), 2.49 (t, *J* = 11.1 Hz, 1H), 2.39 (d, *J* = 10.6 Hz, 1H), 2.26 (s, 3H), 2.24–2.20 (m, 2H), 2.01–1.91 (m, 4H), 1.81–1.71 (m, 2H), 1.55–1.49 (m, 2H). ^13^C NMR (101 MHz; CDCl_3_): δ 159.51, 151.56, 140.69, 129.09, 128.73, 128.31, 125.91, 117.99, 112.08, 110.31, 58.26, 55.15, 53.57, 50.00, 49.76, 45.48, 41.57, 37.84, 36.70, 34.65, 29.32, 21.72, 18.58. HRMS-ESI (m/z): [M+H]^+^ calc for C_25_H_35_N_2_O: 379.2749; found, 379.2747.

*1-((1S,5R,9S)-5-(3-Methoxyphenyl)-2-phenethyl-2-azabicyclo[3.3.1]nonan-9-yl)-N-methylmethanamine* (**46**). The ether **44** (320 mg, 1 equiv, 845 µmol) was dissolved in anhydrous dichloromethane (20 mL), and the solution was cooled to −78 °C. To this cooled solution was added boron tribromide (1.06 mg, 400 µL, 5 equiv, 4.23 mmol) dropwise. The reaction was stirred at −78 °C for 15 min and then warmed up to room temperature for 2 h. The reaction was cooled to 0 °C, quenched with MeOH, and stirred for 30 min. Then, 2 N HCl (10 mL) was added and distilled at 100 °C (to remove the volatile solvents) under nitrogen conditions for 1 h. The reaction was allowed to cool to room temperature and basified to pH 10 using aqueous NH_4_OH. The aqueous phase was extracted with CHCl_3_:MeOH (9:1) (3 × 20 mL). The organic layers were combined and washed with brine (20 mL). The organic layer was dried over anhydrous Na_2_SO_4_ and filtered. The solvent was removed in vacuo to give rise to a pale-yellow oil. The crude sample was purified using flash chromatography (0–20% CHCl_3_/MeOH/NH_4_OH), and **46** was obtained as a pale-yellow oil (200 mg, 65%). ^1^H NMR (400 MHz; CDCl_3_): δ 7.27–7.10 (m, 6H), 6.78–6.76 (m, 2H), 6.58 (dd, *J* = 8.1, 1.5 Hz, 1H), 3.47 (bs, 1H), 3.10–3.02 (m, 2H), 2.93 (s, 1H), 2.84–2.73 (m, 4H), 2.67 (dd, *J* = 11.7, 9.0 Hz, 1H), 2.27–2.20 (m, 3H), 2.13 (dd, *J* = 11.8, 4.0 Hz, 1H), 2.06 (s, 3H), 1.97–1.88 (m, 1H), 1.87–1.85 (m, 2H), 1.84–1.72 (m, 1H), 1.70–1.61 (m, 1H), 1.45–1.38 (m, 1H). ^13^C NMR (101 MHz; CDCl_3_): δ 157.32, 151.11, 140.82, 129.26, 128.63, 128.17, 125.85, 116.32, 113.51, 112.78, 56.58, 53.58, 49.70, 48.74, 45.04, 42.86, 38.07, 36.30, 33.92, 29.98, 25.73, 23.28. HRMS-ESI (*m/z*): [M+H]^+^ calc for C_24_H_33_N_2_O: 365.2593; found, 365.2593. [α]^20^_D_ +8.8 (*c* 1.1, CHCl_3_), mp (dioxalate salt): 143.4–145.1 °C. CHN calcd for C_28_H_36_N_2_O_9_ • 0.7H_2_O: C, 60.36; H, 6.77; N, 5.03. Found: C, 60.29; H, 6.72; N, 5.12.

*3-((1S,5R,9R)-9-((Methylamino)methyl)-2-phenethyl-2-azabicyclo[3.3.1]nonan-5-yl)phenol* (**47**). The ether **45** (180 mg, 1 equiv, 475 µmol) was dissolved in anhydrous dichloromethane (15 mL), and the solution was cooled to −78 °C. To this cooled solution was added boron tribromide (596 mg, 225 µL, 5 equiv, 2.38 mmol) dropwise. The reaction was stirred at −78 °C for 15 min and then warmed up to room temperature for 2 h. The reaction was cooled to 0 °C, quenched with MeOH, and stirred for 30 min. Then, 2 N HCl (10 mL) was added and distilled at 100 °C (to remove the volatile solvents) under nitrogen conditions for 1 h. The reaction was allowed to cool to room temperature and basified to pH 10 using aqueous NH_4_OH. The aqueous phase was extracted with CHCl_3_:MeOH (9:1) (3 × 20 mL). The organic layers were combined and washed with brine (20 mL). The organic layer was dried over anhydrous Na_2_SO_4_ and filtered. The solvent was removed in vacuo to give rise to a pale-yellow foam. The crude sample was purified using flash chromatography (0–20% CHCl_3_/MeOH/NH_4_OH), and **47** was obtained as an off-white foam (140 mg, 81%). ^1^H NMR (400 MHz; CD_3_OD): δ 7.28–7.26 (m, 1H), 7.24–7.22 (m, 3H), 7.18–7.14 (m, 1H), 7.14–7.10 (m, 1H), 6.88 (d, *J* = 8.0 Hz, 1H), 6.84 (t, *J* = 2.0 Hz, 1H), 6.60 (dd, *J* = 7.9, 2.2 Hz, 1H), 3.29 (s, 1H), 3.06 (td, *J* = 12.0, 5.0 Hz, 1H), 2.99–2.95 (m, 1H), 2.90–2.78 (m, 4H), 2.58 (dd, *J* = 12.1, 10.6 Hz, 1H), 2.43 (dd, *J* = 10.4, 2.7 Hz, 1H), 2.25–2.17 (m, 5H), 2.04–1.95 (m, 4), 1.84–1.79 (m, 1H), 1.72 (ddd, *J* = 14.1, 4.9, 2.0 Hz, 1H), 1.59–1.56 (m, 1H). ^13^C NMR (101 MHz; CDCl_3_): δ 157.65, 150.96, 140.34, 129.28, 128.71, 128.34, 125.99, 116.40, 114.01, 113.08, 58.26, 55.12, 50.67, 49.39, 44.88, 40.54, 37.51, 36.08, 34.14, 29.14, 21.39, 19.08. HRMS-ESI (*m/z*): [M+H]^+^ calc for C_24_H_33_N_2_O: 365.2593; found, 365.2594. [α]^20^_D_ −30.3 (*c* 1.0, CHCl_3_). mp: 134.2–135.3 °C. CHN calc for C_24_H_32_N_2_O • 0.03 H_2_O • 0.24 CHCl_3_: C, 73.95; H, 8.27; N, 7.12. Found: C, 74.09; H, 8.36; N, 6.95.

*(1R,5R)-9-(Methoxymethylene)-5-(3-methoxyphenyl)-2-phenethyl-2-azabicyclo[3.3.1]nonane* (**48**) [5]. 1*R*,5*R*-**4** (2.20 g, 1 equiv, 2.86 mmol) was transferred to an oven-dried flask charged with (methoxymethyl)triphenylphosphonium chloride (6.47 g, 3 equiv, 8.58 mmol). The solution was cooled down to 0 °C, to which was added lithium bis(trimethylsilyl)amide (4.21 g, 25.2 mL, 1 molar, 4 equiv, 11.4 mmol) dropwise. The reaction was gradually allowed to warm to room temperature and stirred overnight. The reaction was then cooled down to 0 °C, quenched with MeOH, and stirred for 10 min. The reaction was concentrated under vacuum conditions, and the residue was taken up in water and chloroform. The pH of the aqueous layer was adjusted to 9 with a saturated aqueous NH_4_OH solution. The aqueous phase was extracted with 9:1 CHCl_3_/MeOH (3 × 25 mL), and the combined organic layer was dried over anhydrous Na_2_SO_4_, filtered, and concentrated under vacuum conditions. The residue was purified using flash chromatography (gradient 0–100% EtOAc in hexanes) to give rise to **48** as a mixture of E/Z isomers (1.70 g, 71%) that were immediately used in the next step.

*1-(1R,5S,9R)-5-(3-Methoxyphenyl)-2-phenethyl-2-azabicyclo[3.3.1]nonan-9-yl)-N-methylmethanamine* (**50**) *and 1-((1R,5S,9S)-5-(3-methoxyphenyl)-2-phenethyl-2-azabicyclo[3.3.1]nonan-9-yl)-N-methylmethanamin*e (**51**). The enol ether **48** (0.700 g, 1 equiv, 1.85 mmol) was dissolved in tetrahydrofuran (5 mL), and 6 N HCl (15 mL) was added to it. The reaction was stirred under nitrogen conditions at room temperature overnight. The reaction was cooled down to 0 °C and basified with aqueous NH_4_OH. The aqueous solution was then extracted with CHCl_3_/MeOH (9:1) (5 × 10 mL). The organic layers were combined and washed with water and brine, dried over anhydrous MgSO_4_, filtered, and concentrated under vacuum conditions to give rise to a mixture of aldehydes as a bluish green oil. The aldehydes **49** were immediately used in the next step without purification. The mixture of aldehydes, methylamine (2.31 mL, 33% in EtOH, 10 equiv, 18.5 mmol), and titanium(IV) isopropoxide (1.05 g, 1.12 mL, 2 equiv, 3.71 mmol) was added to a clean, dry-pressure vial. The reaction was stirred under argon at 60 °C overnight. The reaction was cooled to 0 °C, and sodium borohydride (140 mg, 2 equiv, 3.71 mmol) was added. The reaction was stirred for 2 h, quenched with water, and basified to pH 9 with an NH_4_OH solution. The solids were filtered off over celite, and the filtrate was extracted with EtOAc (3 × 20 mL). The organic layers were combined, washed with brine, dried over anhydrous MgSO_4_, and filtered, and the solvent was removed in vacuo to give rise to a yellow residue, a mixture of diastereomers that were separated using flash chromatography (0–5% CHCl_3_/MeOH/NH_4_OH) to give **50** (1R,5S,9R) and **51** (1R,5S,9S) as pale-yellow oils (0.301 g, 43%). ^1^H NMR (400 MHz; CDCl_3_): δ 7.28–7.25 (m, 3H), 7.22 (d, J = 8 Hz, 2H), 7.17 (t, J_1_ = 4 Hz, J_2_ = 8 Hz, 1H), 6.93 (d, J = 8Hz, 1H), 6.87 (s, 1H), 6.71 (d, J = 8 Hz, 1H), 3.80 (s, 3H), 3.10–3.05 (m, 2H), 2.99 (s, 1H), 2.85–2.68 (m, 5H), 2.31–2.27 (m, 2H), 2.12 (s, 3H), 2.11–2.08 (m, 1H), 1.98–1.94 (m, 2H), 1.92–1.79 (m, 2H), 1.82–1.64 (m, 3H), 1.49–1.41 (m, 1H). ^13^C NMR (101 MHz; CDCl_3_): δ 159.46, 151.78, 140.97, 129.02, 128.67, 128.10, 125.76, 117.87, 112.03, 109.93, 56.69, 55.11, 52.95, 49.38, 48.82, 45.87, 42.87, 38.21, 36.90, 34.05, 30.15, 25.82, 23.32. HRMS-ESI (*m/z*): [M+H]^+^ calc for C_25_H_35_N_2_O: 379.2749; found, 379.2756.

For 1*R*,5*S*,9*S*-**51**, the product was obtained as a pale-yellow oil. ^1^H NMR (400 MHz; CDCl_3_) δ: 7.30–7.17 (m, 6H), 7.01 (d, *J* = 8.0 Hz, 1H), 6.95 (s, 1H), 6.72 (dd, *J* = 8.1, 2.2 Hz, 1H), 3.79 (s, 3H), 3.20 (bs, 1H), 3.03–2.99 (m, 2H), 2.88–2.77 (m, 4H), 2.49 (t, *J* = 11.1 Hz, 1H), 2.39–2.35 (m, 1H), 2.27 (s, 3H), 2.24–2.18 (m, 2H), 2.02–1.87 (m, 4H), 1.81–1.71 (m, 3H), 1.55–1.48 (m, 1H). ^13^C NMR (101 MHz; CDCl_3_): δ 159.50, 151.61, 140.73, 129.08, 128.73, 128.30, 125.90, 117.99, 112.07, 110.29, 58.29, 55.15, 53.54, 50.03, 49.78, 45.45, 41.62, 37.86, 36.76, 34.70, 29.33, 21.74, 18.59.

*3-((1R,5S,9R)-9-((Methylamino)methyl)-2-phenethyl-2-azabicyclo[3.3.1]nonan-5-yl)phenol* (**52**). The ether **50** (0.130 g, 1 equiv, 343 µmol) was dissolved in anhydrous dichloromethane (5 mL), and the solution was cooled to −78 °C. To this cooled solution was added boron tribromide (430 mg, 162 µL, 5 equiv, 1.72 mmol) dropwise. The reaction was stirred at −78 °C for 15 min and then warmed up to room temperature for 2 h. The reaction was cooled down to 0 °C, quenched with MeOH, and stirred for 30 min. Then, 1 N HCl (10 mL) was added and distilled at 100 °C (to remove the volatile solvents) under nitrogen conditions for 1 h. The reaction was allowed to cool down and basified to pH 8.5. The aqueous phase was extracted with CHCl_3_ (3 × 10 mL). The organic layers were combined and washed with brine (20 mL). The organic layer was dried over anhydrous MgSO_4_ and filtered, and the solvent was removed in vacuo to give rise to **52** as a pale-yellow oil (120 mg, 96%). ^1^H NMR (400 MHz; CDCl_3_): δ 7.28–7.10 (m, 6H), 6.77–6.76 (m, 2H), 6.58 (d, J = 8 Hz, 1H), 4.62 (bs, 1H), 3.07–3.02 (m, 2H), 2.93 (s, 1H), 2.84–2.73 (m, 4H), 2.69–2.64 (m, 1H), 2.31–2.18 (m, 3H), 2.14 (dd, J = 8 Hz, 1H), 2.06 (s, 3H), 1.96–1.79 (m, 3H), 1.72–1.68 (m, 1H), 1.64–1.60 (m, 1H), 1.43–1.39 (m, 1H). ^13^C NMR (101 MHz; CDCl_3_): δ 157.48, 151.03, 140.82, 129.26, 128.63, 128.18, 125.86, 116.20, 113.58, 112.85, 56.58, 53.59, 49.64, 48.73, 44.85, 42.85, 38.04, 36.19, 33.89, 29.95, 25.68, 23.26. HRMS-ESI (*m/z*): [M+H]^+^ calc for C_24_H_33_N_2_O: 365.2593; found, 365.2591. [α]^20^_D_ −7.7° (*c* 1.1, CHCl_3_). Dioxalate salt: mp 150–152 °C. CHN calcd for C_28_H_36_N_2_O_9_: C, 61.75; H, 6.66; N, 5.14. Found: C, 61.98; H, 6.93; N, 5.29.

*3-((1R,5S,9S)-9-((Methylamino)methyl)-2-phenethyl-2-azabicyclo[3.3.1]nonan-5-yl)phenol* (**53**). The ether **51** (630 mg, 1 equiv, 1.66 mmol) was dissolved in anhydrous dichloromethane (25 mL), and the solution was cooled to −78 °C. To this cooled solution was added boron tribromide (2.08 g, 787 µL, 5 equiv, 8.32 mmol) dropwise. The reaction was stirred at −78 °C for 15 min and then warmed up to room temperature for 2 h. The reaction was cooled down to 0 °C, quenched with MeOH, and stirred for 30 min. Then, 1 N HCl (20 mL) was added and distilled at 100 °C (to remove the volatile solvents) under nitrogen conditions for 1 h. The reaction was allowed to cool down and basified to pH 8.5. The aqueous phase was extracted with CHCl_3_ (3 × 20 mL). The organic layers were combined and washed with brine (20 mL). The organic layer was dried over anhydrous MgSO_4_ and filtered, and the solvent was removed in vacuo to give rise to **53** as a white foam (400 mg, 66%). ^1^H NMR (400 MHz; CD_3_OD): δ 7.28–7.22 (m, 4H), 7.18–7.14 (m, 1H), 7.12 (t, *J* = 8.0 Hz, 1H), 6.88 (d, *J* = 8.0 Hz, 1H), 6.84 (d, *J* = 1.9 Hz, 1H), 6.59 (dd, *J* = 7.9, 1.9 Hz, 1H), 3.20 (s, 1H), 3.05 (td, *J* = 12.1, 5.0 Hz, 1H), 2.99–2.94 (m, 1H), 2.89–2.77 (m, 4H), 2.56 (t, *J* = 11.3 Hz, 1H), 2.43 (d, *J* = 10.4 Hz, 1H), 2.24–2.16 (m, 5H), 2.03–1.95 (m, 4H), 1.83–1.79 (m, 1H), 1.71 (dt, *J* = 11.7, 2.4 Hz, 1H), 1.62–1.55 (m, 1H). ^13^C NMR (101 MHz; CDCl_3_): δ 157.57, 150.99, 140.36, 129.28, 128.71, 128.34, 125.99, 116.46, 113.99, 113.04, 58.27, 55.15, 50.71, 49.39, 44.92, 40.54, 37.52, 36.12, 34.17, 29.14, 21.39, 19.10. HRMS-ESI (*m/z*): [M+H]^+^ calc for C_24_H_33_N_2_O: 365.2593; found, 365.2595. [α]^20^_D_ +31.9° (*c* 1.0, CHCl_3_). Mp: 138.1–139.0 °C. CHN calcd for C_24_H_32_N_2_O • 0.05 H_2_O • 0.35 CHCl_3_: C, 71.82; H, 8.03; N, 6.88. Found: C, 71.71; H, 7.93; N, 7.06.

### 3.3. In Vitro Assay

#### 3.3.1. Cell Lines and Cell Culture

Cell lines and cell culture: cAMP Hunter^TM^ Chinese hamster ovary cells (CHO-K1) that express the human μ-opioid receptor (OPRM1), human κ-opioid receptor (OPRMK1), and human δ-receptor (OPRMD1) were purchased from Eurofins DiscoverX (Fremont, CA, USA) and used for the forskolin-induced cAMP accumulation assays [17]. All cell lines were maintained in F-12 media with 10% fetal bovine serum (Life Technologies, Grand Island, NY, USA), 1% penicillin/streptomycin/l-glutamine (Life Technologies), and 800 µg/mL of Geneticin (Mirus Bio, Madison, WI, USA). All cells were grown at 37 °C and 5% CO_2_ in a humidified incubator.

#### 3.3.2. Forskolin-Induced cAMP Accumulation Assays

Assays were performed as previously described [4]. Briefly, 10,000 cells/well of cells were plated in 384-well tissue culture plates and incubated overnight at 37 °C in 5% CO_2_. Stock solutions of the compound were prepared in DMSO at a 5 mM concentration, and then 9 to 10 concentrations of 100X working solutions were prepared by serial dilution using DMSO. Furthermore, 5X working solutions were subsequently prepared using forskolin-containing assay buffer (consisting of HBSS and HEPES). For the agonist assay, cells were incubated at 37 °C with compounds for 30 min at a 1X final concentration. For the antagonist assay [17], cells were incubated at 37 °C with compounds for 15 min before 30 min of incubation at 37 °C with the selected agonist at their EC_50_ or EC_90_ doses. Detection was carried out by using the HitHunter cAMP Assay for Small Molecules by DiscoverX according to the manufacturer’s directions, and the BioTek Synergy H1 hybrid plate reader (BioTek, Winooski, VT, USA) and Gen5 Software version 2.01 were used to quantify luminescence (BioTek, Winooski, VT, USA). To determine the % efficacy in forskolin-induced cAMP assays, background readouts of the vehicle control were subtracted from all treatment readouts and then normalized to the forskolin control. Data were then analyzed in GraphPad Prism 8 (GraphPad, LaJolla, CA, USA) using nonlinear regression. Values are expressed as the mean ± SEM of at least three independent experiments. The degree of antagonism (I_max_) was normalized to naltrexone (MOR, DOR) or *nor*-BNI (KOR).

#### 3.3.3. X-ray Crystal Data

Single-crystal X-ray diffraction data on compound **23** were collected using Cu Kα radiation and a Bruker SMART APEX II CCD area detector. The crystal was prepared for data collection by coating it with high-viscosity microscope oil. The oil-coated crystal was mounted on a micromesh mount (MiTeGen, Inc., Ithaca NY, USA) and transferred to the diffractometer, where a dataset was collected at 100(2) K. The 0.341 × 0.046 × 0.030 mm^3^ crystal was orthorhombic in space group P2_1_2_1_2_1_. The structure was solved by direct methods and refined by full-matrix least-squares refinement on F^2^ values using the programs found in the SHELXL suite (Bruker, SHELXL v2014.7, 2014, Bruker AXS Inc., Madison, WI, USA). Corrections were applied for Lorentz, polarization, and absorption effects. The parameters refined included atomic coordinates and anisotropic thermal parameters for all non-hydrogen atoms. The H atoms were included using riding models and direct assignment. The hydrogen atoms could not be found when the solvent, methanol, was used for recrystallization. This is likely due to the positional disorder that was modeled. The stereochemistry of C1 (R), C5 (R), and C9 (S) was determined via the orientation of the molecules. It is worth noting that there are A and B alerts observed in the cif. Each will be addressed here: The rotational disorder of a solvent water molecule resulted in an “alert A”, a CCDC qualifier which, in this molecule, suggests short hydrogen bonding distances to the protonated amine. The hydrogen bonding is likely due to rotations of the amine as well. The residual electron density observed in alert B is due to a further positional disorder of the methanol mentioned above. Attempts to resolve this disorder with additional parts were not successful. The proton on the water without an acceptor is due to the water molecule having a rotational disorder between two molecules that it interacts with, as discussed in alert A. The outlier reflections were checked, and there were no major missing or overly intense peaks (~10 sigma). Nothing is out of the ordinary with each reflection, and no changes were made. None of the alert C or G errors were a cause for concern, but they were checked on a case-by-case basis to verify there were no major problems. Tables of the X-ray spectroscopic data were included in the Supplementary Materials.

The atomic coordinates for **23** have been deposited with the Cambridge Crystallographic Data Centre, deposition number 2336627. Copies of the data can be obtained, free of charge, upon sending an application to CCDC, 12 Union Road, Cambridge, CB2 1EZ, UK (fax: +44(0)-1223-336033 or e-mail: deposit@ccdc.cam.ac.uk).

## 4. Conclusions

None of the C9 amino compounds were as potent as the formerly reported stereoisomers bearing a 1*R*,5*R*,9*S*-OH or a 1*R*,5*S*,9*R*-methyl substituent at C9 in 5-phenylmorphan [3]. Only the cyanomethyl compound 1*R*,5*S*,9*R*-**35** had nanomolar potency among the various compounds with a nitrogen atom; it was about 7x more potent than morphine and was found to have high MOR efficacy. An amino substituent directly attached at C9 had lowered potency in comparison with the generally more potent methylaminomethyl compounds with a secondary amino function situated one carbon away from C9. Both the stereochemistry of the molecule and that of the C9 substituent were found to affect potency and efficacy. Most of these new compounds were either fully efficacious or had little or no efficacy at the MOR, with two exceptions: the primary amine partial agonist 1*R*,5*R*,9*R*-**23** and the saturated cyanomethyl compound 1*S*,5*R*,9*R*-**38**. The former was only moderately potent, while the latter was twice as potent as morphine. That compound, 1*S*,5*S*,9*R*-**38**, had potency and efficacy like those of a compound that had been previously noted, bearing a C9 hydroxyethyl substituent at C9. That compound was found to have fewer adverse side effects than those displayed by analgesics like fentanyl [13]. Among the enantiomers, it was common to have a ten-fold difference in potency. Only a few compounds were weak MOR antagonists (compounds **3**, **8**, and **41**), and only one compound, 1*S*,5*R*,9*S*-**46**, was found to be a potent KOR agonist (KOR EC_50_ = 18 nM (% E_max_ = 103%)). Two compounds, **33** and **35**, were potent DOR agonists, but neither was efficacious at DOR (DOR % E_max_ = 69% and 35%, respectively).

## Figures and Tables

**Table 1:** Oxime reduction.

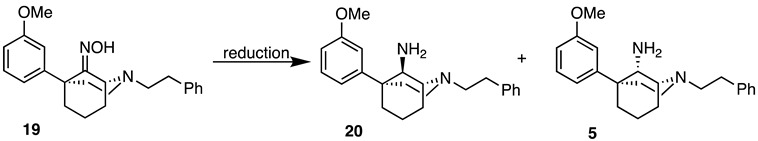		
Reductant	Diastereomeric Ratio (20:5)	Yield
Red-Al	1:2	15% (pure **20**)
H-Cube (H_2_, 10% Pd/C)	1:1	52% (mixture)
LAH	1.5:1	37% (pure **20**)

**Table 2:** Opioid receptor activity measured in the forskolin-induced cAMP accumulation assay ^a^.

Compd#	Molecular Structure	MOR		DOR		KOR	
		Agonist	Antagonist ^b^	Agonist	Antagonist ^c^	Agonist	Antagonist ^d^
		EC_50_ ± SEM (nM) (%E_max_ ± SEM)	IC_50_ ± SEM (nM) (%I_max_ ± SEM)	EC_50_ ± SEM (nM) (%E_max_ ± SEM)	IC_50_ ± SEM (nM) (%I_max_ ± SEM)	EC_50_ ± SEM (nM) (%E_max_ ± SEM)	IC_50_ ± SEM (nM) (%I_max_ ± SEM)
1*S*,5*S*,9*R*-**3**	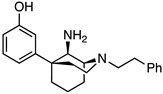	>10,000	709 ± 248 (114.8 ± 5.5%)	N/D	N/D	>10,000	N/D
1*R*,5*R*,9*S*-**6**	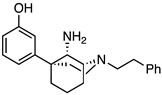	24 ± 8 (91 ± 6%)	N/D	690 ± 188 (39.4 ± 3.5%)	>10,000	>10,000	>10,000
1*S*,5*S*,9*R*-**8**	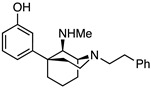	>10,000	178 ± 81 (136.5 ± 20.1%)	N/D	N/D	>10,000	N/D
1*R*,5*R*,9*S*-**10**	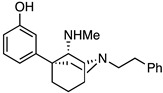	82.8 ± 27.5 (27.5 ± 8%)	>10,000	N/D	N/D	N/D	N/D
1*S*,5*S*-**15**	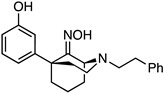	229 ± 67 (90.6 ± 4.6%)	N/D	N/D	N/D	>10,000	N/D
1*R*,5*R*-**17**	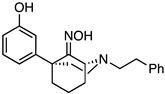	22.12 ± 10.42 (99.3 ± 3.2%)	N/D	157 ± 72 (32.2 ± 5.0%)	1077.30 ± 463.66 (66.5 ± 6.0%)	146 ± 19 (21.2 ± 4.3%)	>10,000
1*S*,5*S*,9*S*-**22**	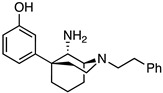	94.1 ± 47.8 (53.2 ± 12.2%)	N/D	N/D	N/D	N/D	N/D
1*R*,5*R*,9*R*-**23**	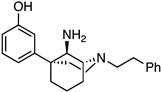	26.1 ± 6.18 (80.7% ± 12.9%)	N/D	186 ± 12.5 (46.6 ± 1.6%)	N/D	>10,000	>10,000
1*S*,5*S*-**28**	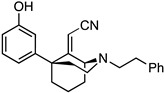	27.2 ± 4.1 (100.4% ± 2%)	N/D	1518 ± 385 (51.4 ± 3.5%)	N/D	>10,000	>10,000
1*R*,5*R*-**33**	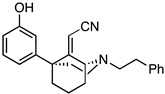	1.8 ± 0.7 (100.1% ± 1.4%)	N/D	8.1 ± 1.8 (69.3 ± 6.0%)	N/D	>10,000 (observed max response > 50%)	N/D
1*S*,5*R*,9*S*-**34**	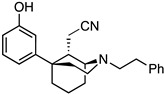	27.3 ± 1.1 (100.8 ± 0.8%)	N/D	575 ± 127 (61.5 ± 1.8%)	N/D	>10,000	>10,000
1*R*,5*S*,9*R*-**35**	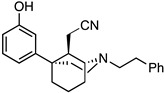	0.85 ± 0.14 (98.8 ± 0.8%)	N/D	4.3 ± 2.6 (35.4% ± 10.9%)	94.52 ± 29.46 (35.1 ± 2.6%)	>10,000	>10,000
1*S*,5*R*,9*R*-**38**	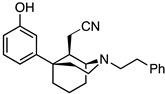	2.5 ± 1.2 (89.6 ± 3.2%)	N/D	55.0 ± 3.9 (46.8 ± 8.4%)	N/D	>10,000	44 ± 4 (97 ± 0.1%)
1*R*,5*S*,9*S*-**41**	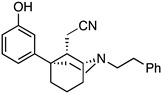	>10,000	318 ± 143 (124.7 ± 13.9%)	N/D	N/D	N/D	N/D
1*S*,5*R*,9*S*-**46**	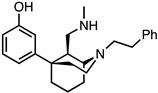	2.4 ± 0.3 (102.7 ± 0.6%)	N/D	126 ± 40 (77.7 ± 2.2%)	N/D	17.9 ± 6.99 (103.2 ± 6.2%)	N/D
1*S*,5*R*,9*R*-**47**	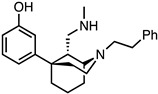	3.66 ± 0.26 (101.9 ± 0.6%)	N/D	416.45 ± 21.07 (84.7 ± 3.9%)	N/D	95.69 ± 16.99 (93.4 ± 3.5%)	N/D
1*R*,5*S*,9*R*-**52**	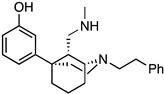	43.3 ± 13.2 (93.6% ± 4.2%)	N/D	502 ±125 (62% ± 2.6%)	N/D	210 ± 92 (57% ± 9.4%)	N/D
1*R*,5*S*,9*S*-**53**	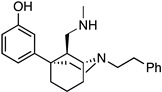	15.9 ± 4 (97 ± 3%)	N/D	43.9 ± 22.2 (17.4 ± 1.0%)	1631.98 ± 882.10 (109.3 ± 5.2%)	>10,000	>10,000
	**STANDARDS**						
	Morphine	5.80 ± 0.32 (102.0 ± 0.1%)					
	Naltrexone	2.14 ± 1.2 (29.61 ± 6.4%)	10.8 ± 0.9 (103.5 ± 0.6%)	>10,000	295.1 ± 47.5 (99.4 ± 1.1%)	0.64 ± 0.32 (56.5 ± 7.2%)	5.53 ± 1.02 (41.3 ± 6.8%)
	U50488H					0.31 ± 0.03 (99.5 ± 0.3%)	
	SNC80			1.66 ± 0.16 (78.6 ± 1.7%)			
	nor-BNI						2.34 ± 0.29 (102.4 ± 1.2%)
	Naltrindole				1.11 ± 0.22 (106.8 ± 4.8%)		

^a^ Inhibition of forskolin-induced cAMP accumulation; cAMP Hunter^TM^ Chinese hamster ovary cells (CHO-K1) that express human μ-opioid receptor (OPRM1), human κ-opioid receptor (OPRK1), and human δ-opioid receptor (OPRD1) were used for the forskolin-induced cAMP accumulation assay to determine potency and efficacy of the compounds following the previously established methods [17]; to determine % efficacy in forskolin-induced cAMP assays, background readouts of the vehicle control were subtracted from all treatment readouts and then normalized to the forskolin control. Data were then analyzed in GraphPad Prism 8 (GraphPad, La Jolla, CA, USA) using nonlinear regression; values are expressed as the mean ± SEM of at least three independent experiments; N/D = not determined. ^b^ MOR antagonist potency (IC_50_) determined versus EC_90_ of fentanyl; degree of antagonism (I_max_) normalized to naltrexone. ^c^ DOR antagonist potency (IC_50_) determined versus EC_50_ of SNC80; degree of antagonism (I_max_) normalized to naltrexone. ^d^ KOR antagonist potency (IC_50_) determined versus EC_90_ of U50488H; degree of antagonism (Imax) normalized to *nor*-BNI.

## Data Availability

The data presented in this study are available in this article or in the Supplementary Materials.

## Supplementary Materials

The following supporting information can be downloaded at https://www.mdpi.com/article/10.3390/molecules29091926/s1. Figures S1–S37: 1H and 13C-NMR spectra of novel compounds; Tables S1–S6: X-ray spectroscopic data.

## Abbreviations

MOR	mu-opioid receptor
DOR	delta-opioid receptor
KOR	kappa-opioid receptor
cAMP	cyclic adenosine monophosphate
