# Supplementary material for: Functional Activity of Enantiomeric Oximes and Diastereomeric Amines and Cyano Substituents at C9 in 3-Hydroxy-N-phenethyl-5-phenylmorphans
Source: Molecules. 2024 Apr 23;29(9):1926. doi: 10.3390/molecules29091926 (PMC11085448; doi:10.3390/molecules29091926)
Supplement: Supplementary file 1 [file molecules-29-01926-s001.zip › Supplementary Materials_4-17-24.pdf]

SUPLEMENTARY MATERIAL

Functional Activity of Enantiomeric Oximes and Diastereomeric Amines and Cyano Substituents at C9 in 3-Hydroxy-*N*-phenethyl-5-phenylmorphans

Hudson G. Roth[1], Madhurima Das[1], Agnieszka Sulima[1], Dan Luo[2], Sophia Kaska[2], Thomas E. Prisinzano[2], Andrew T. Kerr,[3] Arthur E. Jacobson[1,*], Kenner C. Rice[1,*]

[1]Drug Design and Synthesis Section, Molecular Targets and Medications Discovery Branch, Intramural Research Program, National Institute on Drug Abuse and the National Institute on Alcohol Abuse and Alcoholism, National Institutes of Health, Department of Health and Human Services, 9800 Medical Center Drive, Bethesda, MD 20892-3373, USA

[2]Department of Pharmaceutical Sciences, College of Pharmacy, University of Kentucky, 789 S. Limestone Street, Lexington, Kentucky 40536, USA

[3]Center for Biomolecular Science and Engineering, Naval Research Laboratory, Washington, DC 20375-0001, USA

*Correspondence: arthurj@nida.nih.gov (A.E.J.); kennerr@nida.nih.gov (K.C.R.)

Tel.: +1-301-451-5028 (A.E.J.); +1-301-451-4799 (K.C.R.)

Supplementary Data

^{1}H and ^{13}C NMR spectra	Figures S1-S37	Pages 2-38, respectively
X-ray Spectroscopic data	Tables S1-S7	Pages 39-49, respectively

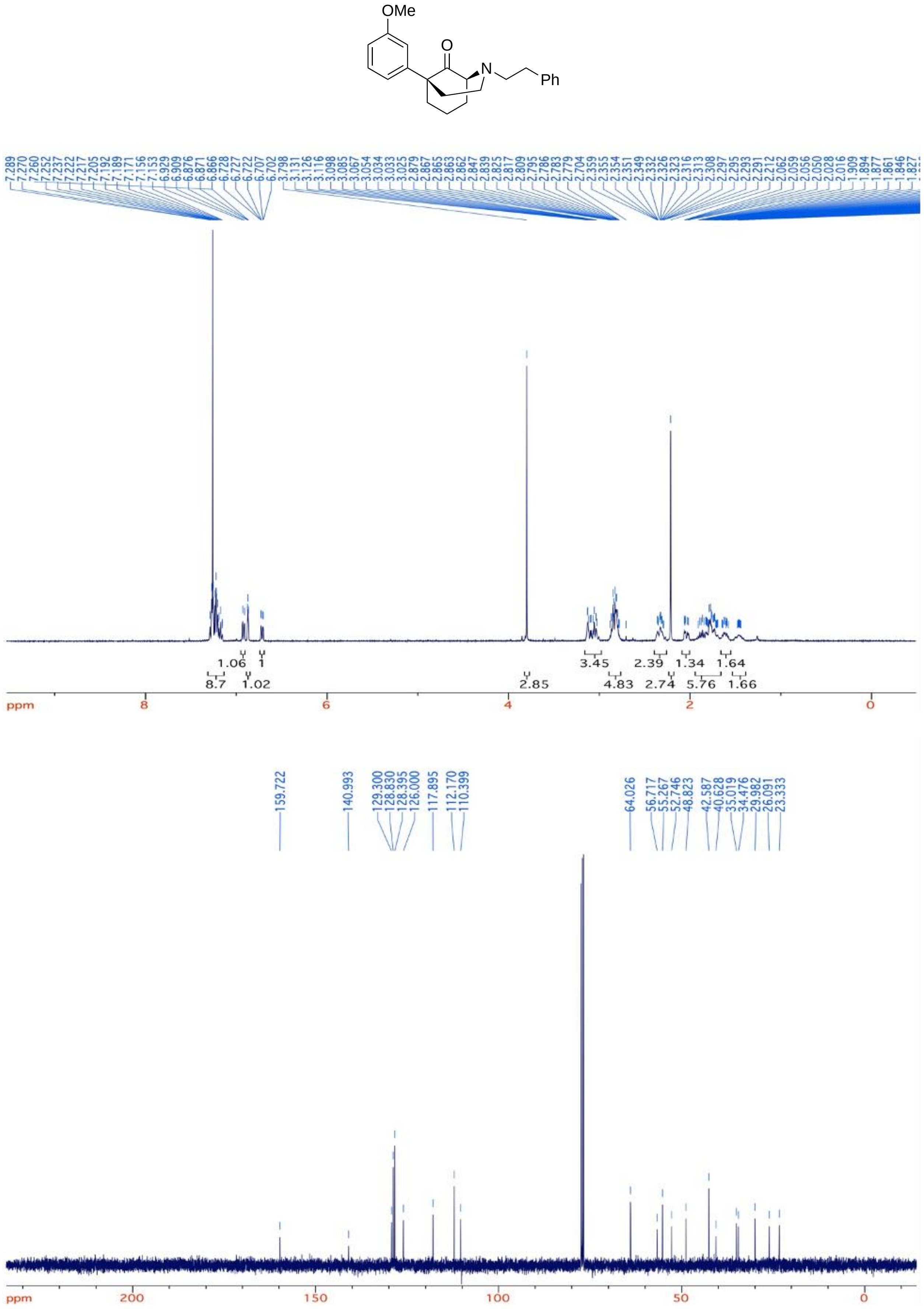

Figure S1. ^{1}H NMR and ^{13}C NMR of 1*S*,5*S*-**1**

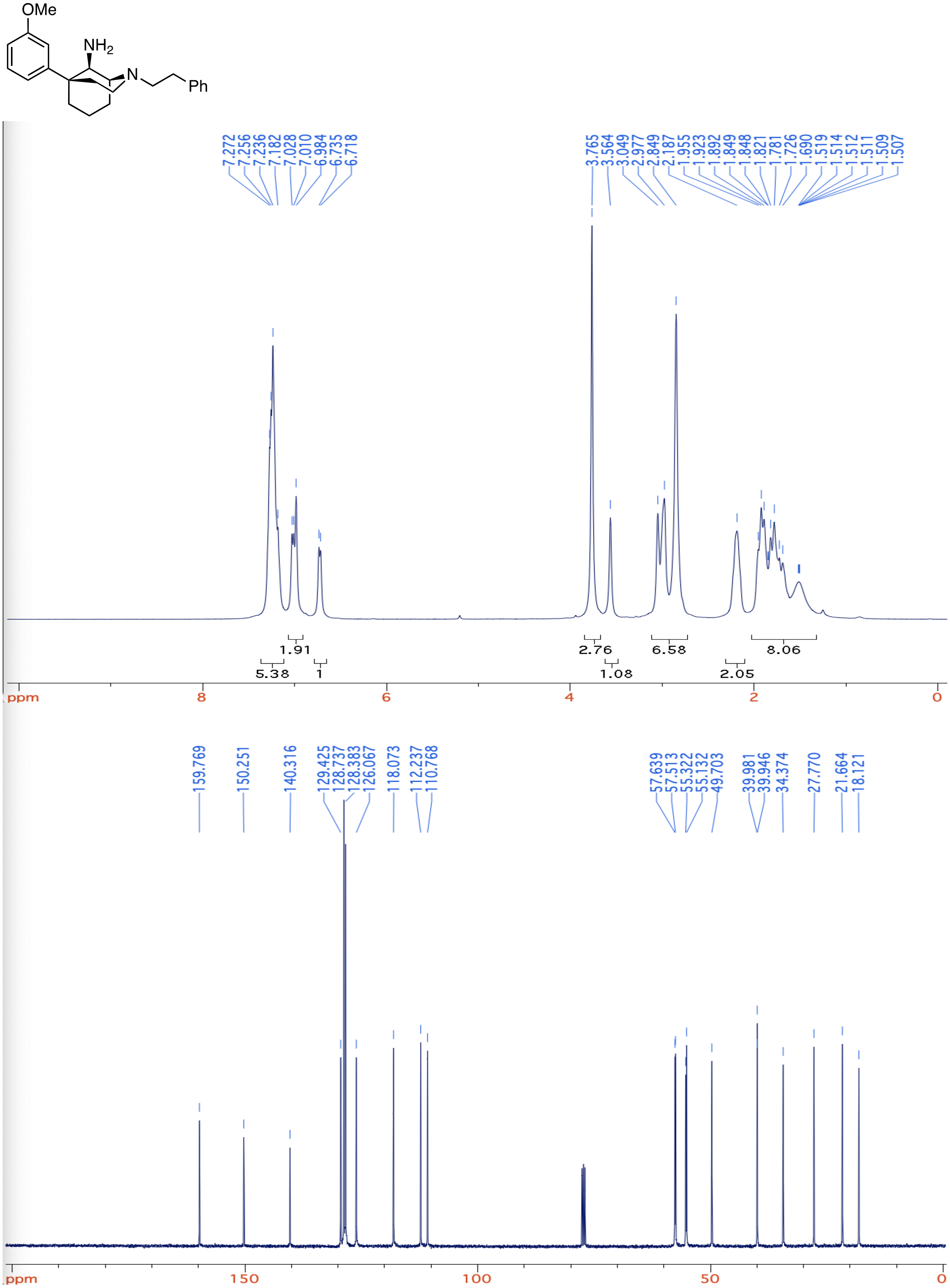

Figure S2. ^{1}H NMR and ^{13}C NMR of 1*S*,5*S*,9*R*-**2**

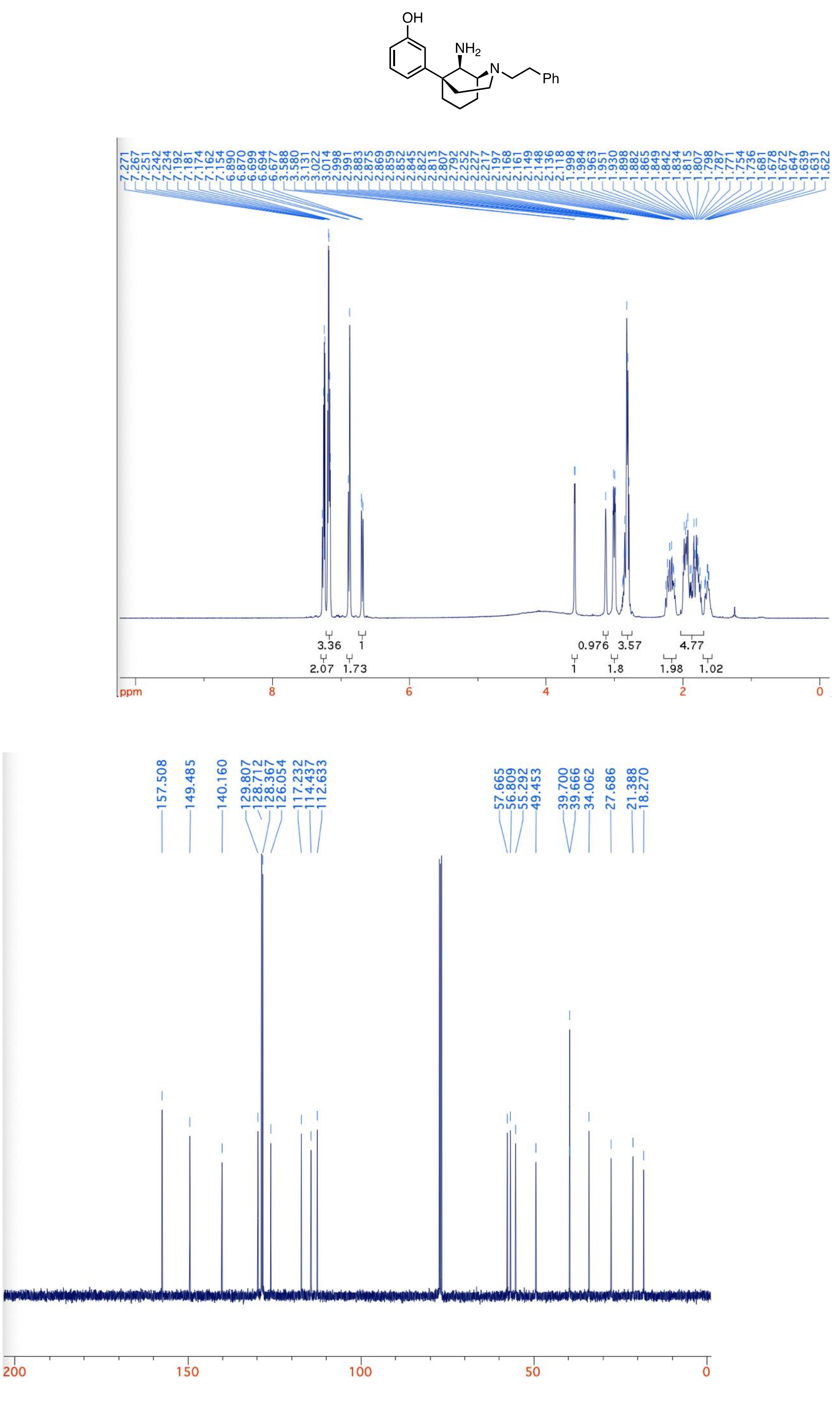

Figure S3. ^{1}H NMR and ^{13}C NMR of 1*S*,5*S*,9*R*-**3**

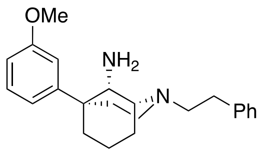

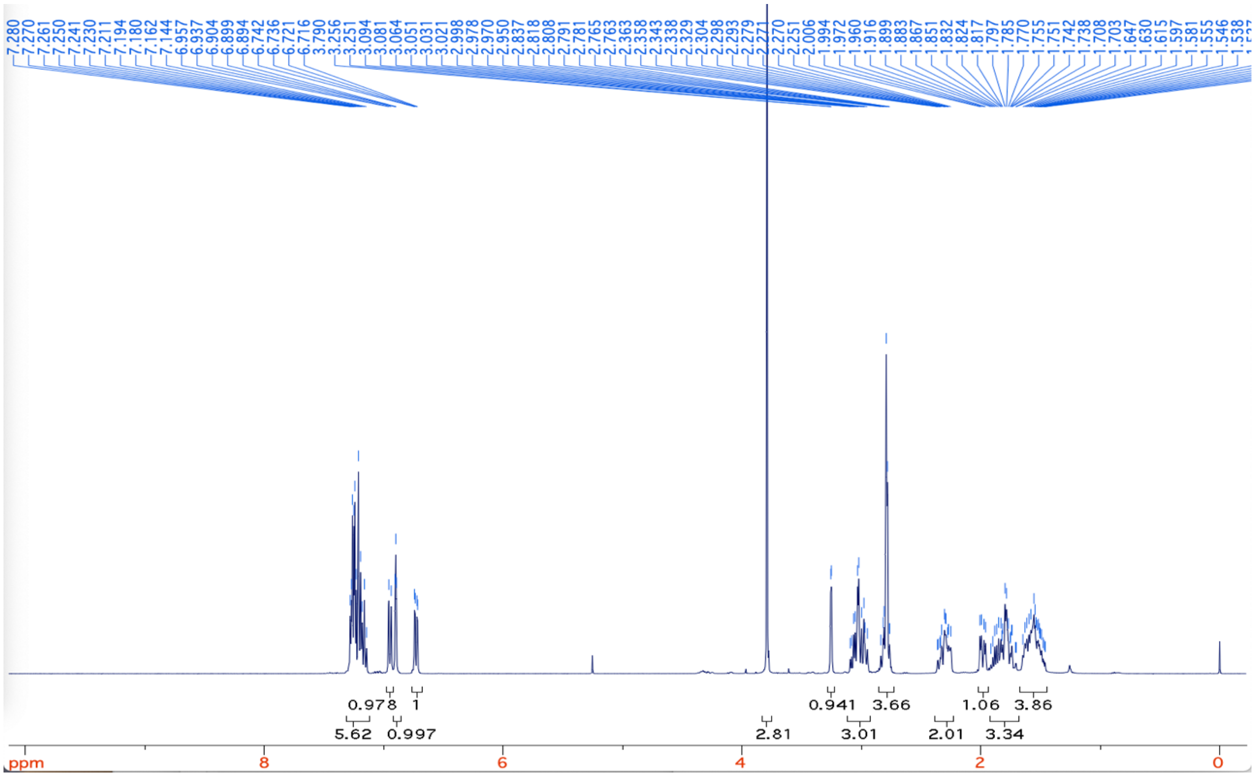

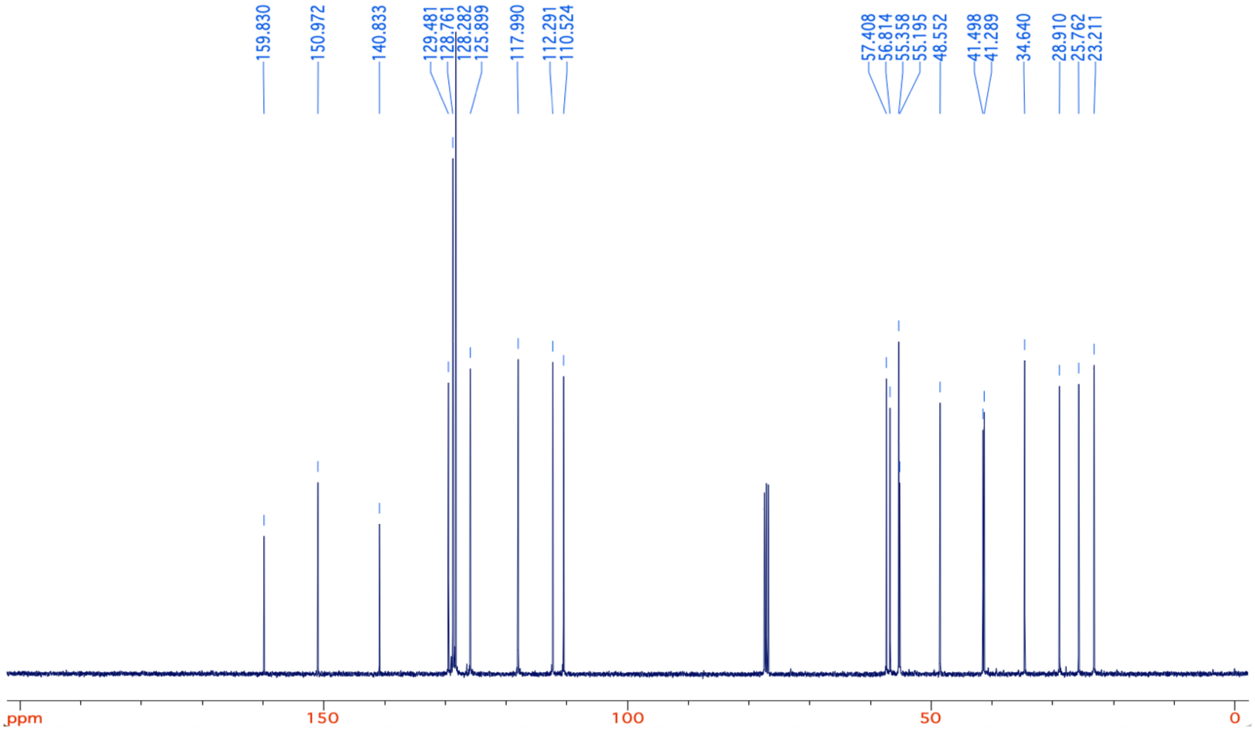

Figure S4. ^{1}H NMR and ^{13}C NMR of 1*R*,5*R*,9*S*-**5**

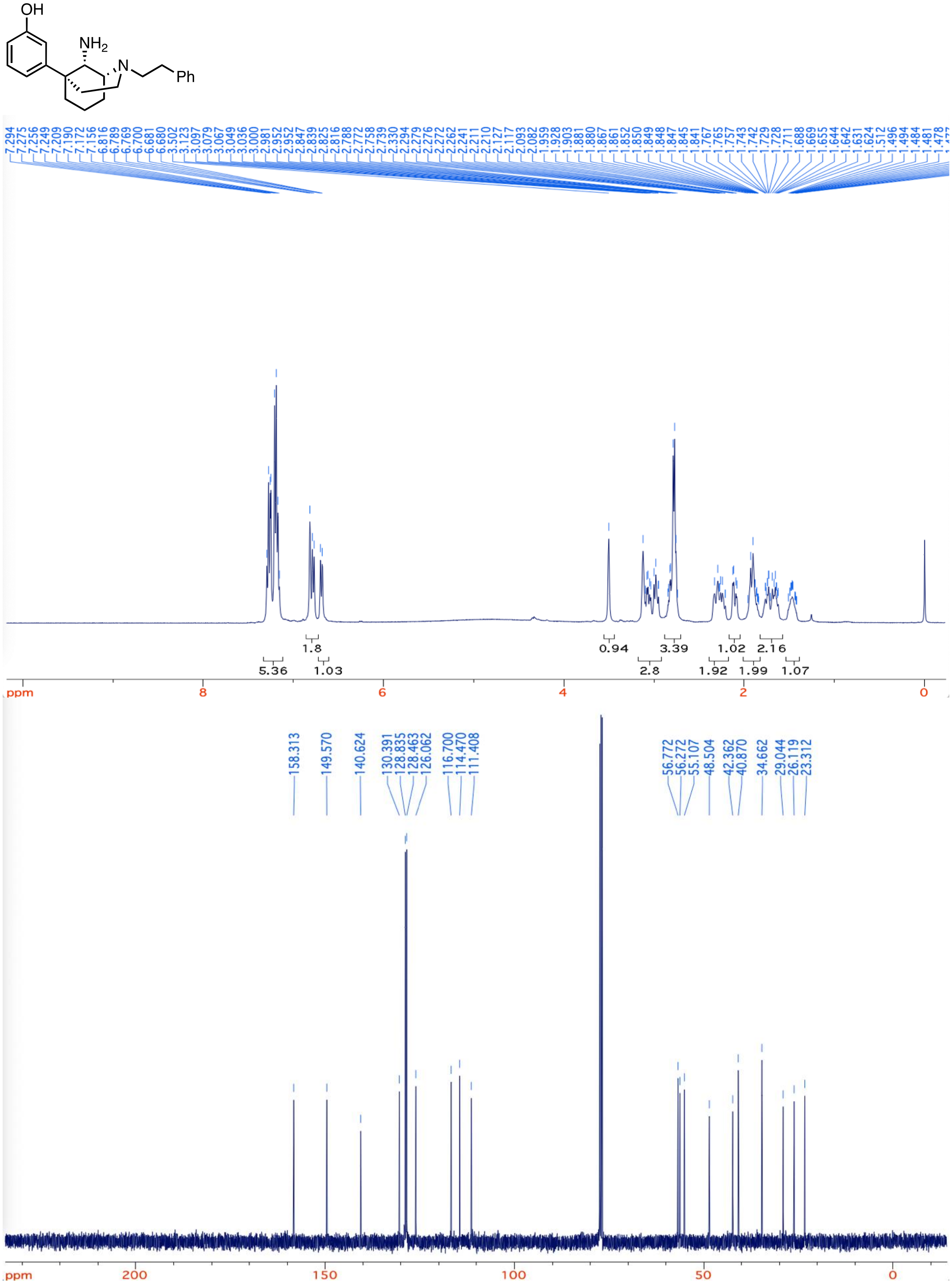

Figure S5. ^{1}H NMR and ^{13}C NMR of 1*R*,5*R*,9*S*-**6**

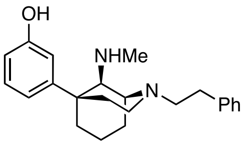

and

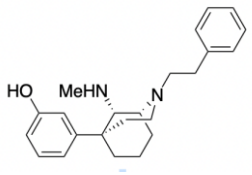

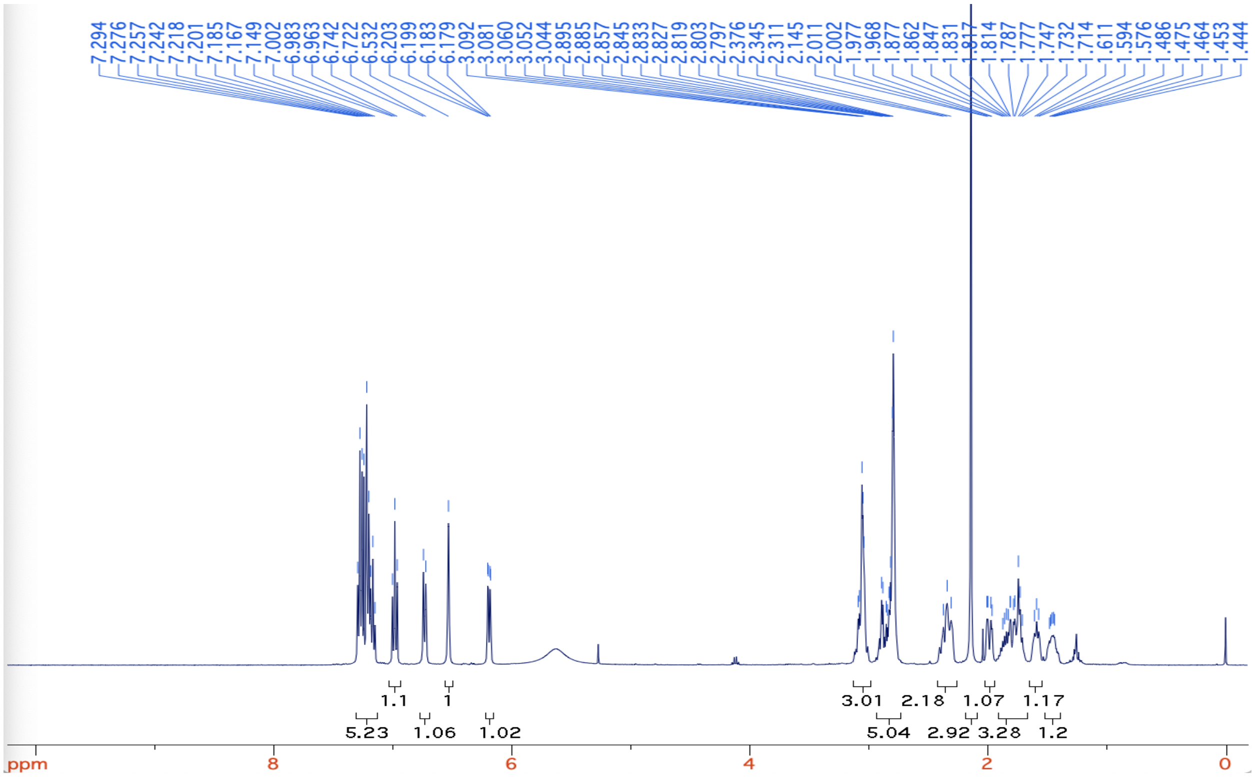

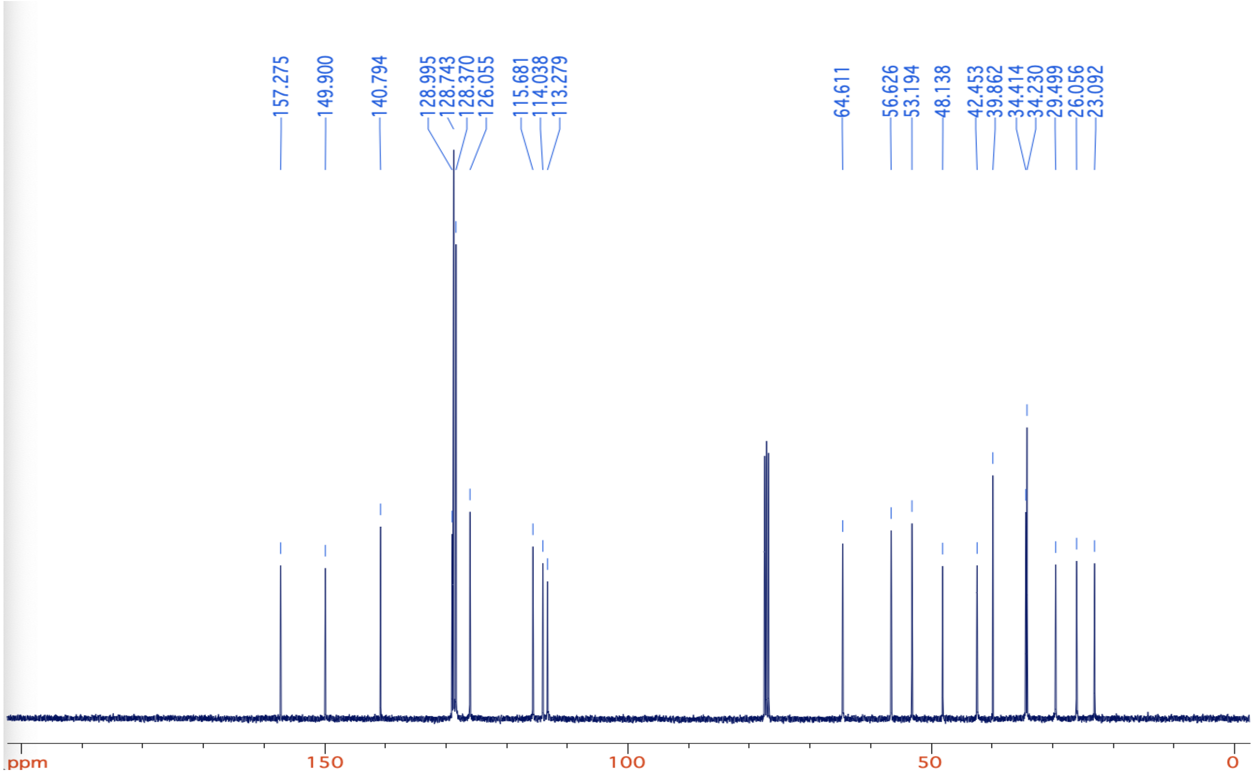

Figure S6. ^{1}H NMR and ^{13}C NMR of 1*S*,5*S*,9*R*-**8** and 1*R*,5*R,*9*S*-**10**

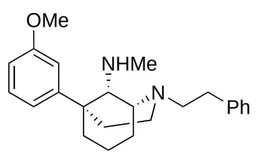

and

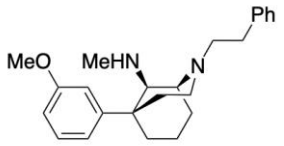

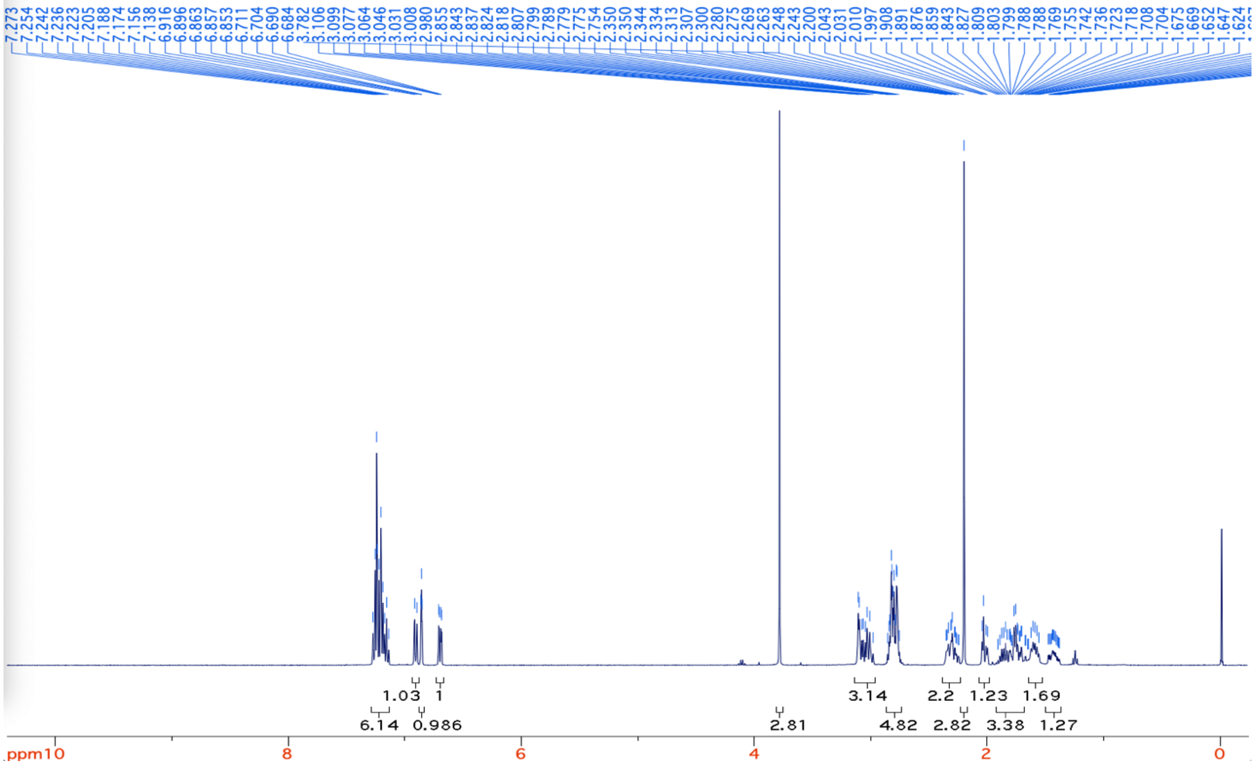

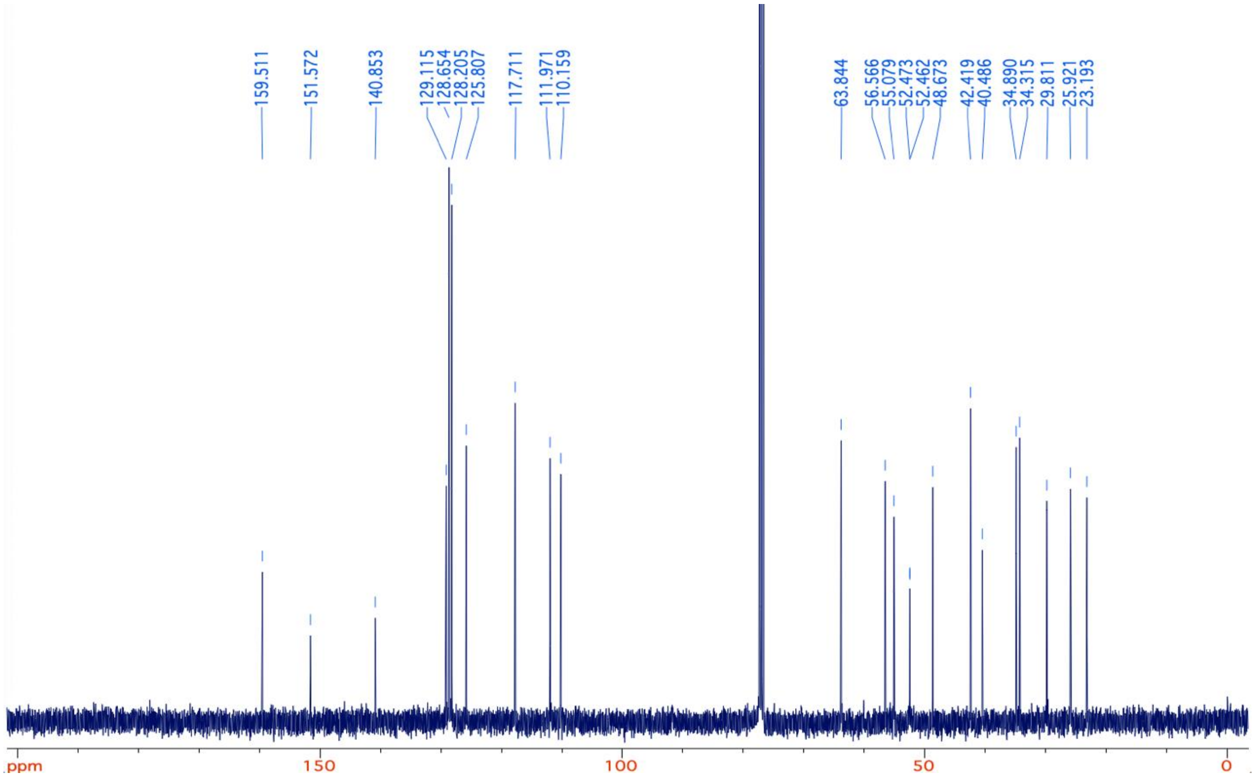

Figure S7. ^{1}H NMR and ^{13}C NMR of 1*S*,5*S*,9*R*-**7** and 1*R*,5*R*,9*S*-**9**

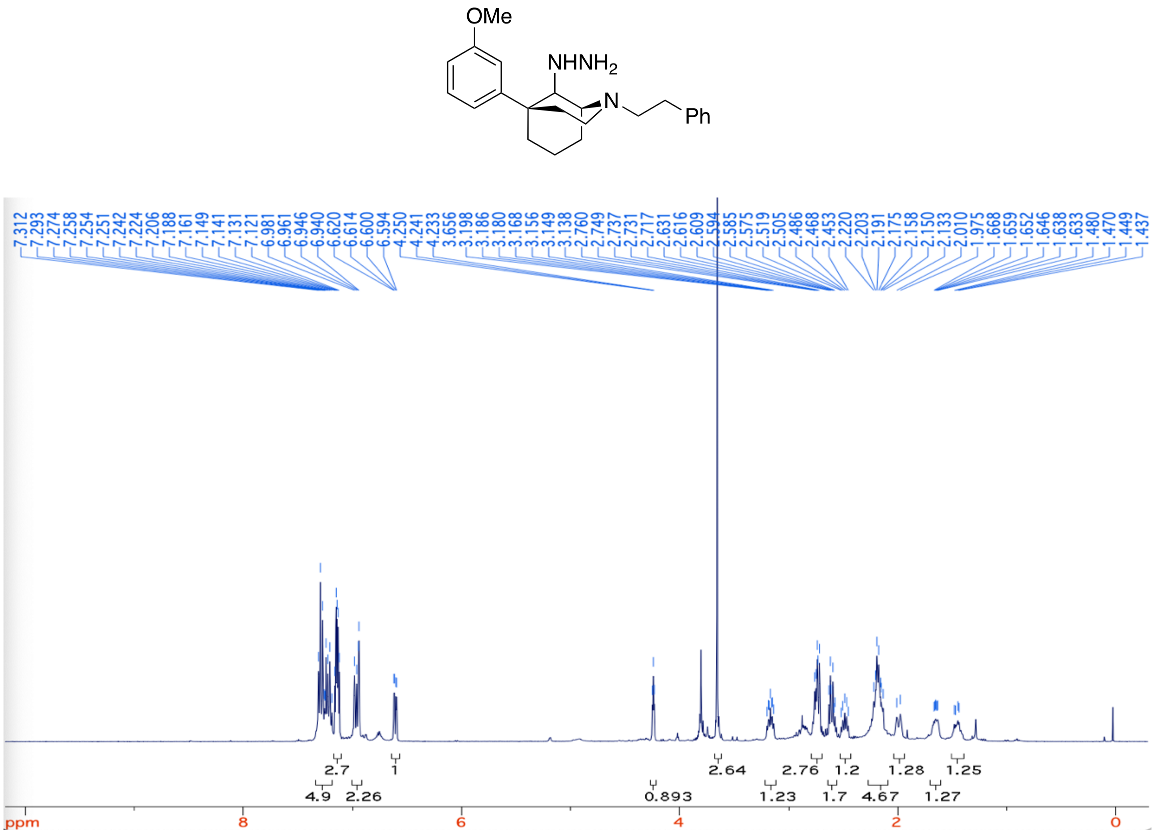

Figure S8. ^{1}H NMR of **11**

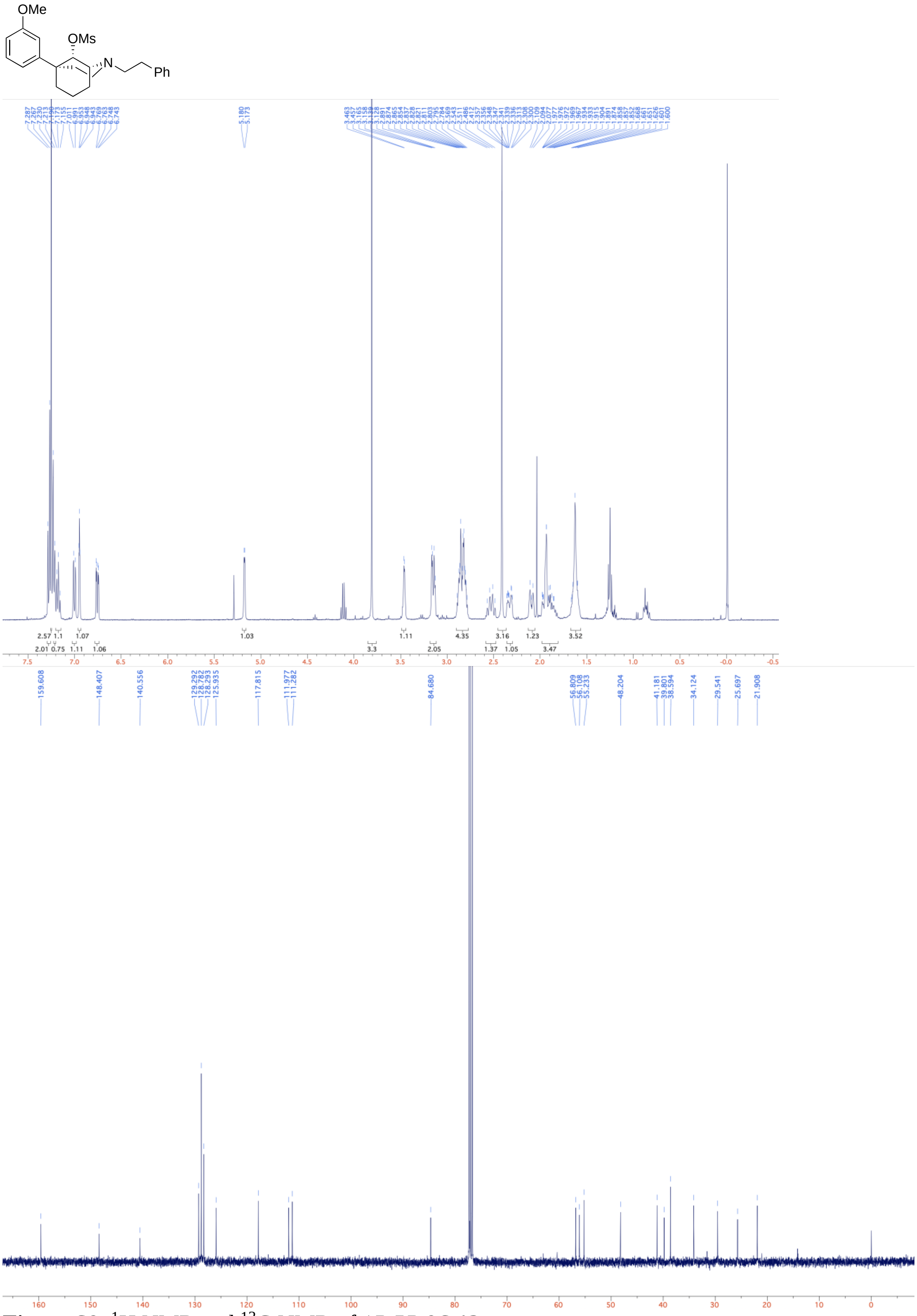

Figure S9. ^{1}H NMR and ^{13}C NMR of 1*R*,5*R*,9*S*-**13**

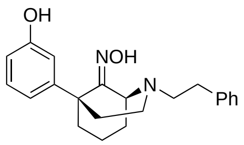

and

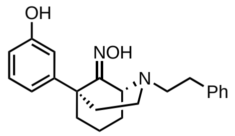

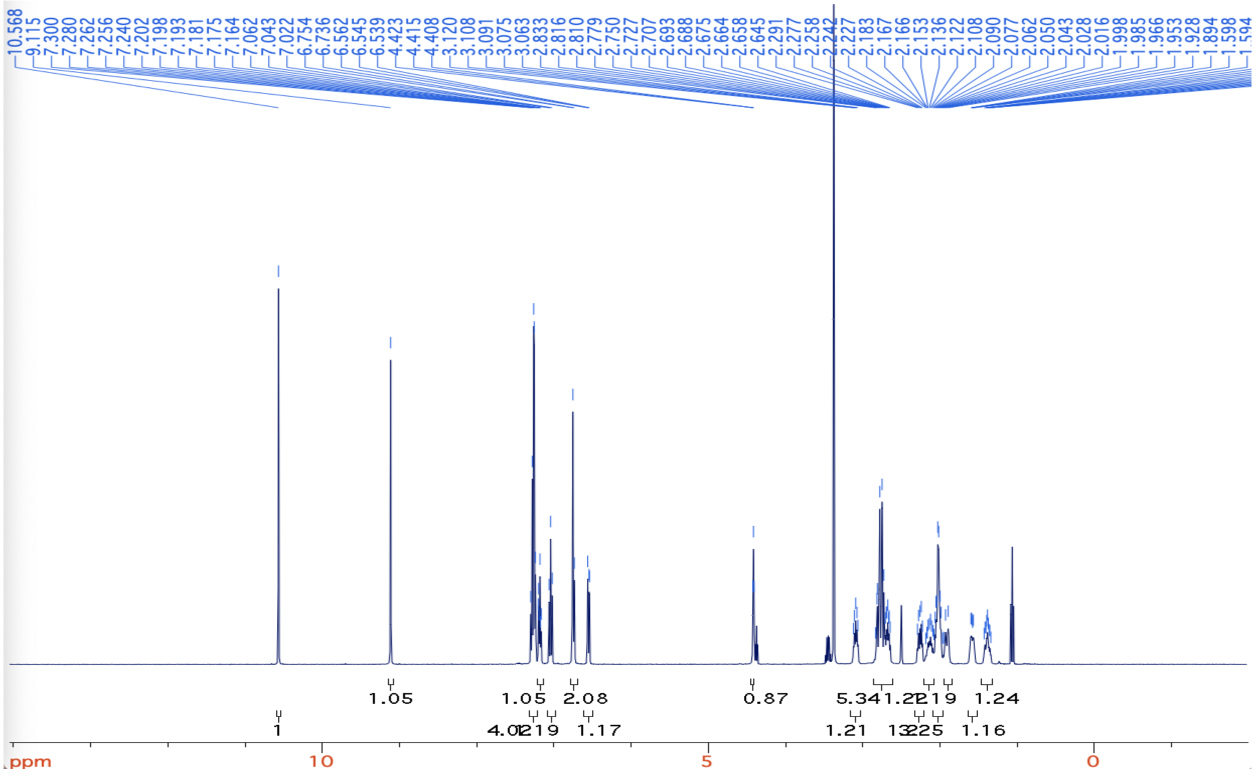

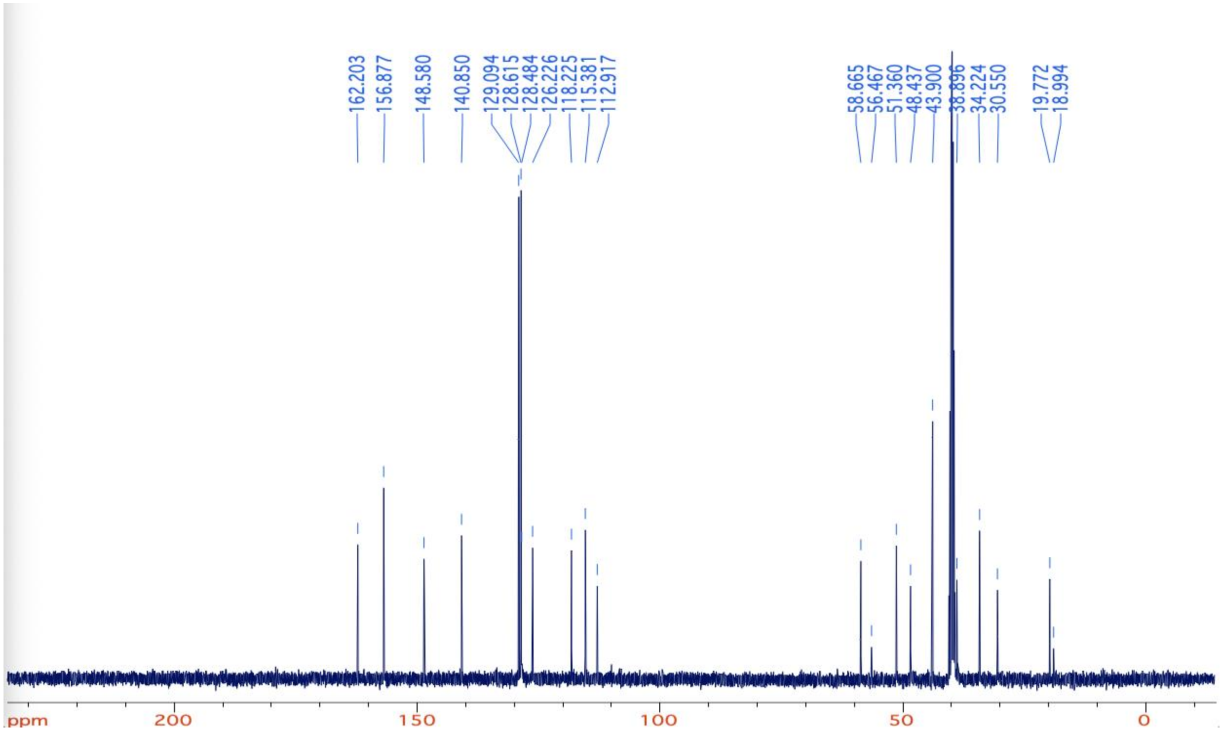

Figure S10. ^{1}H NMR and ^{13}C NMR of 1*S*,5*S*-**15** and 1*R*,5*R*-**17**

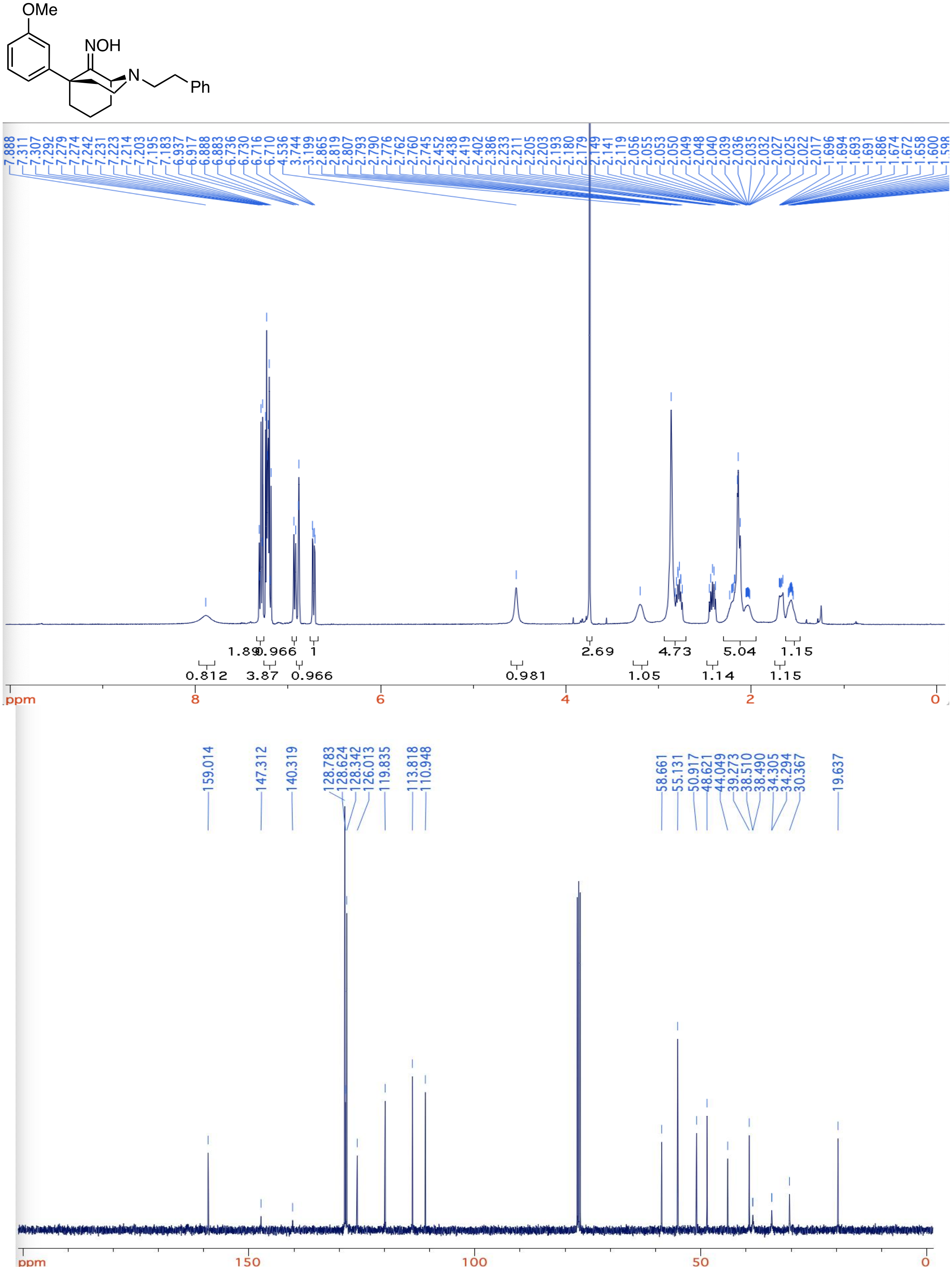

Figure S11. ^{1}H NMR and ^{13}C NMR of 1*S*,5*S*-**18**

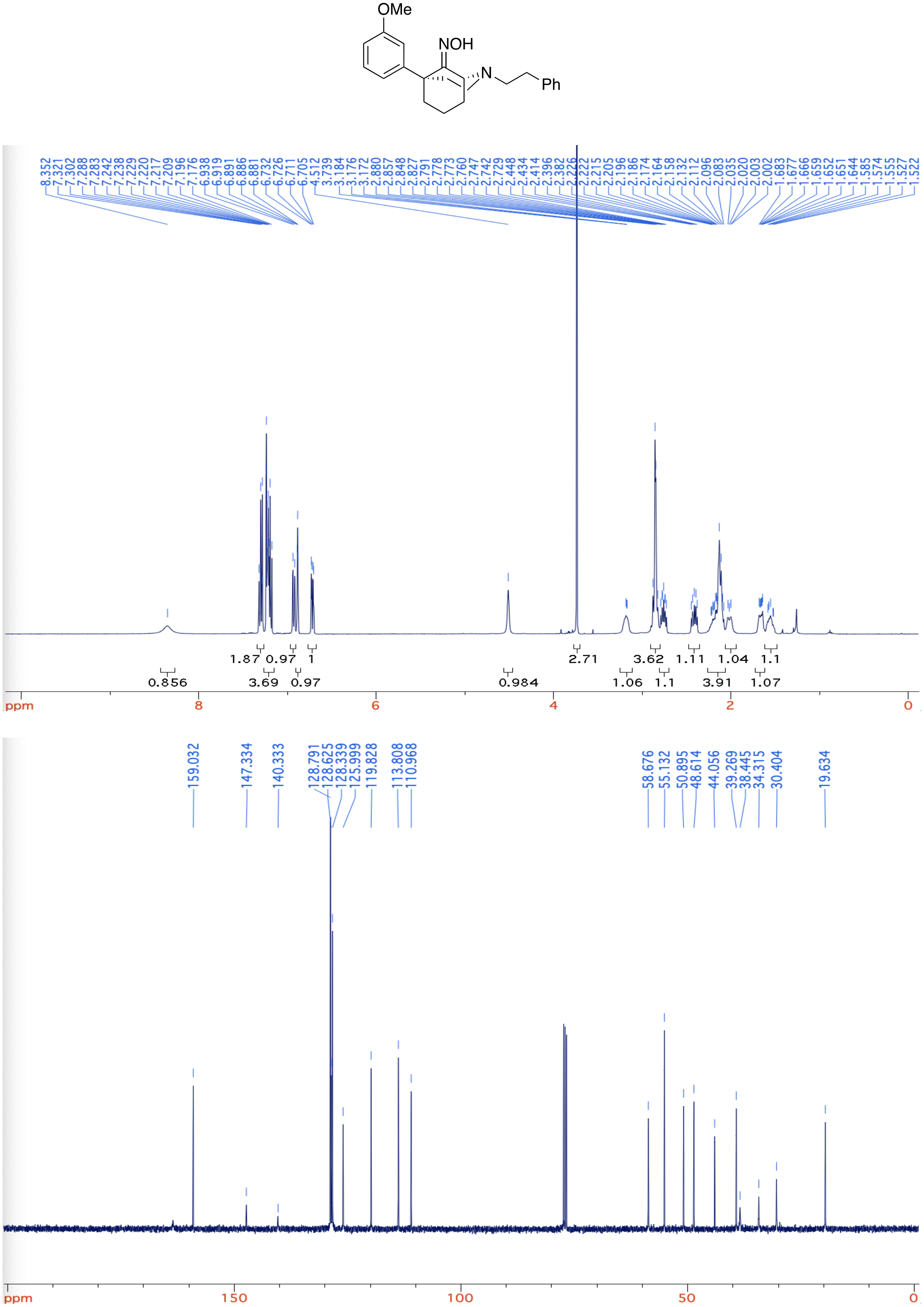

Figure S12. ^{1}H NMR and ^{13}C NMR of 1*R*,5*R*-**19**

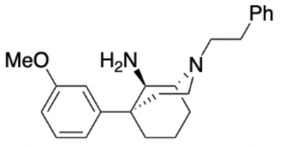

and

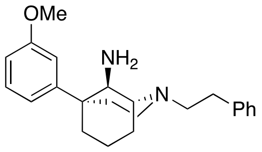

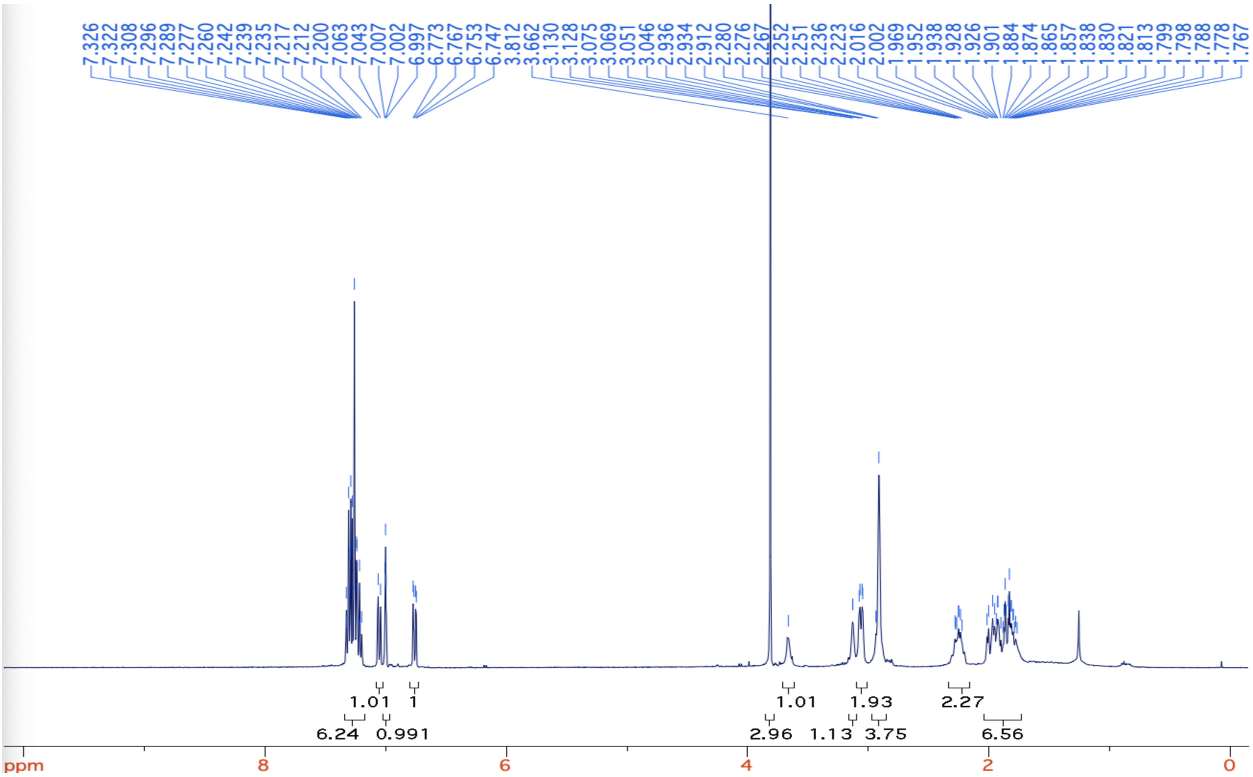

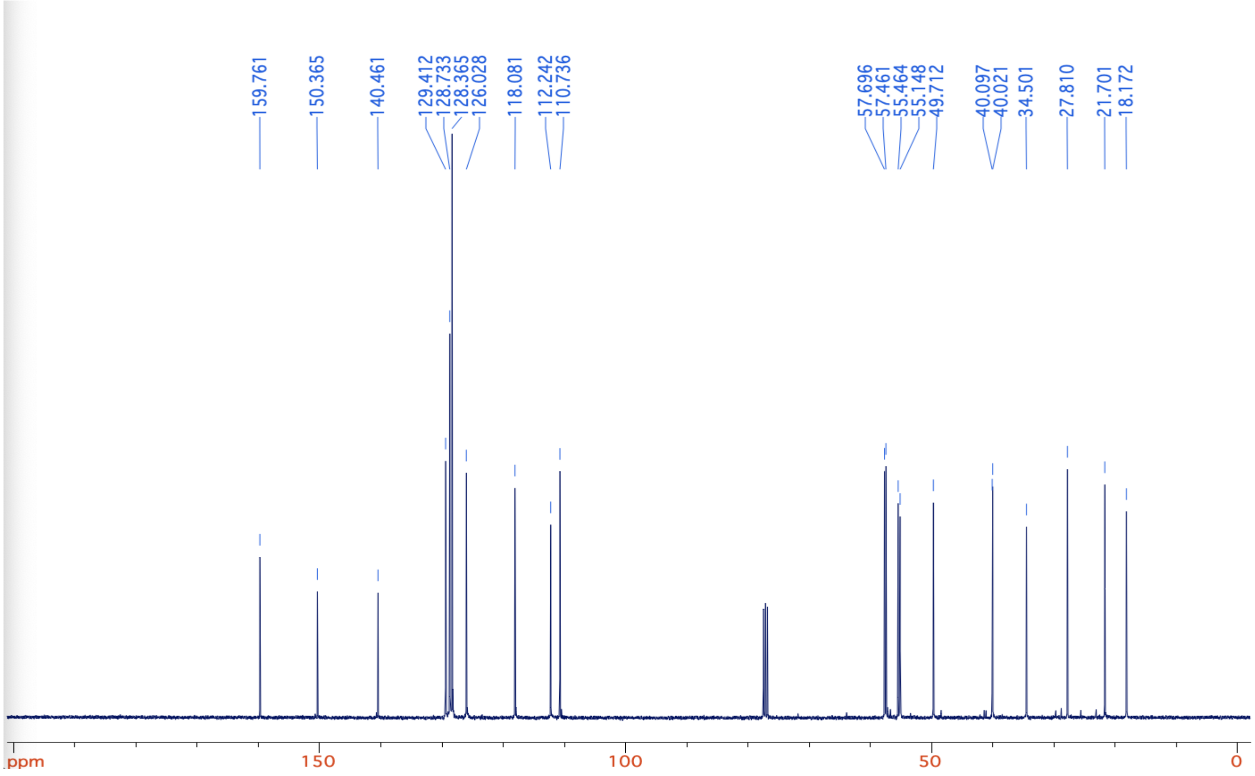

Figure S13. ^{1}H NMR and ^{13}C NMR of 1*R*,5*R*,9*R*-**20** and 1*S*,5*S*,9*S*-**21**

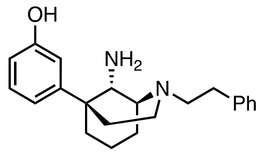

and

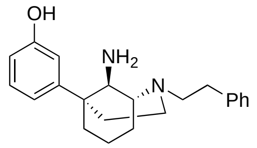

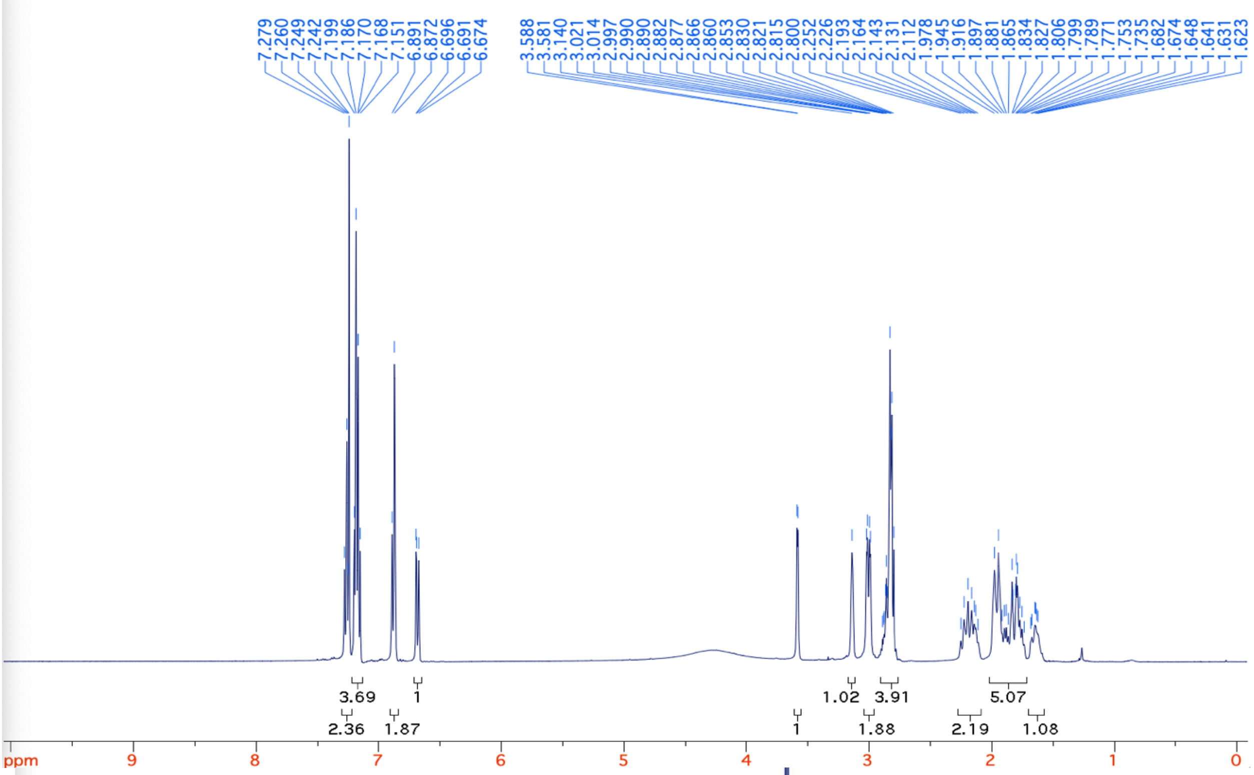

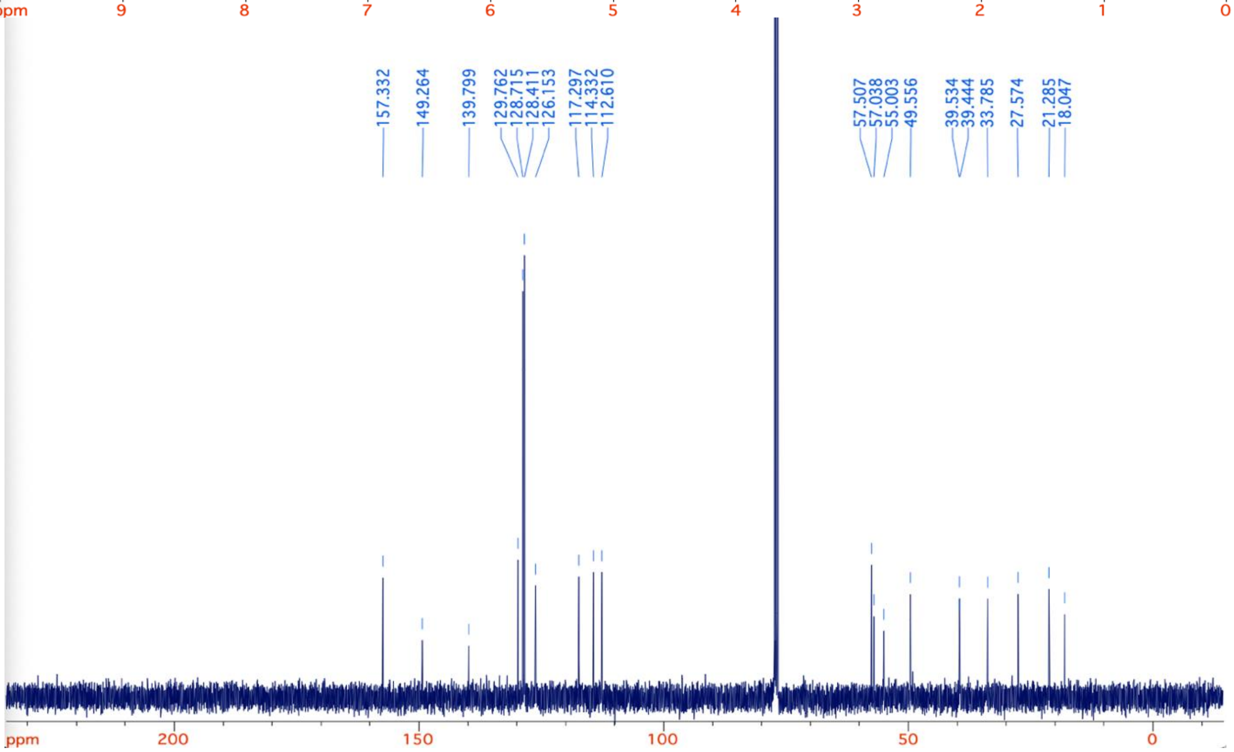

Figure S14. ^{1}H NMR and ^{13}C NMR of 1*S*,5*S*,9*S*-**22** and 1*R*,5*R*,9*R*-**23**

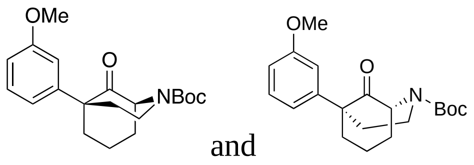

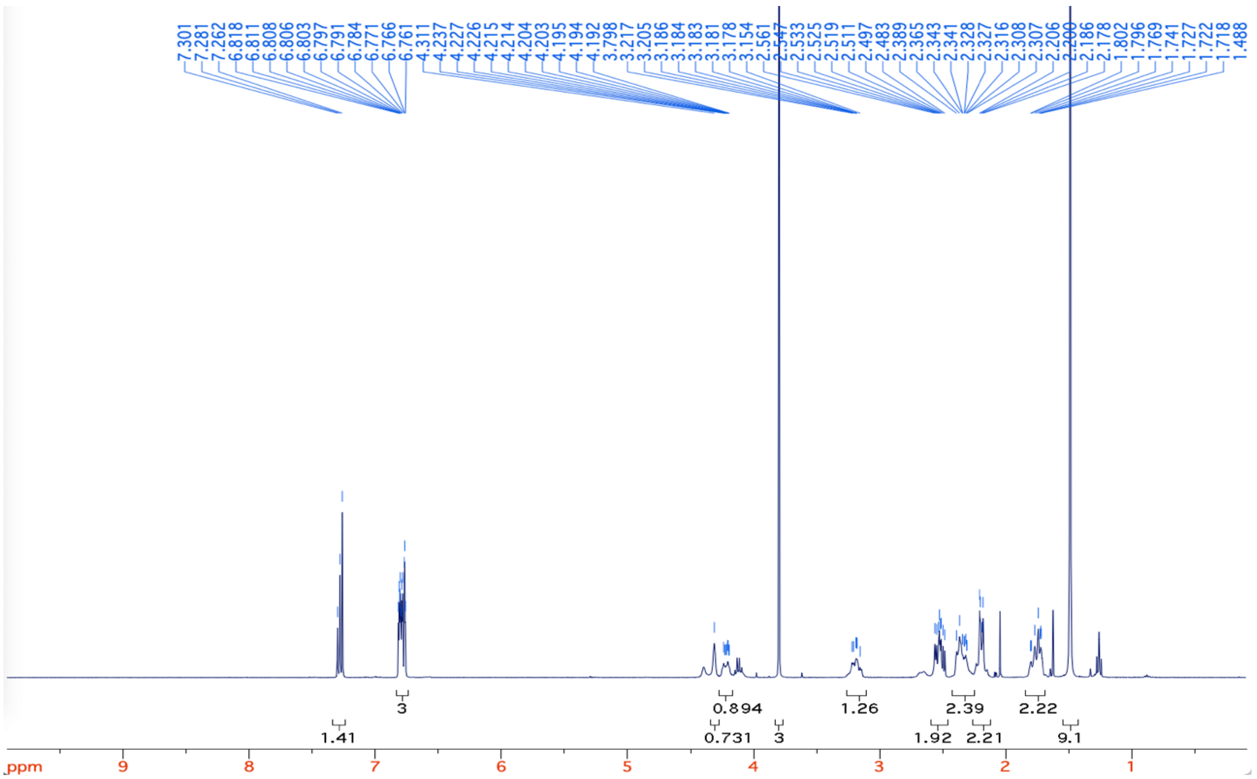

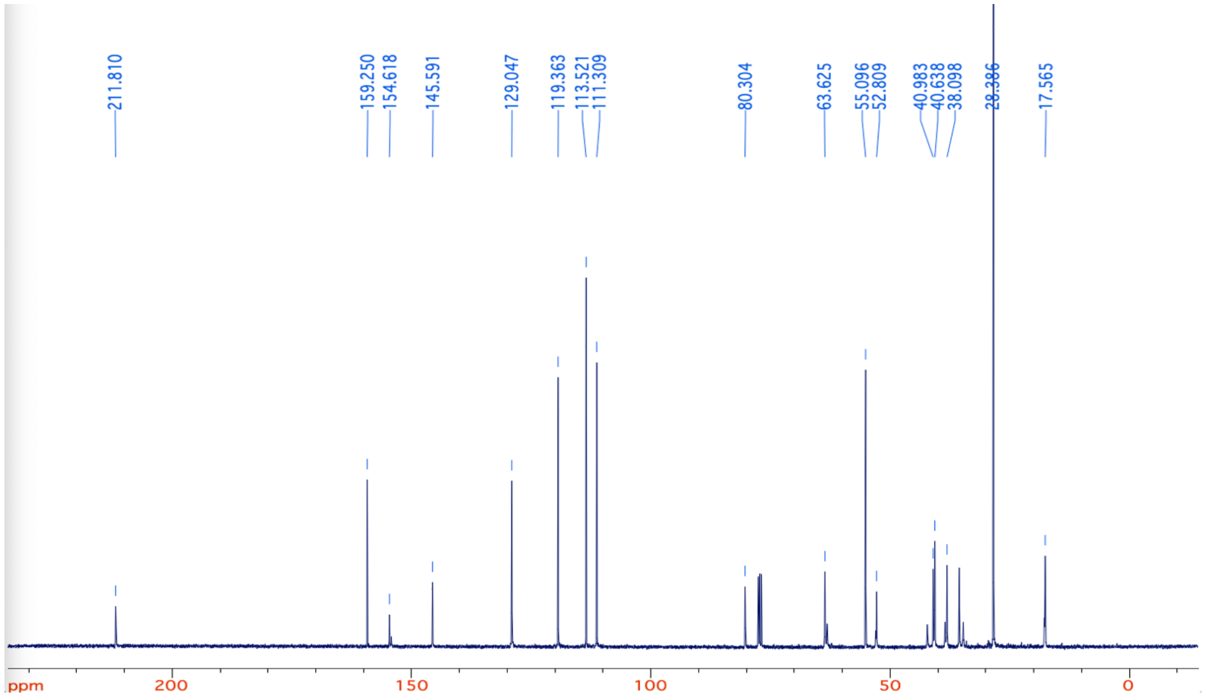

Figure S15. ^{1}H NMR and ^{13}C NMR of 1*S*,5*S*-**25** and 1*R*,5*R*-**30**

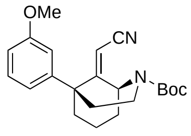

and

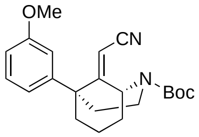

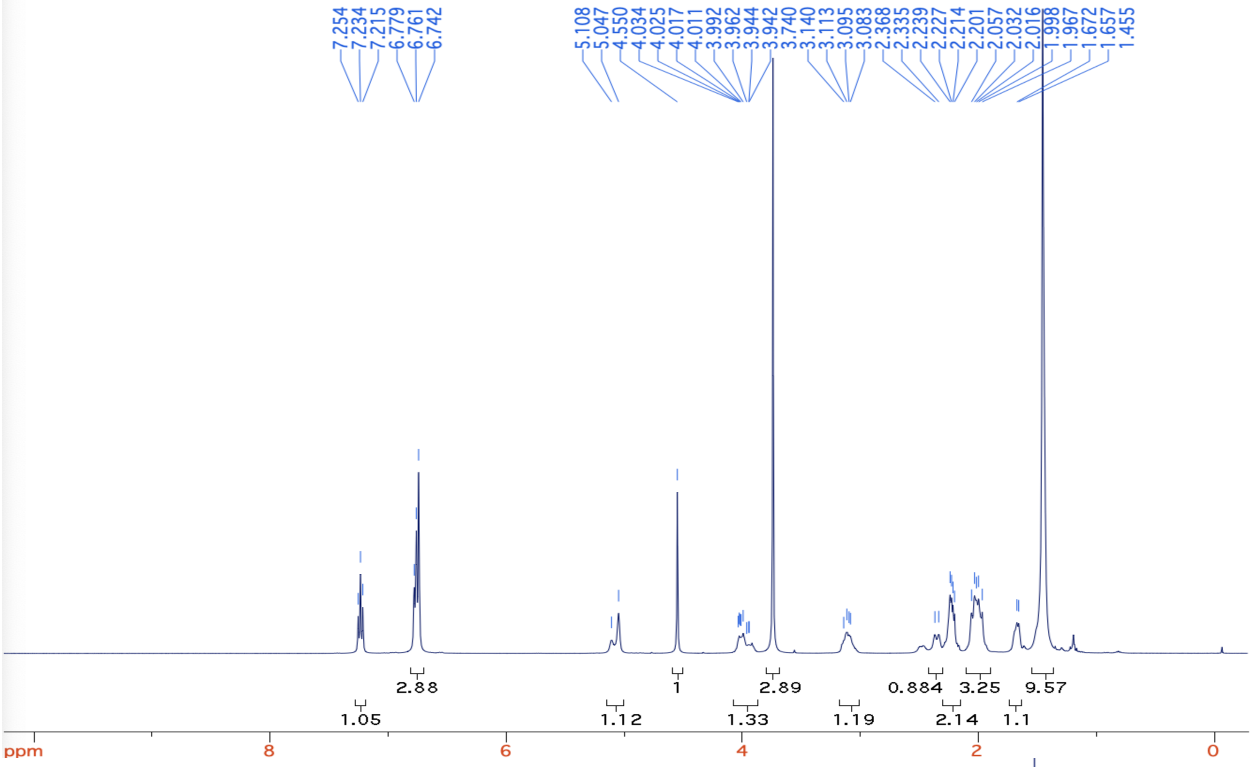

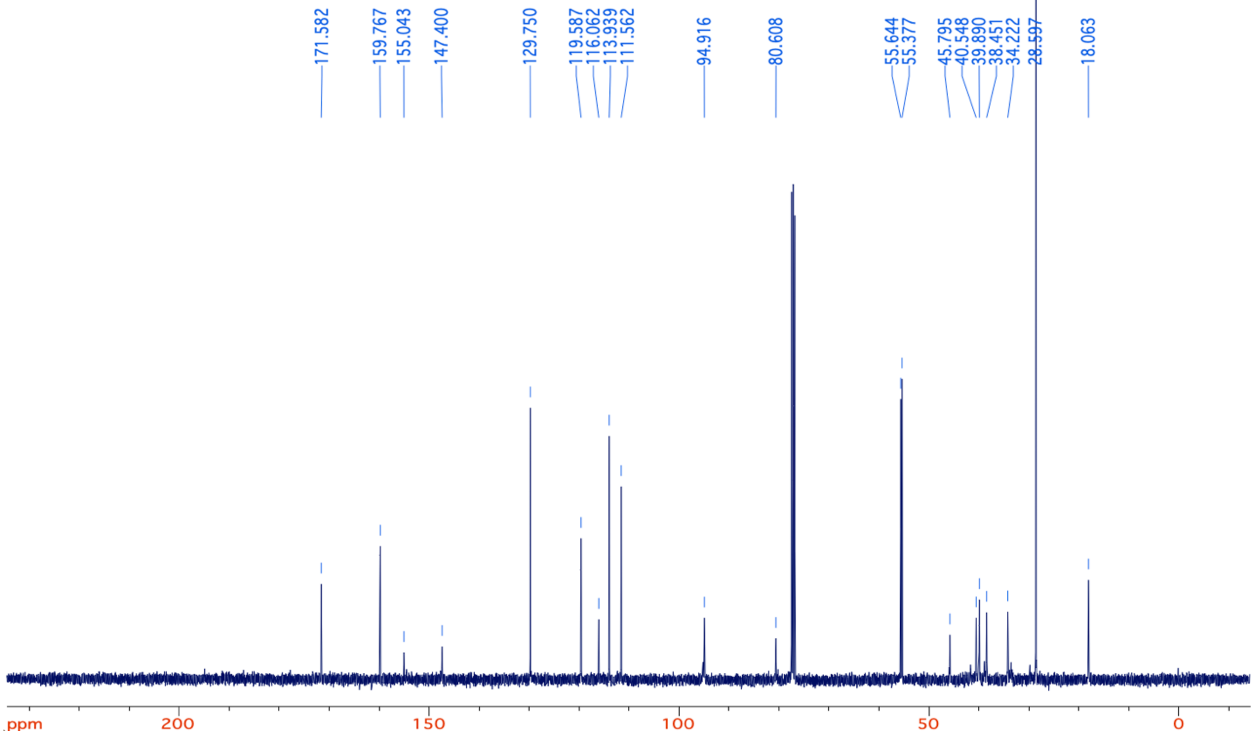

Figure S16. ^{1}H NMR and ^{13}C NMR of 1*S*,5*S*-**26** and 1*R*,5*R*-**31**

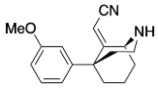

and

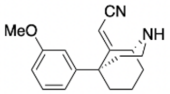

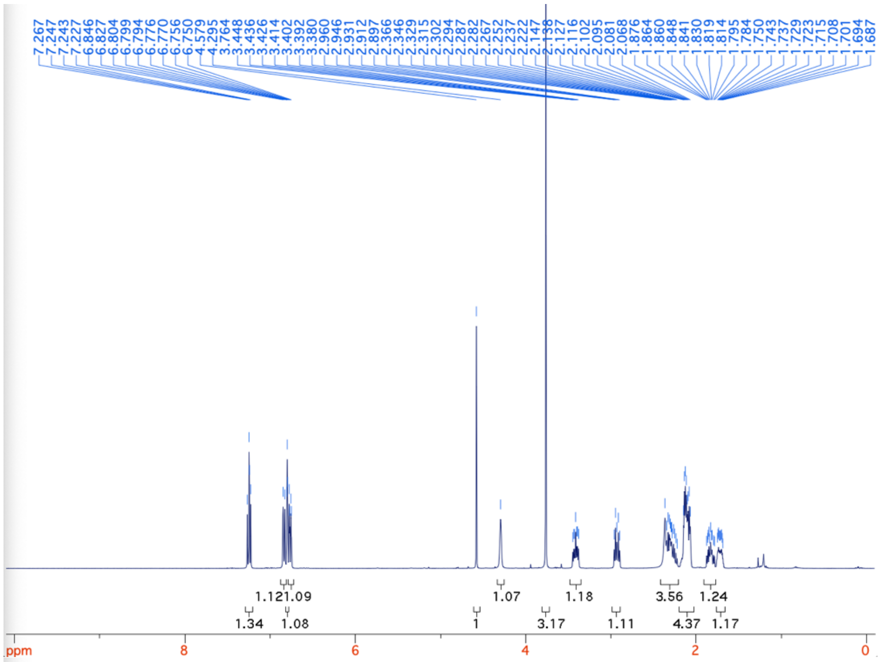

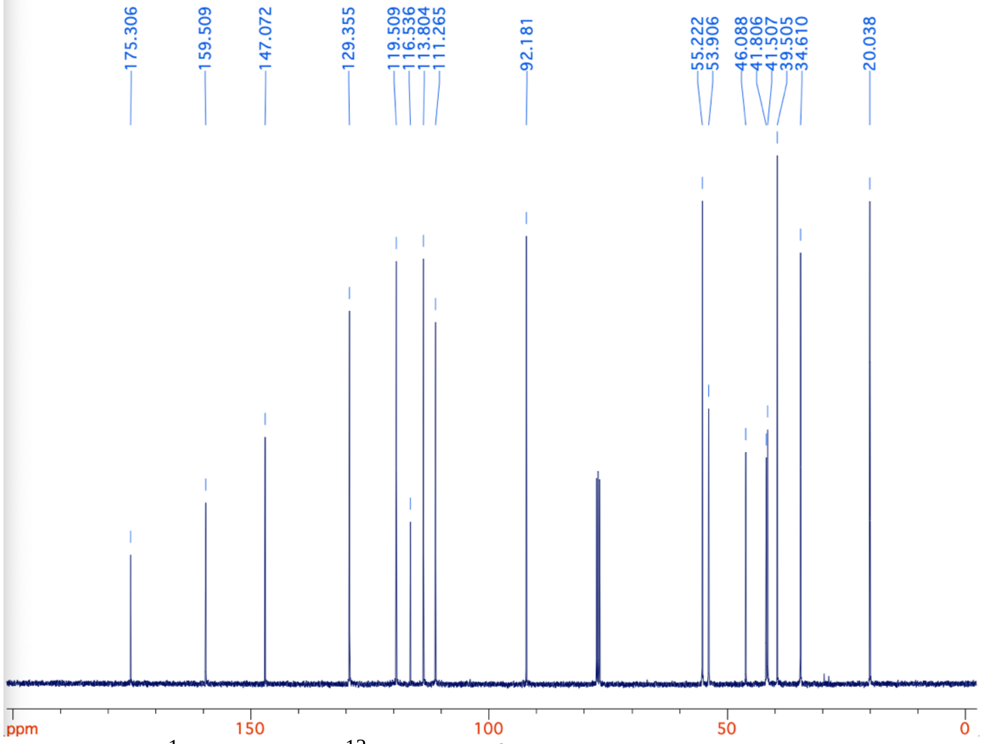

Figure S17. ^{1}H NMR and ^{13}C NMR of intermediate secondary amines towards 1*S*,5*S*-**27** and 1*R*,5*R*-**32**

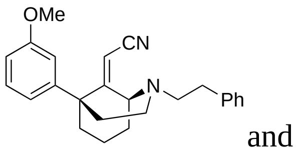

and

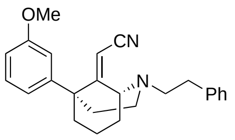

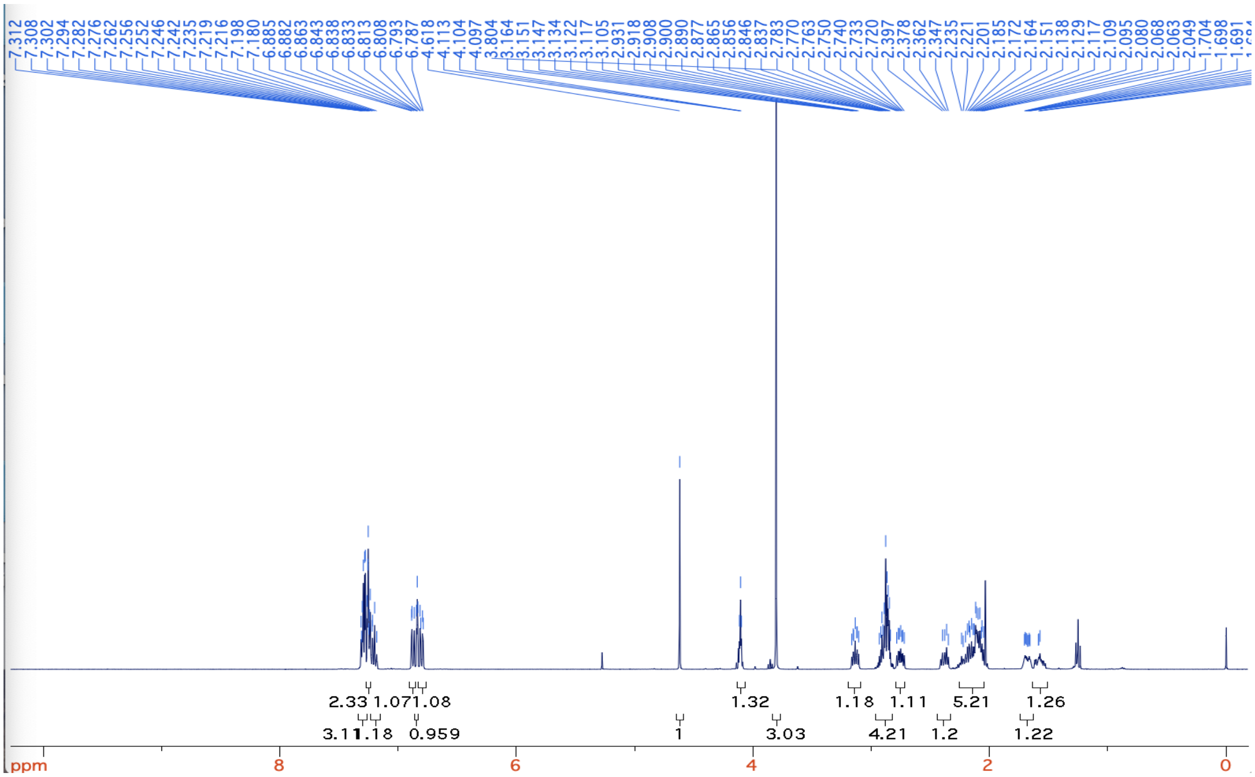

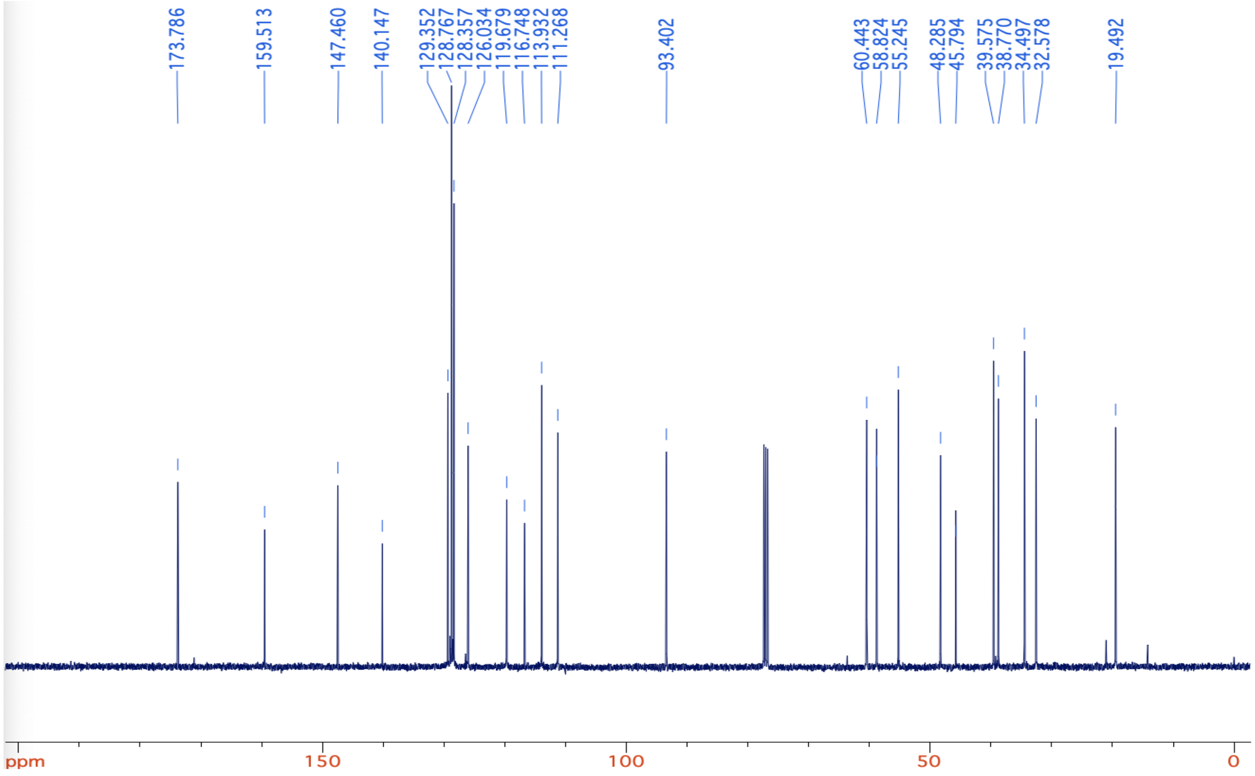

Figure S18. ^{1}H NMR and ^{13}C NMR of 1*S*,5*S*-**27** and 1*R*,5*R*-**32**

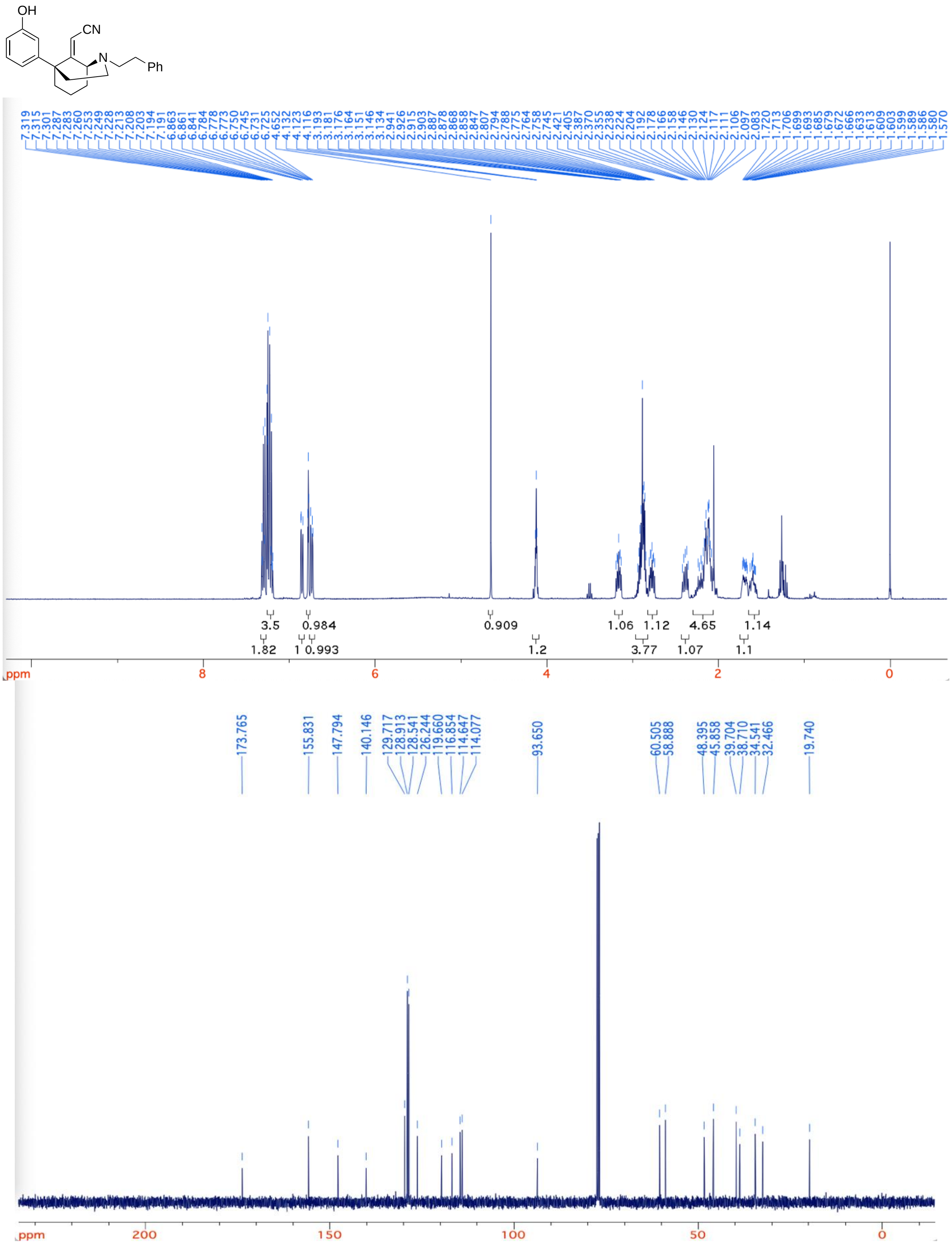

Figure S19. ^{1}H NMR and ^{13}C NMR of 1*S*,5*S*-**28**

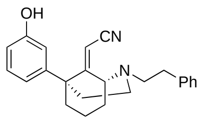

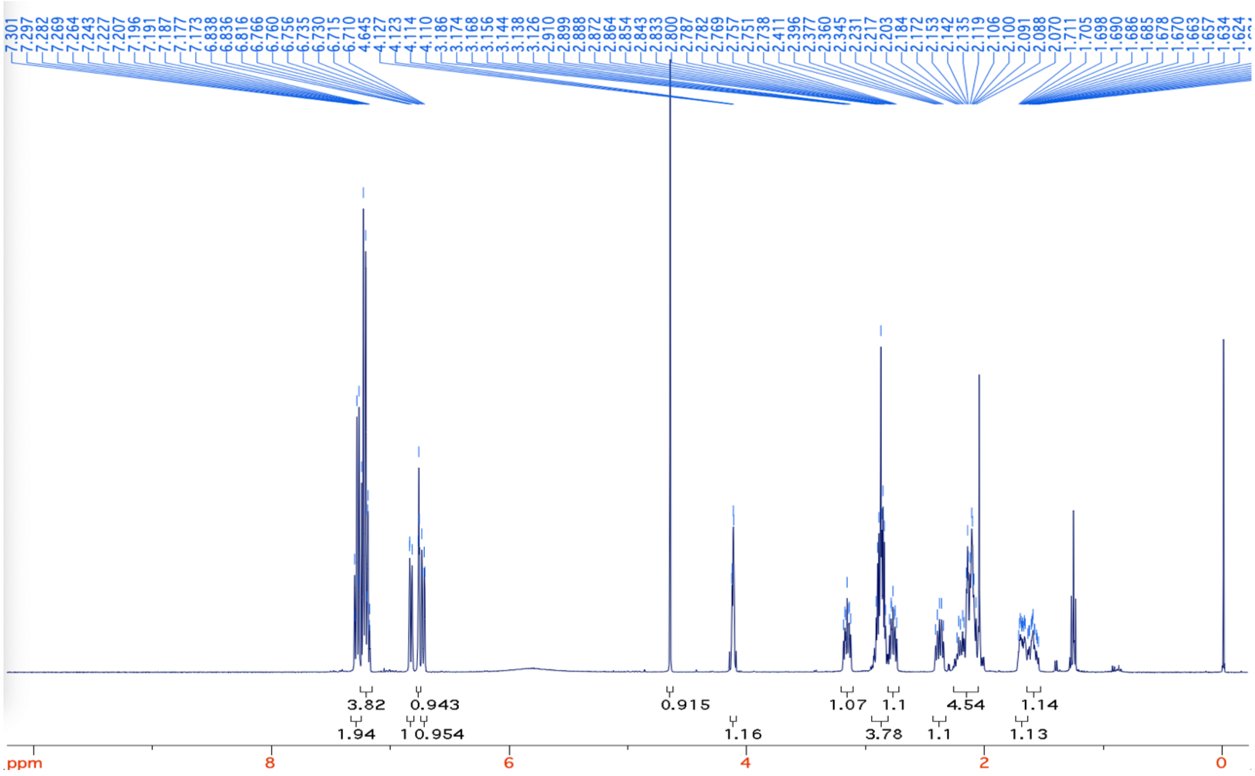

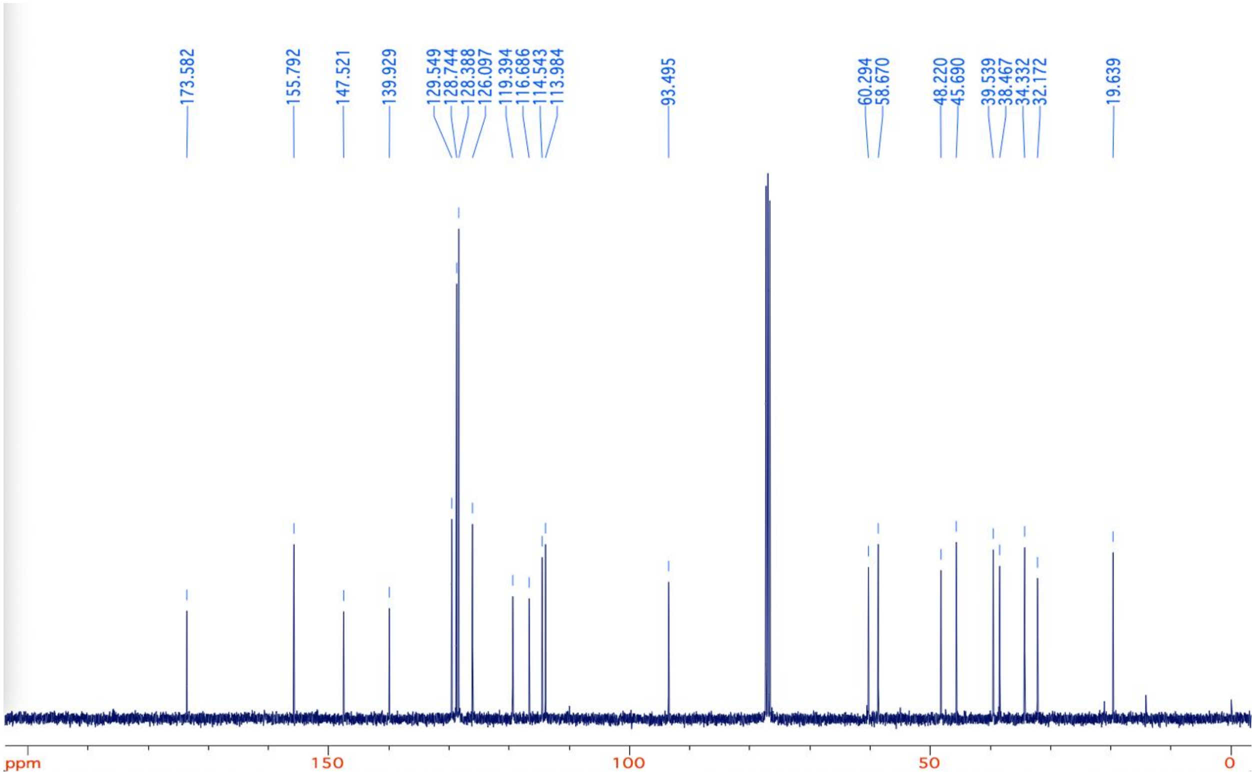

Figure S20. ^{1}H NMR and ^{13}C NMR of 1*R*,5*R*-**33**

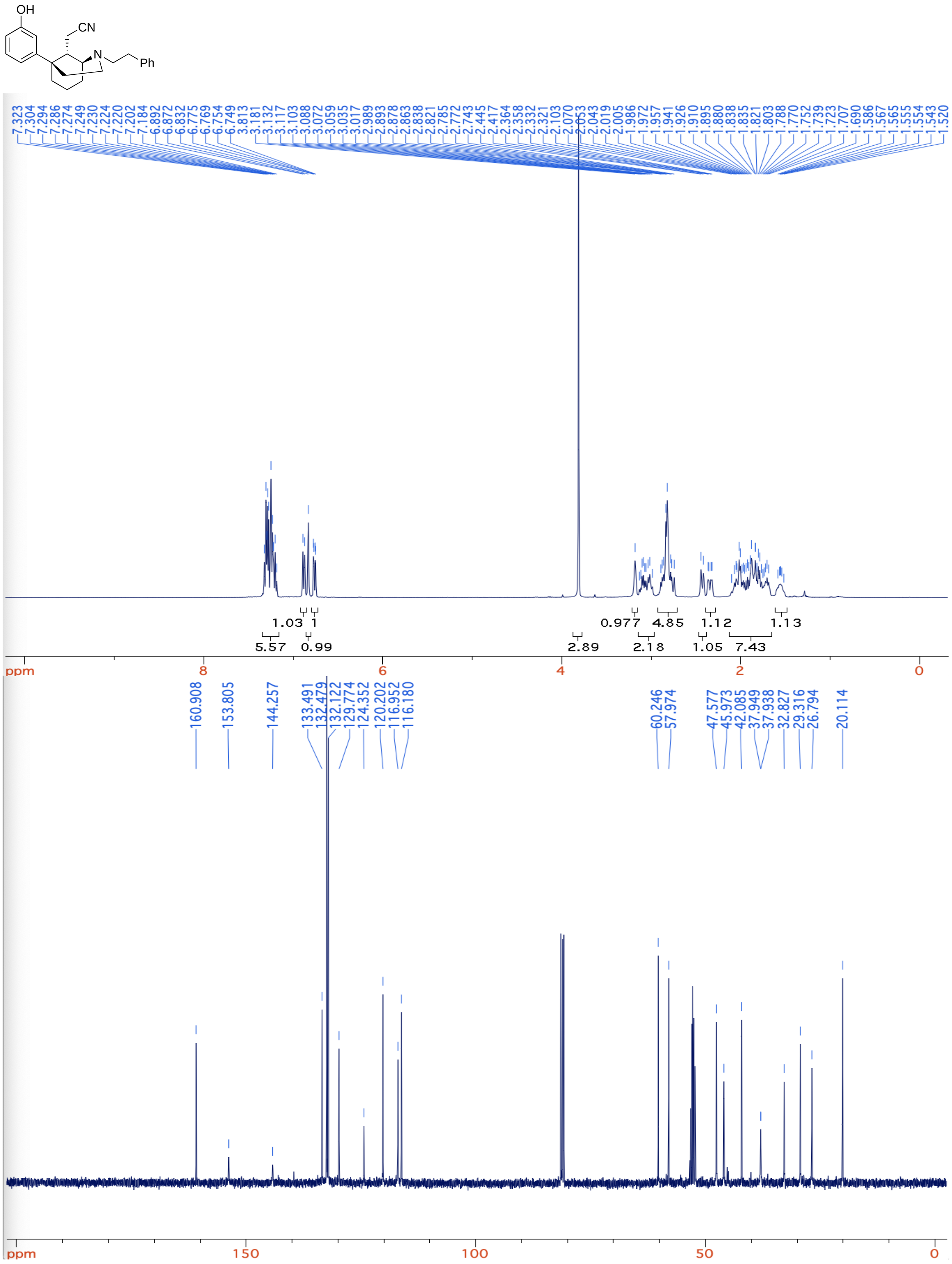

Figure S21. ^{1}H NMR and ^{13}C NMR of 1*S*,5*R*-9*S*-**34**

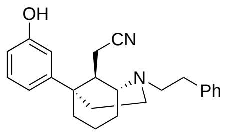

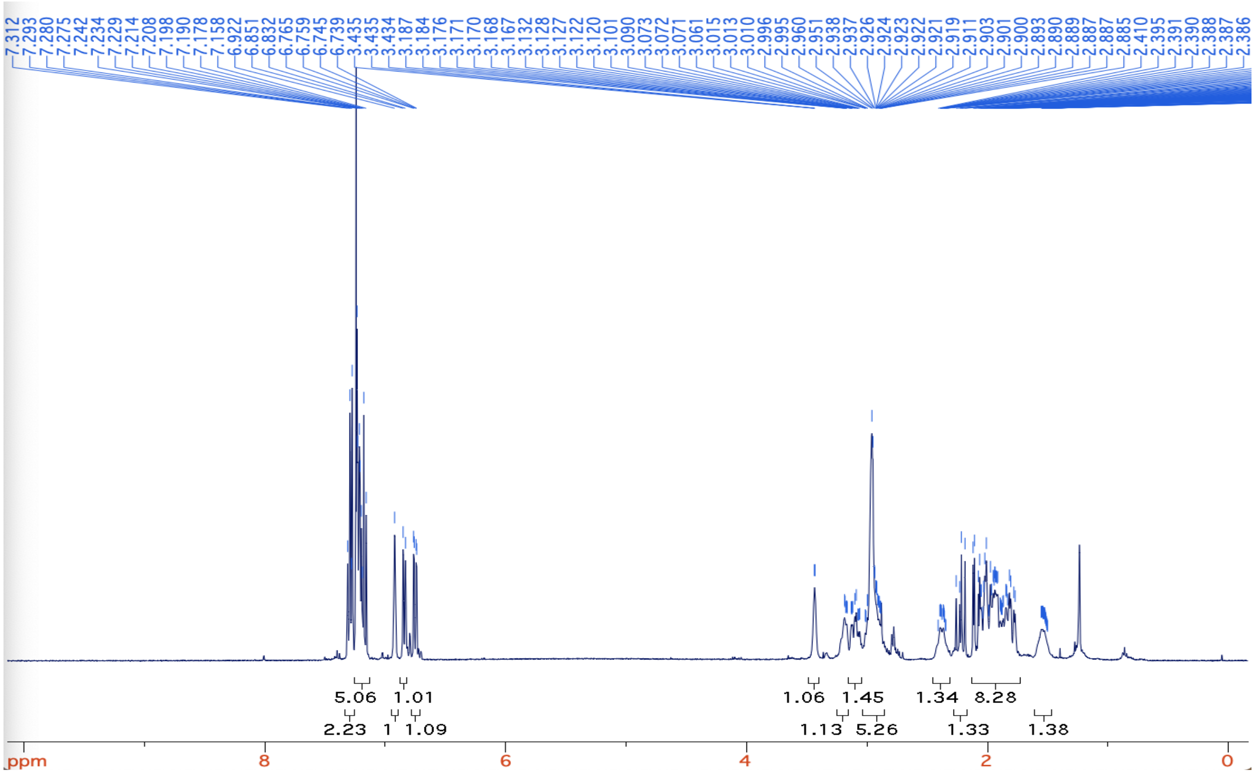

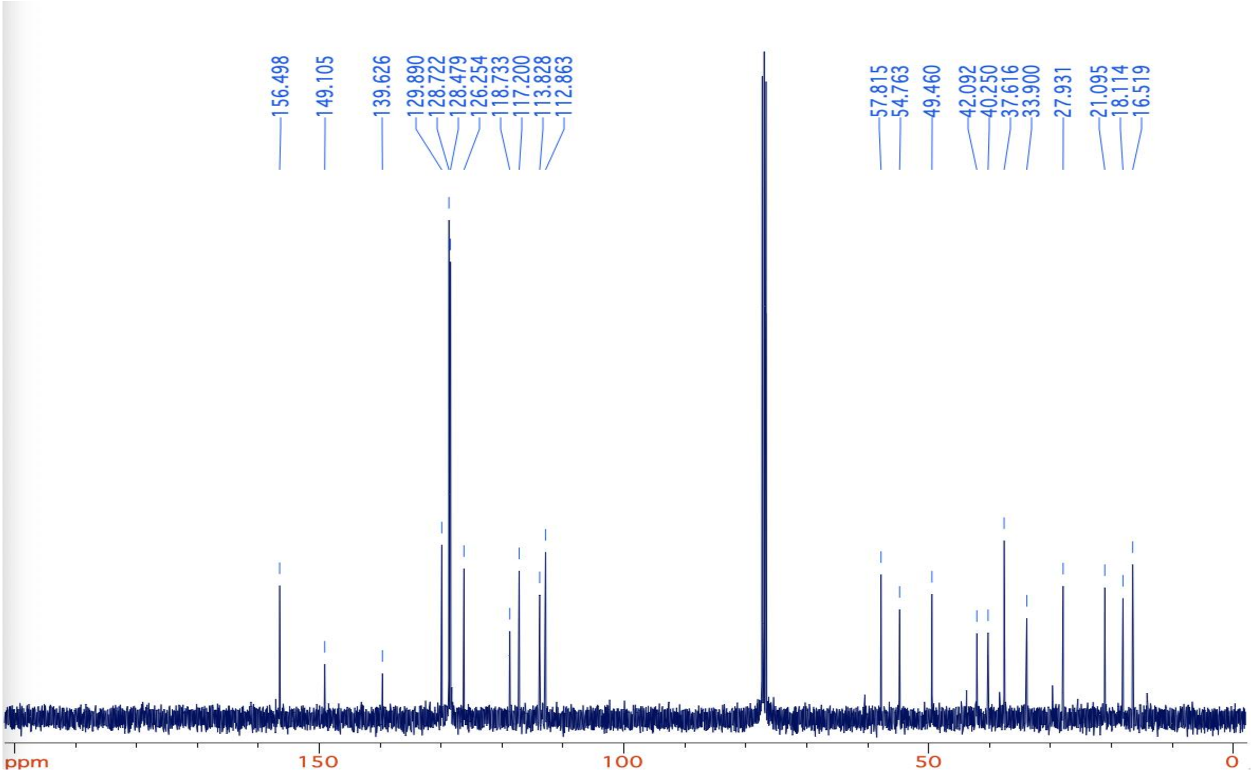

Figure S22. ^{1}H NMR and ^{13}C NMR of 1*R*,5*S*-9*R*-**35**

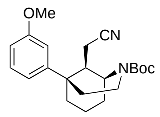

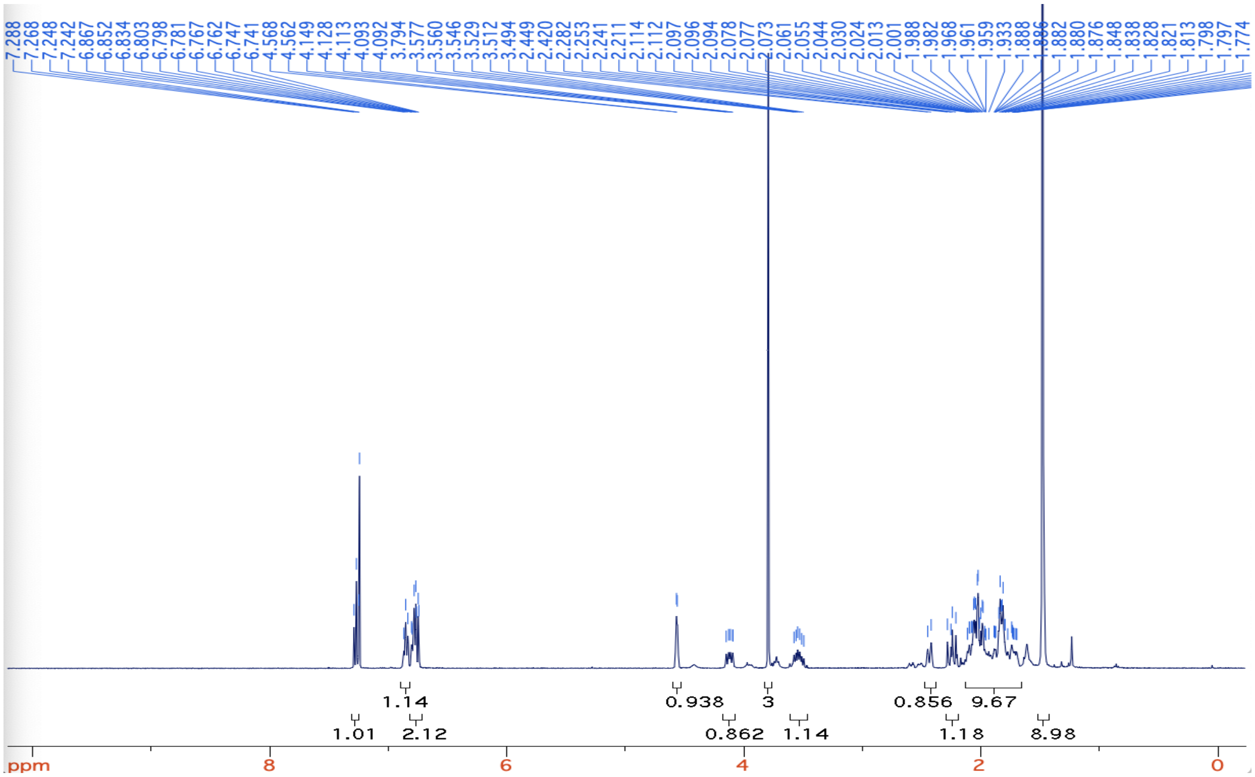

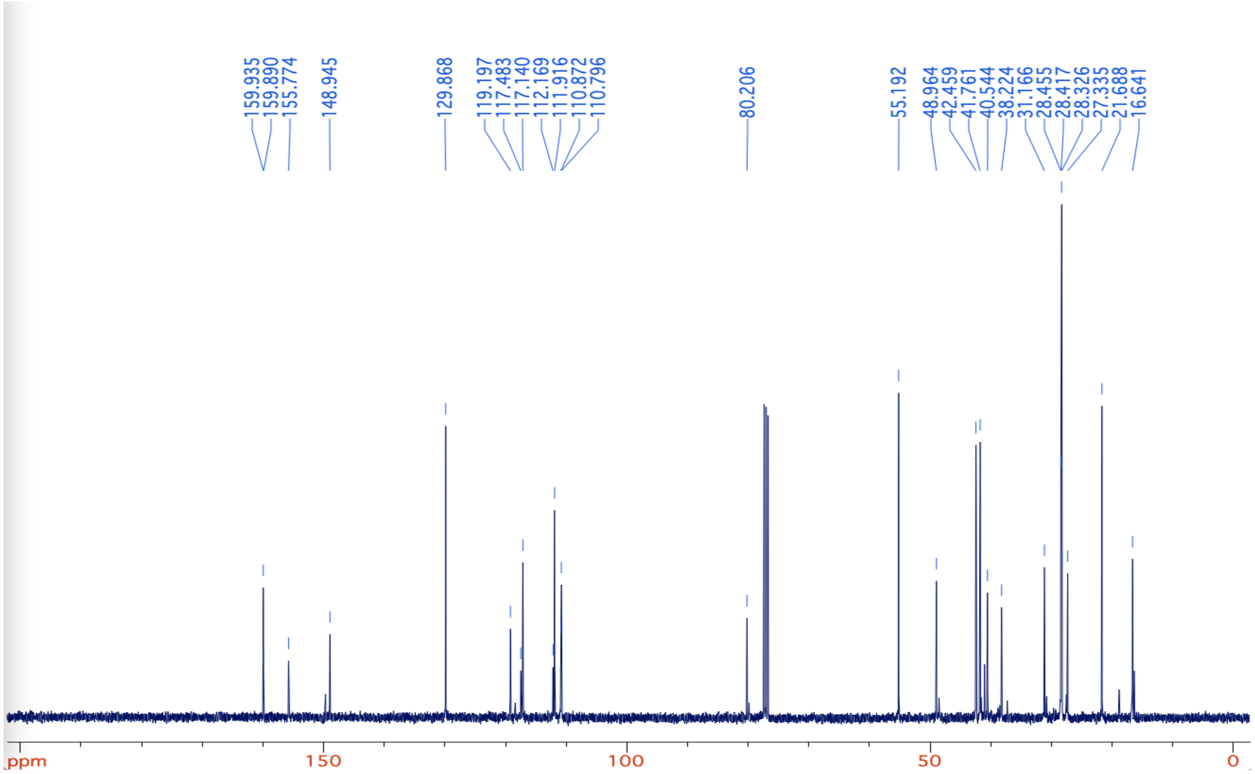

Figure S23. ^{1}H NMR and ^{13}C NMR of 1*S*,5*R*-9*R*-**36**

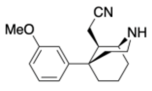

and

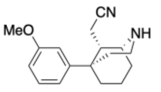

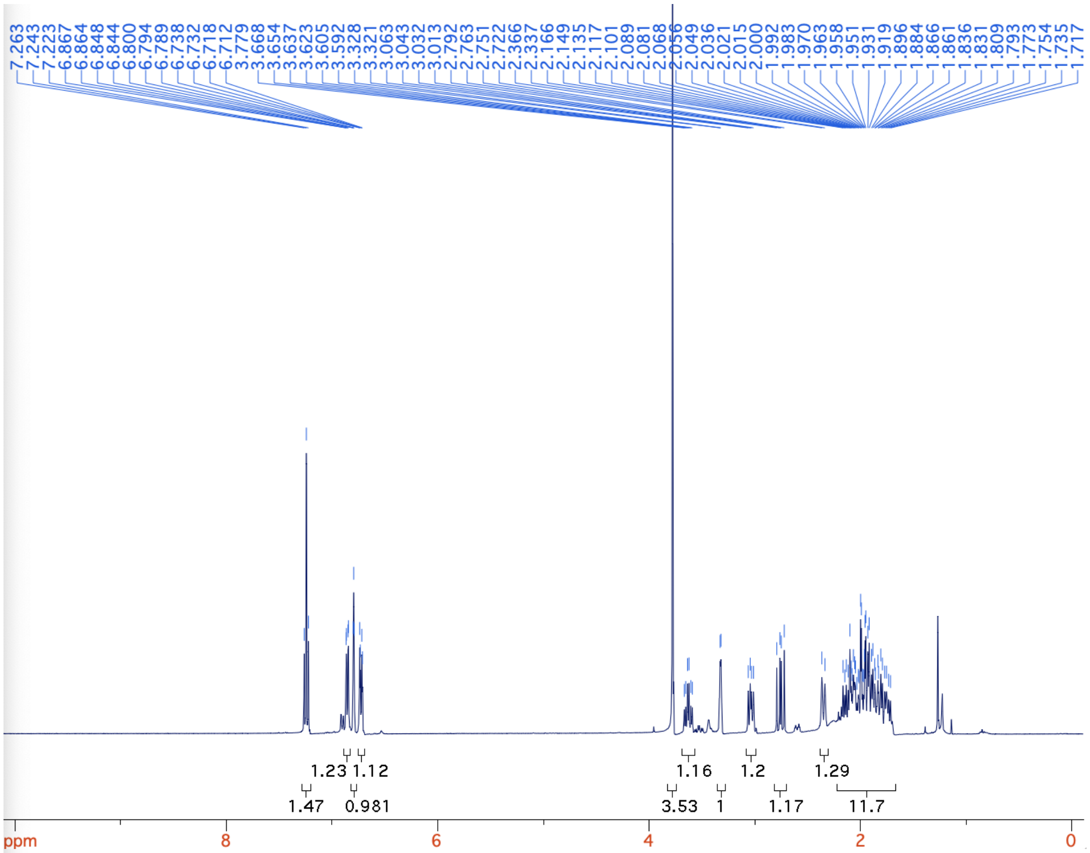

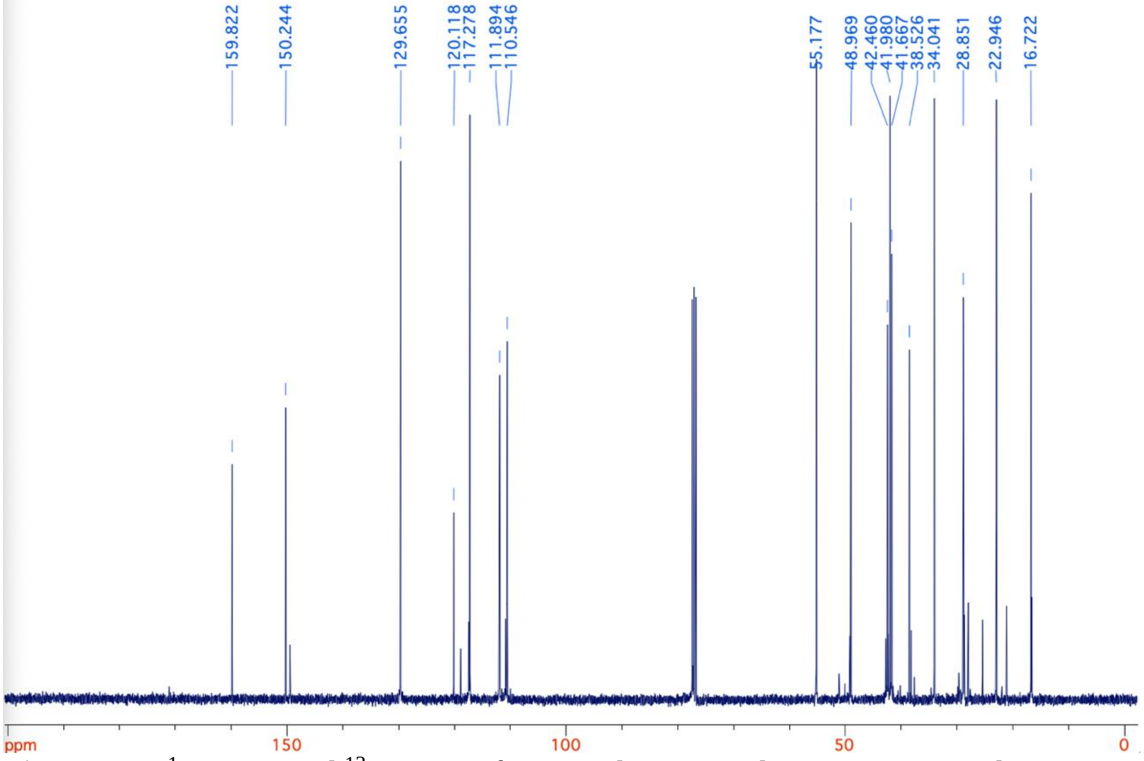

Figure S24. ^{1}H NMR and ^{13}C NMR of intermediate secondary amines towards 1*S*,5*R*,9*R*-**37** and 1*R*,5*S*,9*S*-**40**

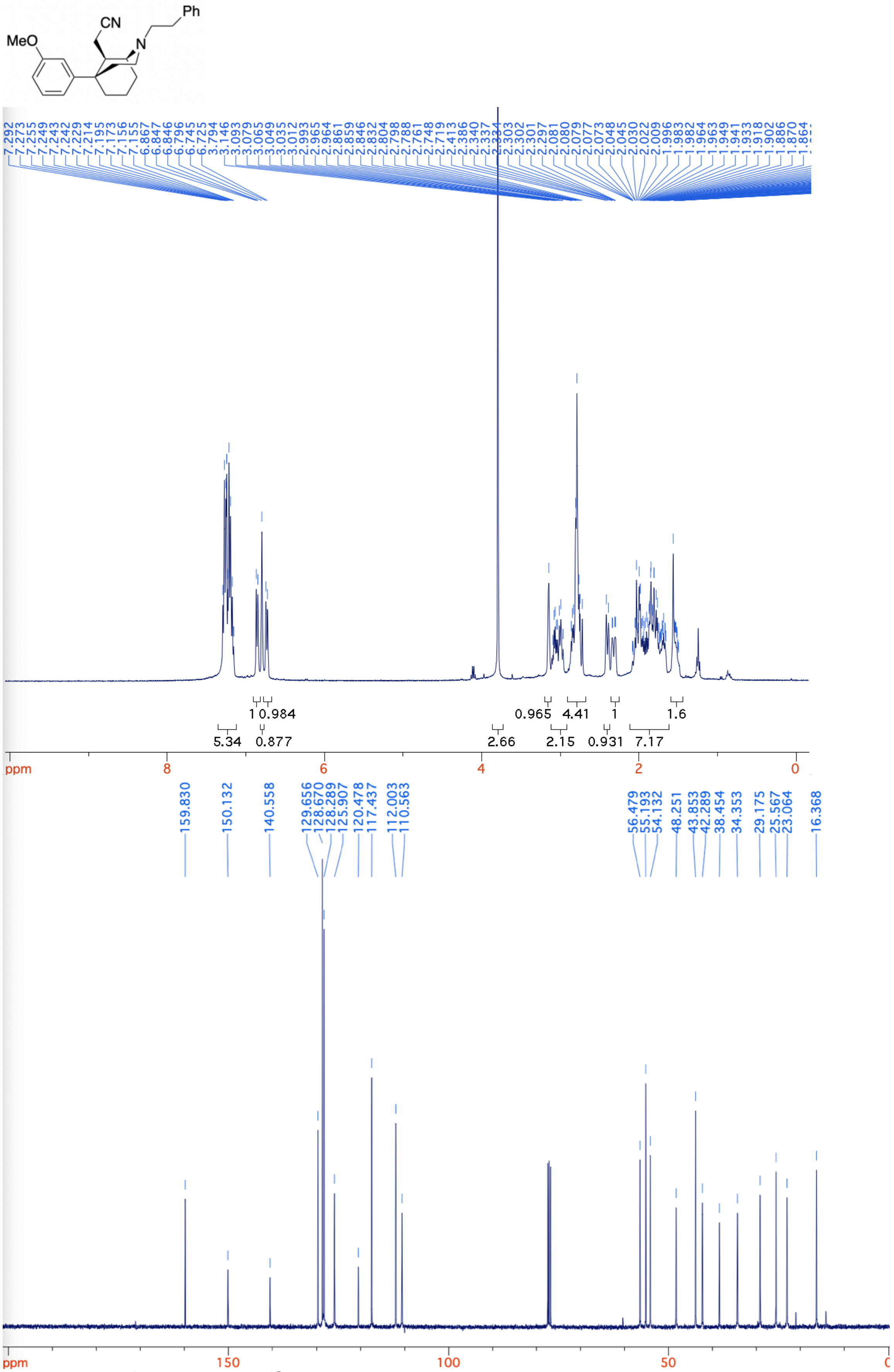

Figure S25. ^{1}H NMR and ^{13}C NMR of 1*S*,5*R*,9*R*-**37**

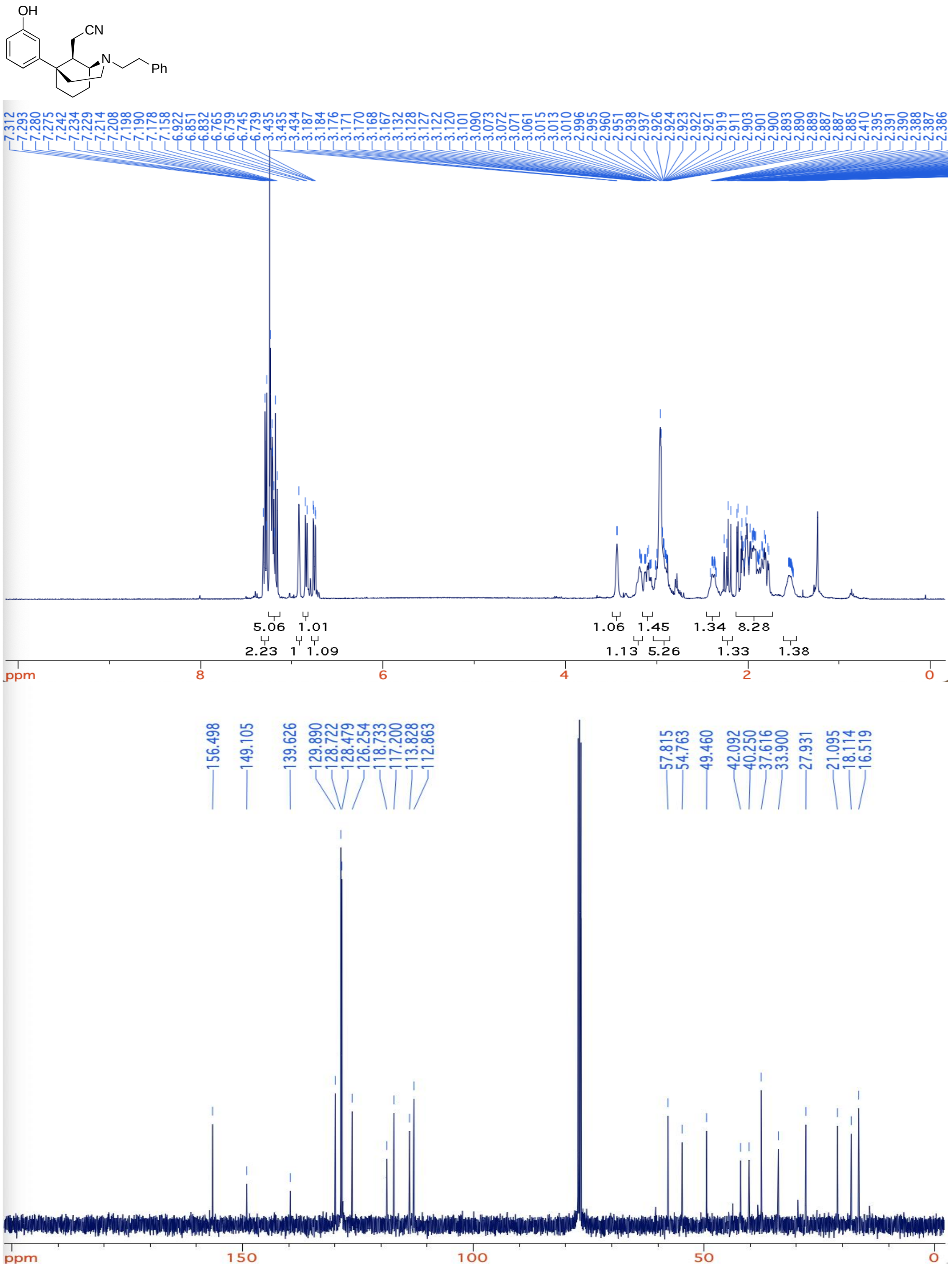

Figure S26. ^{1}H NMR and ^{13}C NMR of 1*S*,5*R*-9*R*-**38**

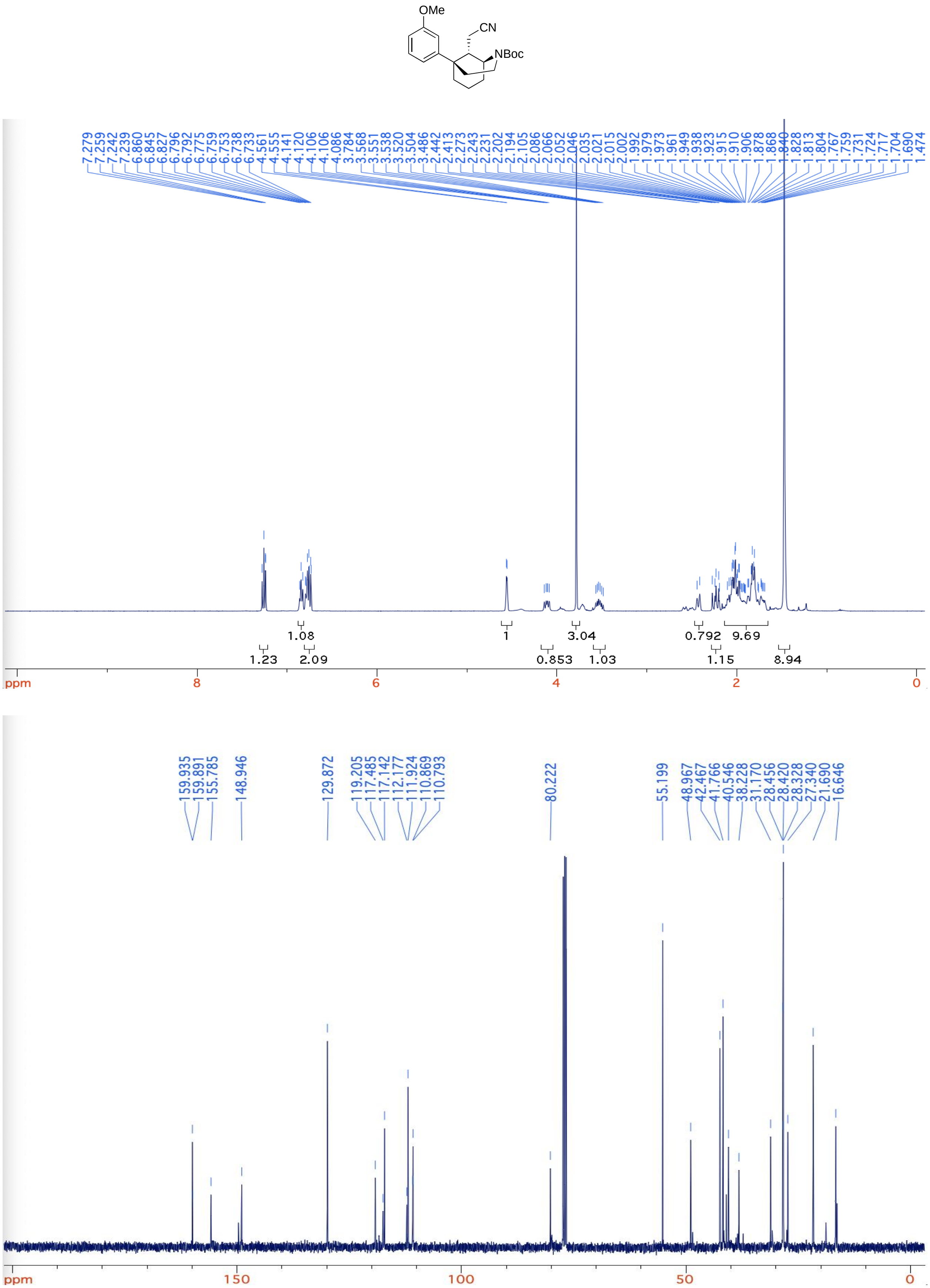

Figure S27. ^{1}H NMR and ^{13}C NMR of 1*R*,5*S*-9*S*-**39**

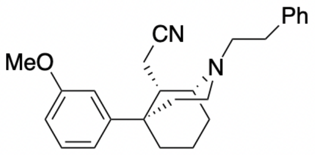

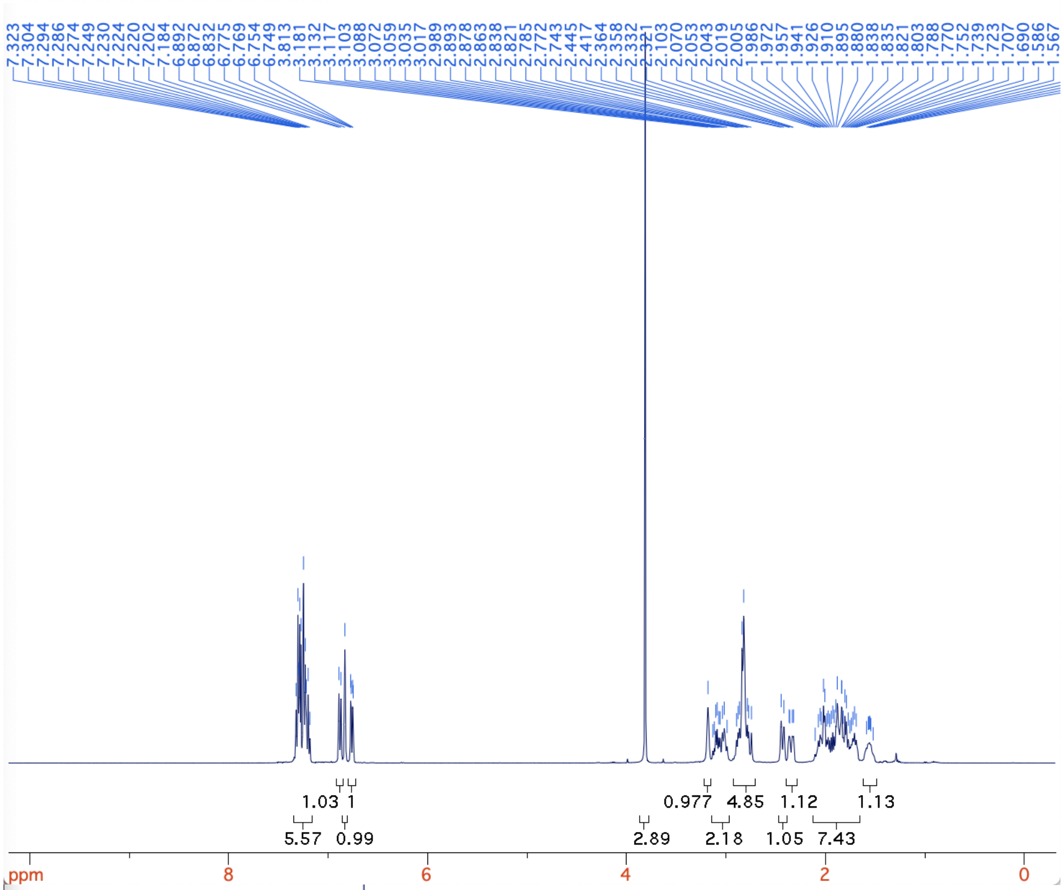

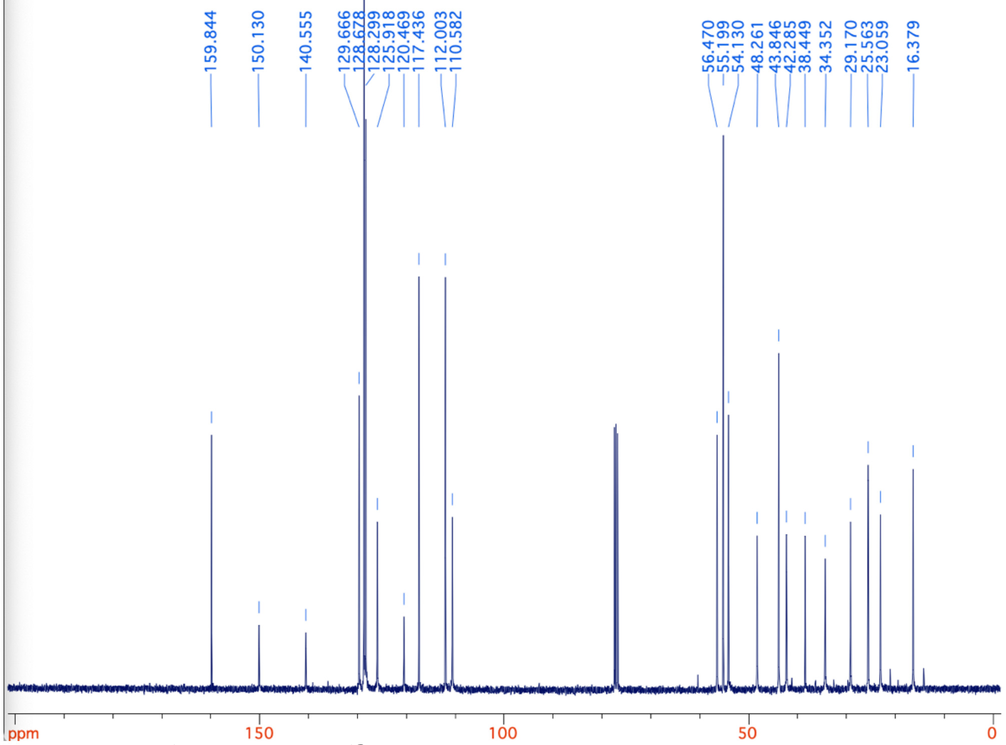

Figure S28. ^{1}H NMR and ^{13}C NMR of 1*R*,5*S*,9*S*-**40**

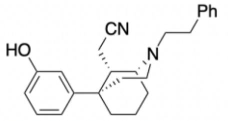

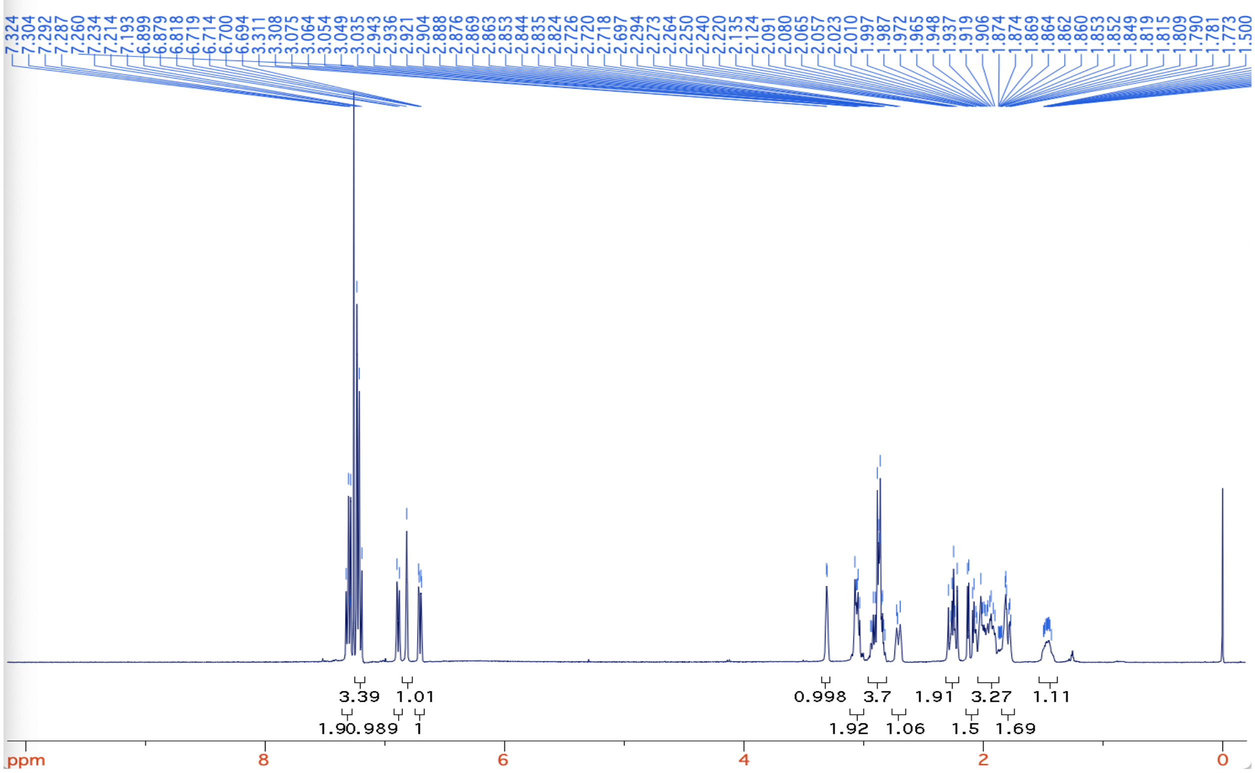

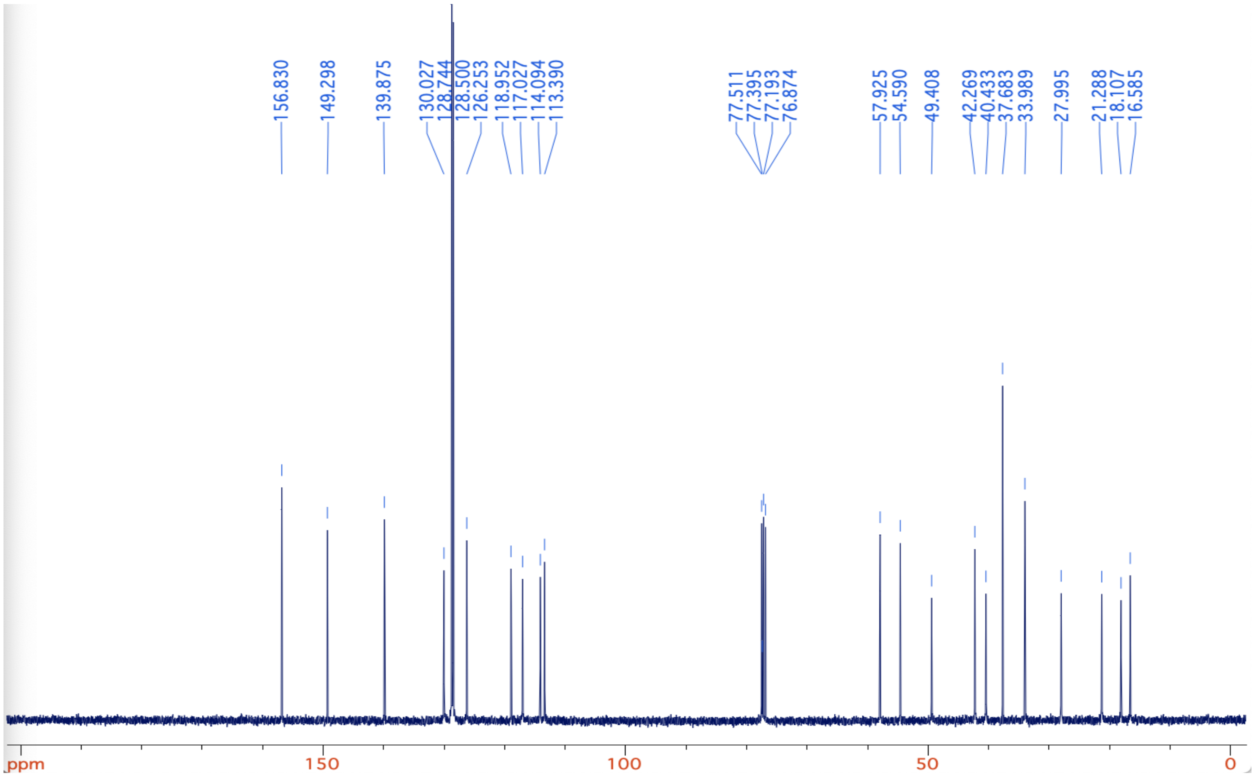

Figure S29. ^{1}H NMR and ^{13}C NMR of 1*R*,5*S*-9*S*-**41**

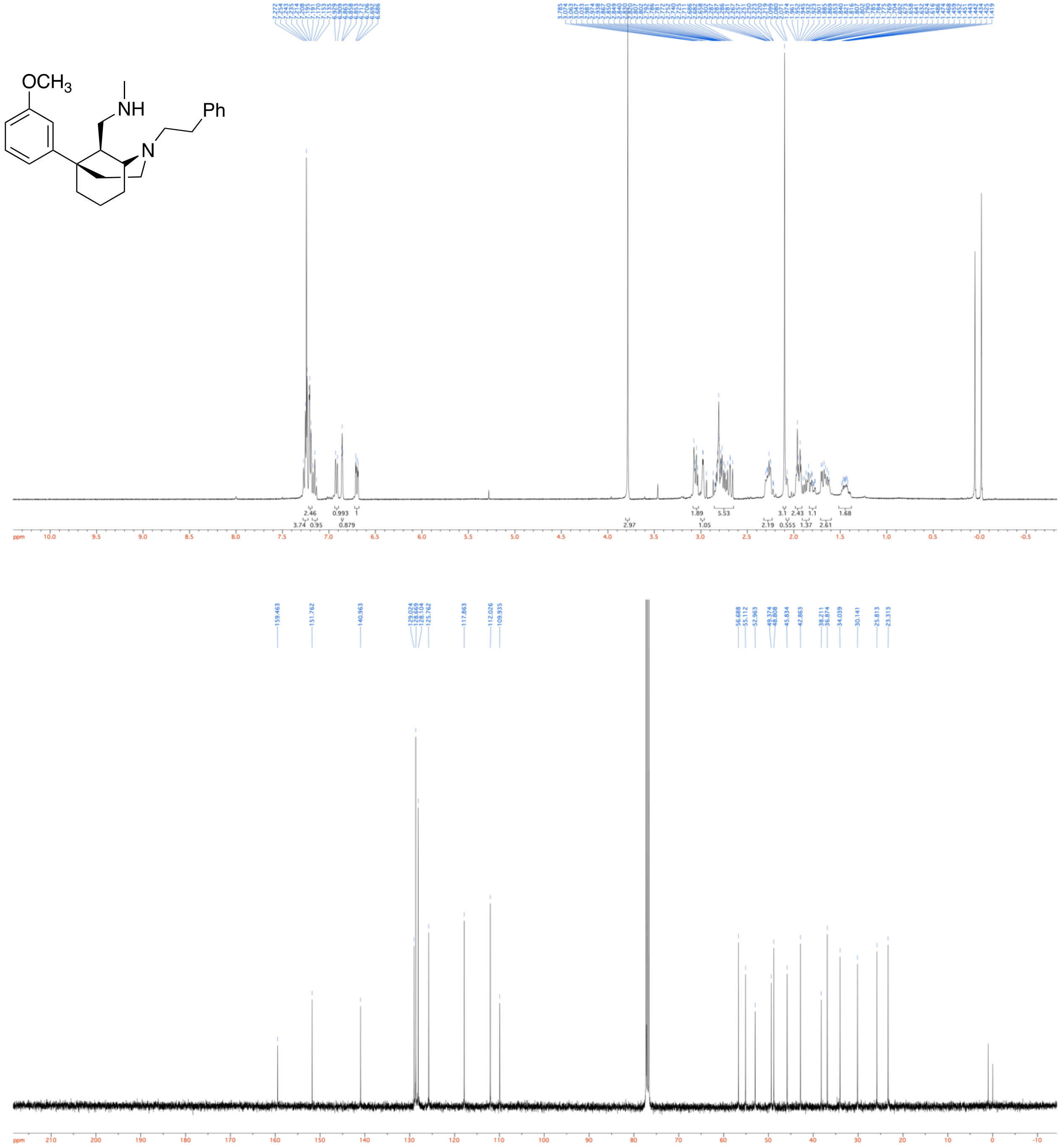

Figure S30. ^{1}H NMR and ^{13}C NMR of 1*S*,5*R*,9*S*-**44**

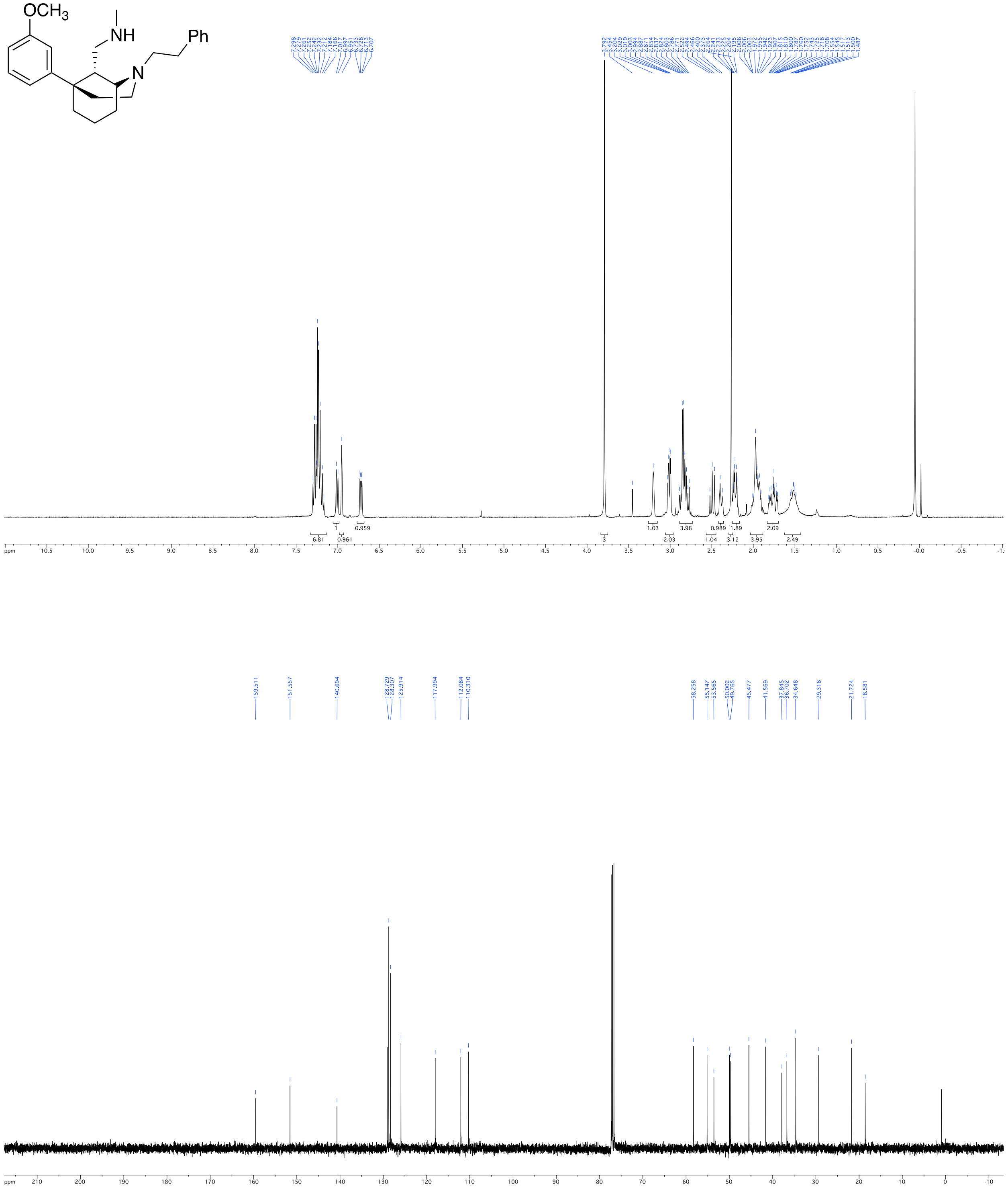

Figure S31. ^{1}H NMR and ^{13}C NMR of 1*S*,5*R*,9*R*-**45**

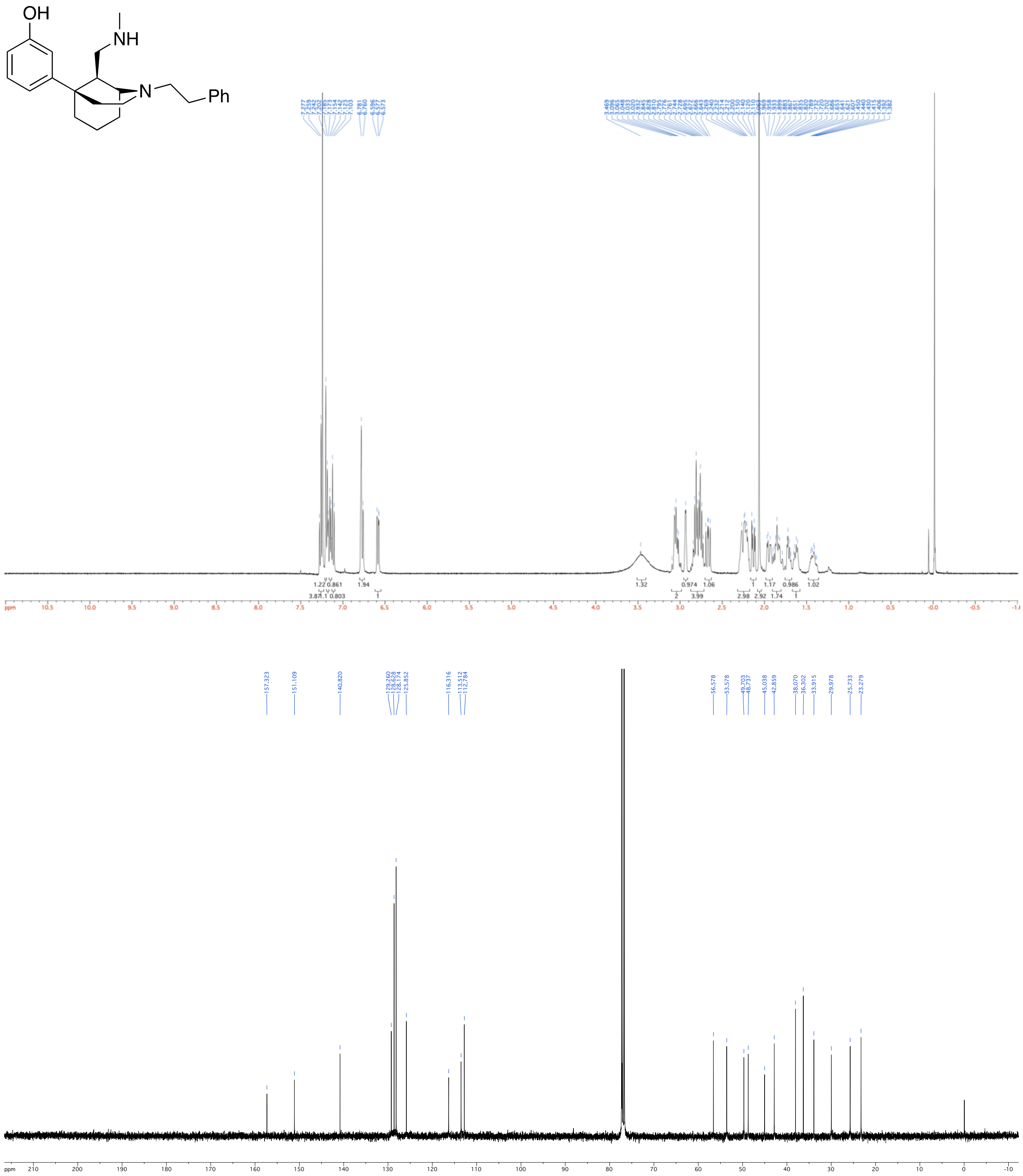

Figure S32. ^{1}H NMR and ^{13}C NMR of 1*S*,5*R*,9*S*-**46**

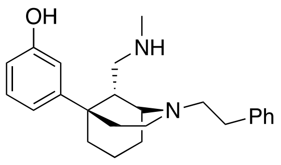

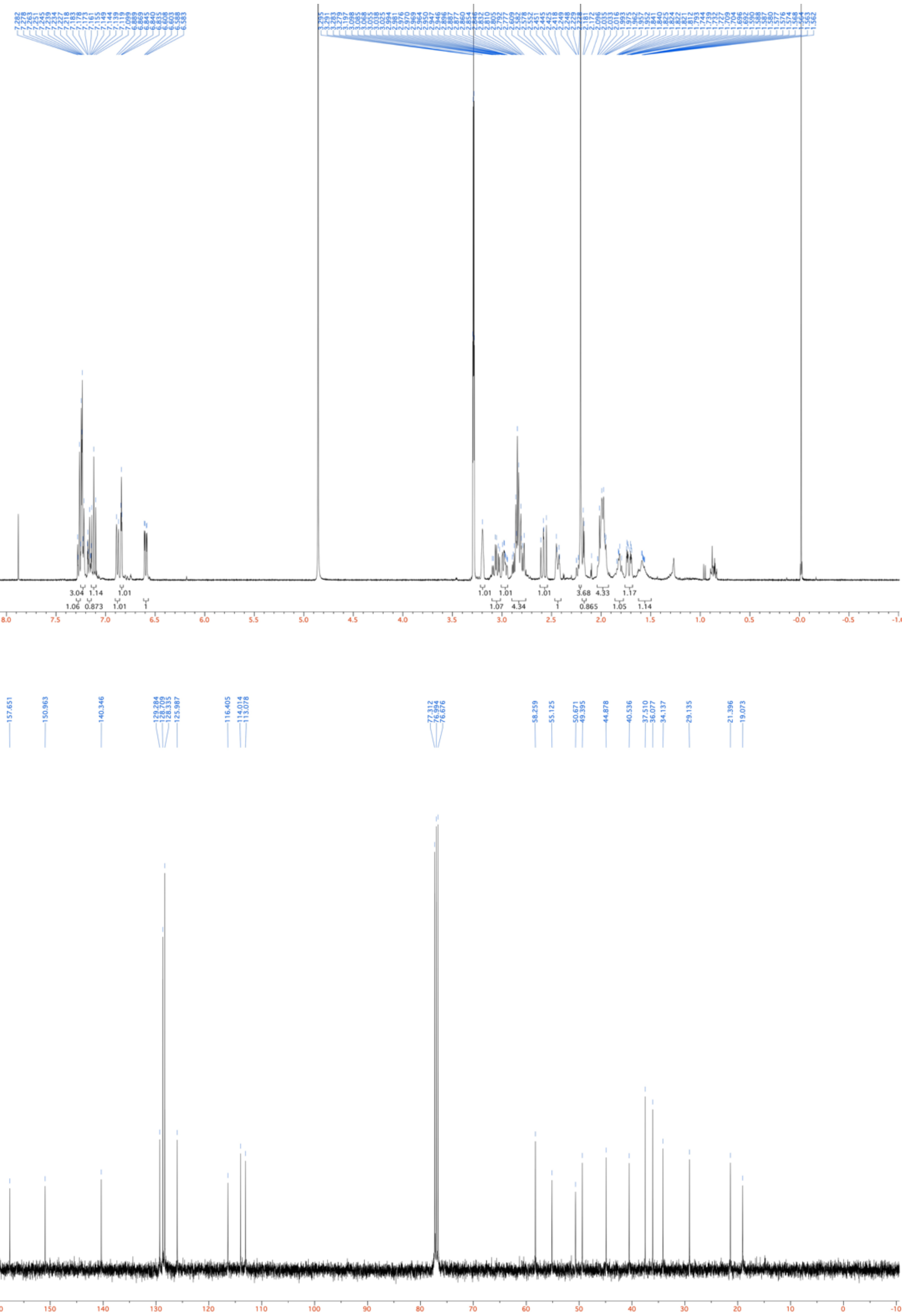

Figure S33. ^{1}H NMR and ^{13}C NMR of 1*S*,5*R*,9*R*-**47**

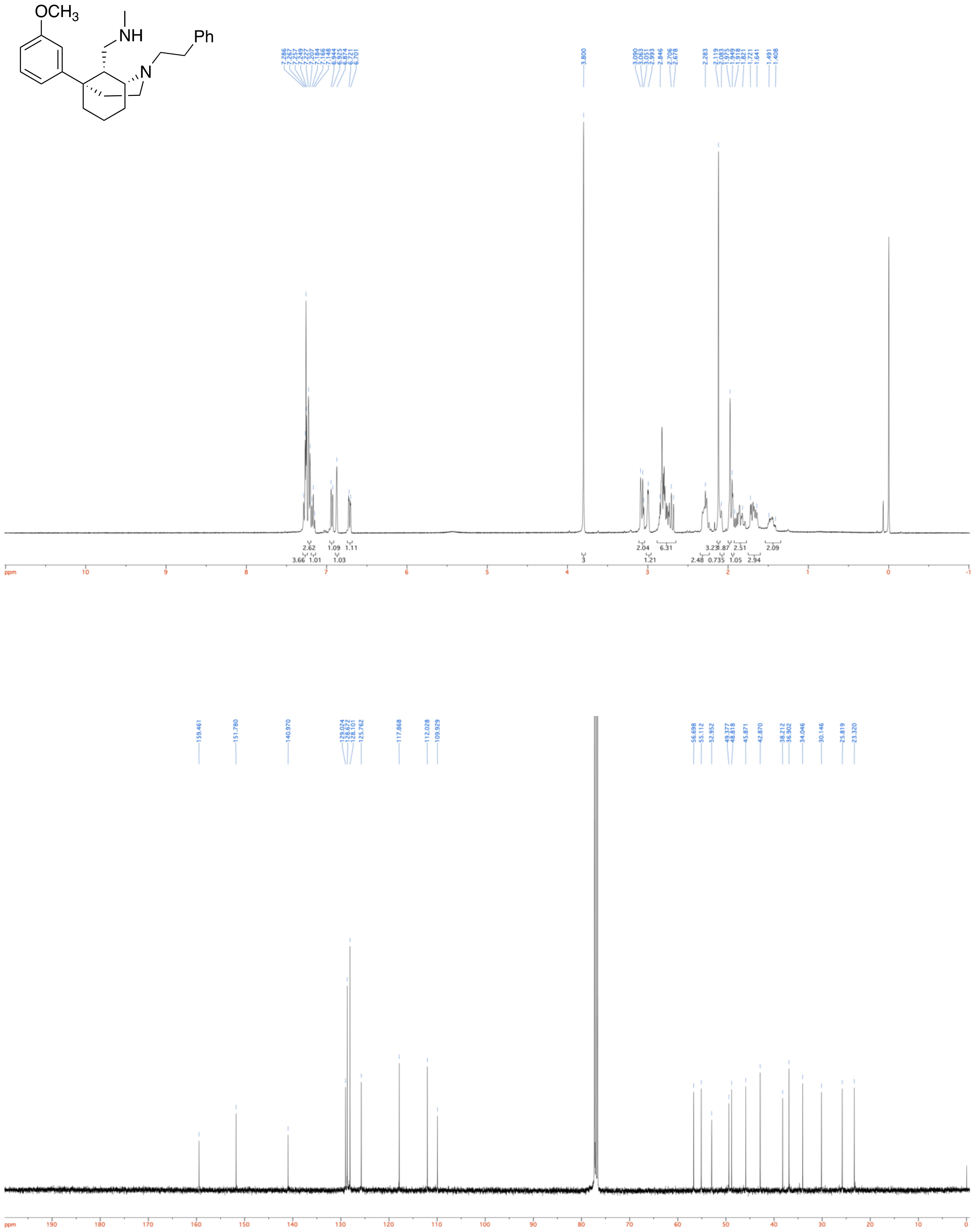

Figure S34. ^{1}H NMR and ^{13}C NMR of 1*R*,5*S*,9*R*-**50**

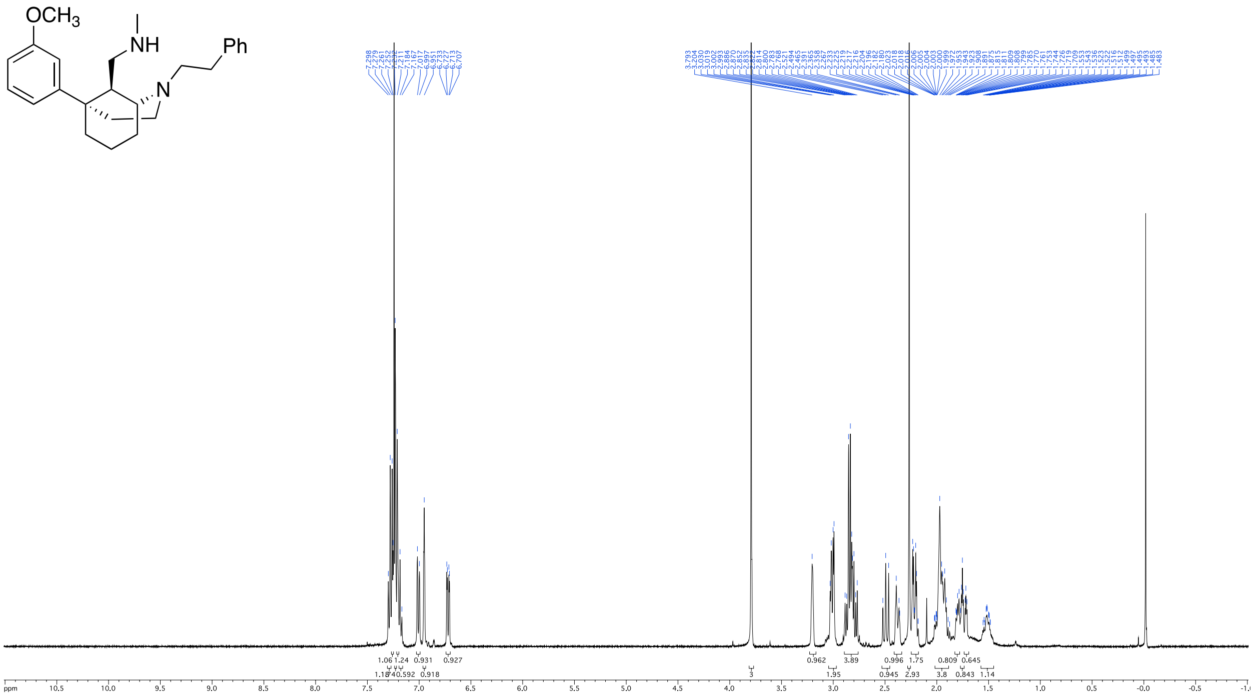

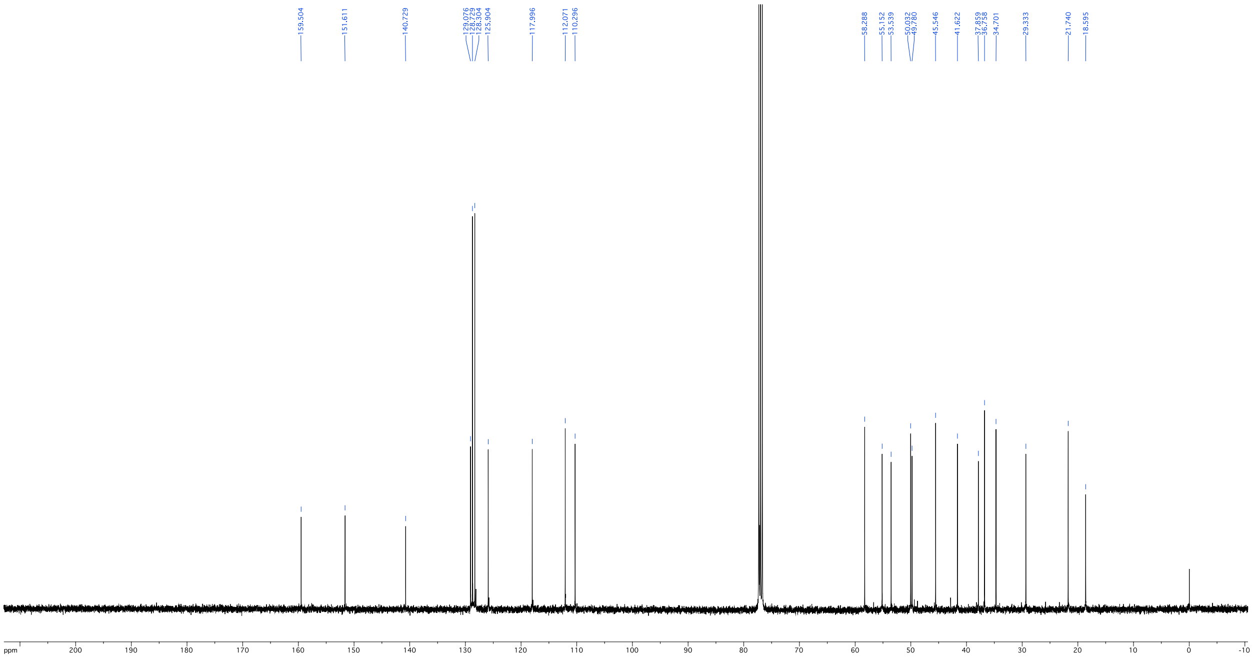

Figure S35. ^{1}H NMR and ^{13}C NMR of 1*R*,5*S*,9*S*-**51**

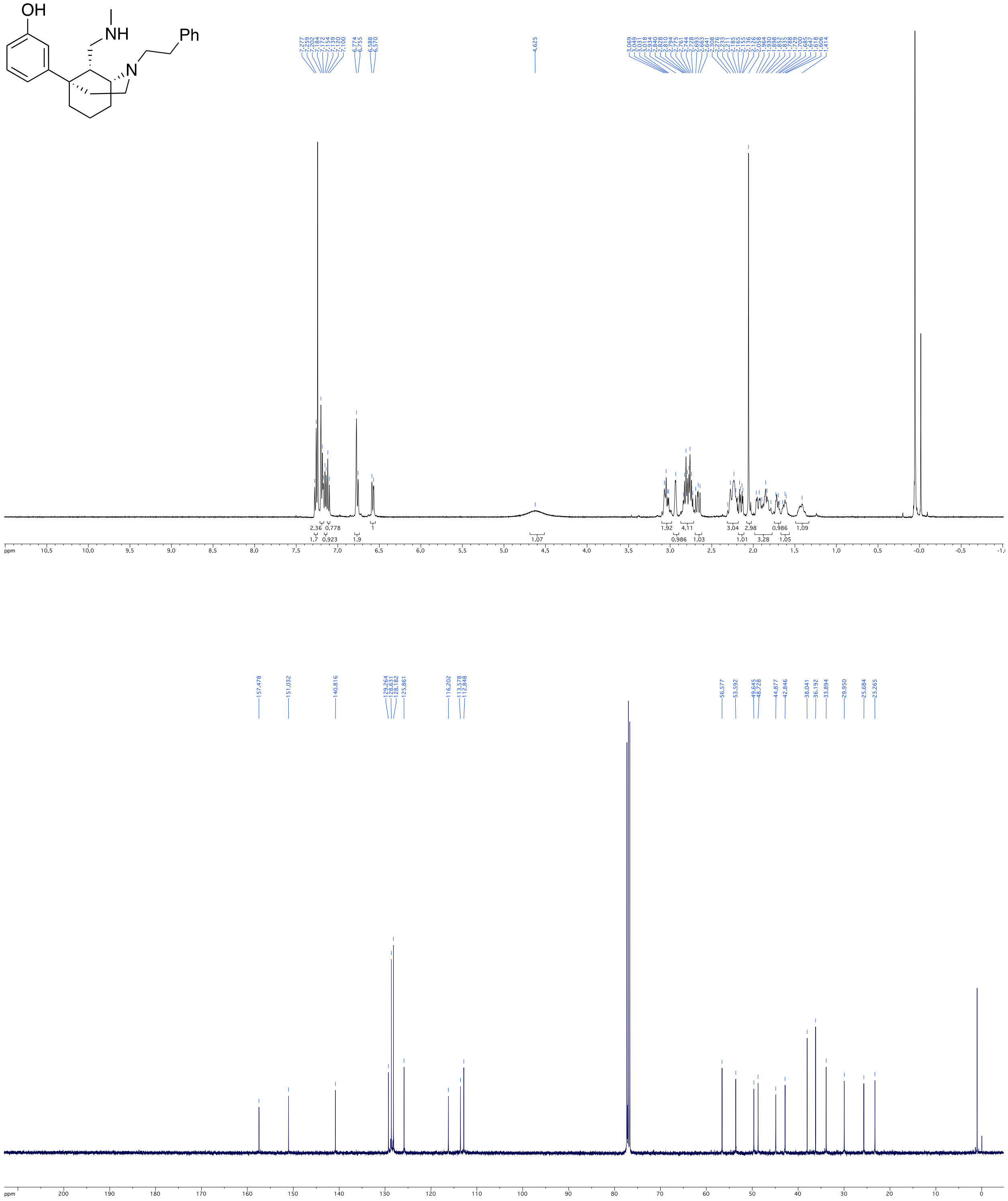

Figure S36. ^{1}H NMR and ^{13}C NMR of 1*R*,5*S*,9*R*-**52**

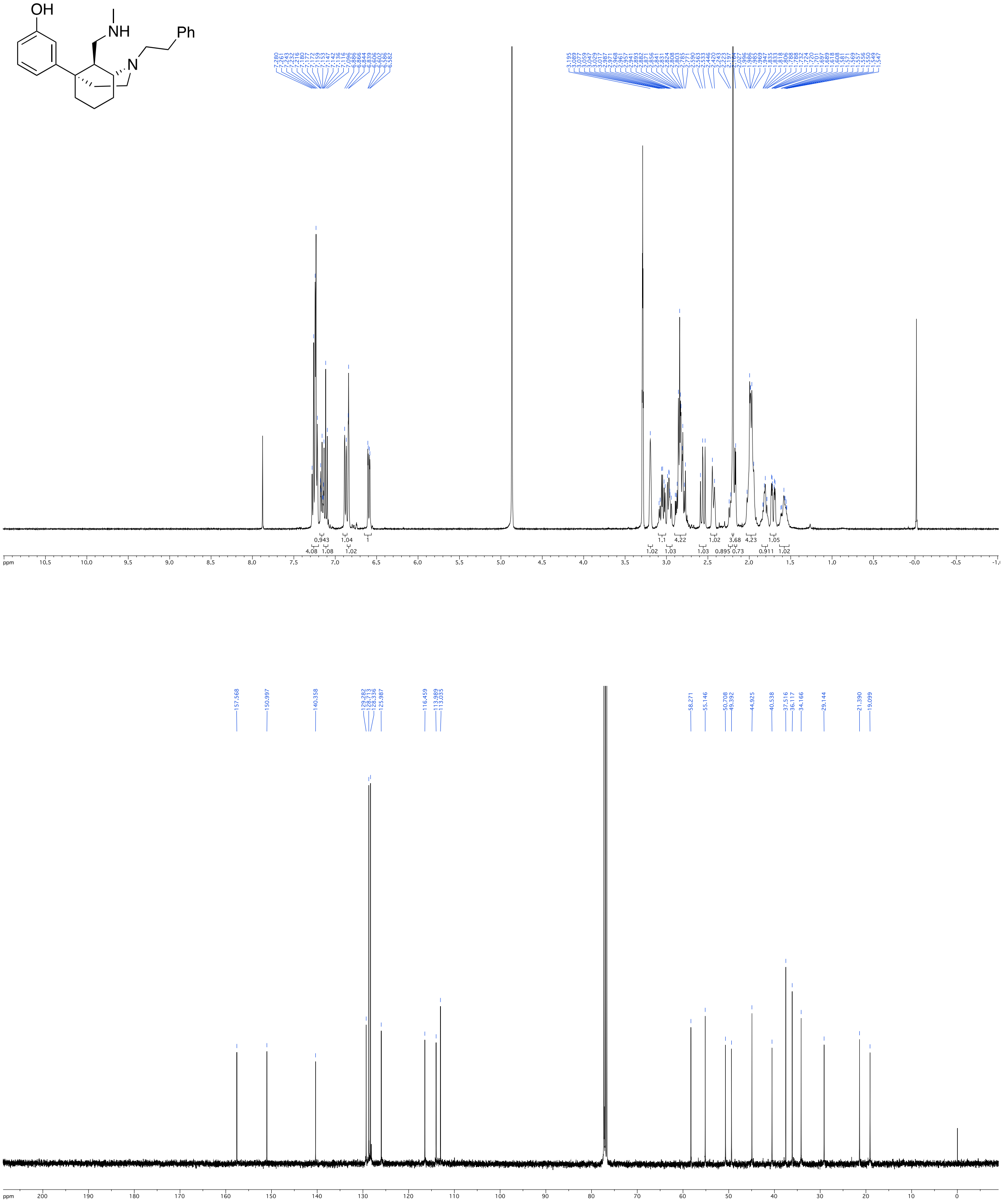

Figure S37. ^{1}H NMR and ^{13}C NMR of *R*,5*S*,9*S*-**53**

Table S1. Crystal data and structure refinement for **23**·2HCl·MeOH·H_2O.

Identification code	Compound_**23**	
Empirical formula	C23 H36 Cl2 N2 O3	
Formula weight	459.44	
Temperature	273(2) K	
Wavelength	1.54178 Å	
Crystal system	Orthorhombic	
Space group	P 21 21 21	
Unit cell dimensions	a = 7.0129(2) Å	α= 90°.
	b = 13.7614(3) Å	β= 90°.
	c = 26.4132(6) Å	γ = 90°.
Volume	2549.07(11) Å^3	
Collected on	Cu Kα Bruker D8 Quest SMART APEX II CCD	
Z	4	
Density (calculated)	1.197 Mg/m^3	
Absorption coefficient	2.484 mm^{-1}	
F(000)	984	
Crystal size	0.341 x 0.046 x 0.030 mm^3	
Theta range for data collection	3.346 to 74.641°.	
Index ranges	-8<=h<=8, -17<=k<=17, -32<=l<=33	
Reflections collected	37321	
Independent reflections	5219 [R(int) = 0.0402]	
Completeness to theta = 67.679°	100.0 %	
Absorption correction	Semi-empirical from equivalents	
Max. and min. transmission	0.7538 and 0.6463	
Refinement method	Full-matrix least-squares on F^2	
Data / restraints / parameters	5219 / 20 / 296	
Goodness-of-fit on F^2	1.113	
Final R indices [I>2sigma(I)]	R1 = 0.0474, wR2 = 0.1430	
R indices (all data)	R1 = 0.0489, wR2 = 0.1445	
Absolute structure parameter	0.032(4)	
Extinction coefficient	0.0018(5)	
Largest diff. peak and hole	1.374 and -0.263 e.Å^{-3}	

Table S2. Atomic coordinates (x 10^4) and equivalent isotropic displacement parameters ($Å^2$x 10^3) for **23**·2HCl·MeOH·H_2O. U(eq) is defined as one third of the trace of the orthogonalized U^{ij} tensor.

	x	y	z	U(eq)
C(12)	766(6)	8909(3)	7192(1)	32(1)
C(13)	1252(6)	9876(3)	7137(2)	36(1)
C(14)	2951(7)	10105(3)	6895(2)	38(1)
C(15)	4157(6)	9397(3)	6717(1)	32(1)
C(10)	3671(5)	8416(3)	6762(1)	25(1)
C(11)	1932(5)	8188(3)	6995(1)	26(1)
C(5)	4972(5)	7598(3)	6568(1)	24(1)
C(6)	6877(5)	7990(3)	6360(1)	27(1)
C(7)	8172(5)	7210(3)	6129(2)	31(1)
C(1)	5158(5)	6171(3)	5971(1)	22(1)
C(9)	3936(5)	7033(2)	6146(1)	20(1)
C(4)	5290(5)	6868(3)	7012(1)	28(1)
C(3)	6101(5)	5871(3)	6875(1)	28(1)
C(16)	6044(6)	4505(3)	6261(2)	30(1)
C(17)	5685(7)	3721(3)	6656(2)	46(1)
C(18)	6348(7)	2750(3)	6446(2)	37(1)
C(23)	5148(11)	2193(4)	6157(3)	67(2)
C(22)	5724(16)	1341(5)	5953(3)	89(3)
C(21)	7515(18)	1021(5)	6029(3)	92(3)
C(20)	8797(12)	1568(6)	6315(3)	88(3)
C(19)	8182(8)	2450(4)	6540(2)	55(1)
Cl(1)	1629(1)	6182(1)	4949(1)	27(1)
Cl(2)	1054(1)	5098(1)	6676(1)	42(1)
N(1)	3495(4)	7659(2)	5698(1)	20(1)
N(2)	5190(4)	5467(2)	6408(1)	24(1)
O(1)	-855(4)	8625(2)	7443(1)	38(1)
O(2)	877(5)	9151(2)	5536(1)	43(1)
C(8)	7128(5)	6482(3)	5786(1)	28(1)
O(3A)	1668(13)	898(7)	5037(3)	77(2)
C(24A)	3204(19)	397(9)	4951(4)	75(2)
O(3B)	5456(19)	795(8)	4529(4)	74(3)
C(24B)	6820(30)	761(12)	4364(6)	75(3)

Table S3. Bond lengths [Å] and angles [°] for **23**·2HCl·MeOH·H_2O.

C(12)-O(1)	1.372(5)
C(12)-C(13)	1.382(6)
C(12)-C(11)	1.387(5)
C(13)-C(14)	1.388(7)
C(13)-H(13)	0.9300
C(14)-C(15)	1.373(6)
C(14)-H(14)	0.9300
C(15)-C(10)	1.397(6)
C(15)-H(15)	0.9300
C(10)-C(11)	1.402(5)
C(10)-C(5)	1.537(5)
C(11)-H(11)	0.9300
C(5)-C(9)	1.541(4)
C(5)-C(6)	1.543(5)
C(5)-C(4)	1.560(5)
C(6)-C(7)	1.532(6)
C(6)-H(6A)	0.9700
C(6)-H(6B)	0.9700
C(7)-C(8)	1.536(6)
C(7)-H(7A)	0.9700
C(7)-H(7B)	0.9700
C(1)-N(2)	1.507(4)
C(1)-C(8)	1.526(5)
C(1)-C(9)	1.535(5)
C(1)-H(1)	0.9800
C(9)-N(1)	1.496(4)
C(9)-H(9)	0.9800
C(4)-C(3)	1.528(6)
C(4)-H(4A)	0.9700
C(4)-H(4B)	0.9700
C(3)-N(2)	1.496(5)
C(3)-H(3A)	0.9700
C(3)-H(3B)	0.9700
C(16)-N(2)	1.503(5)
C(16)-C(17)	1.522(5)
C(16)-H(16A)	0.9700
C(16)-H(16B)	0.9700

C(17)-C(18)	1.520(6)
C(17)-H(17A)	0.9700
C(17)-H(17B)	0.9700
C(18)-C(23)	1.370(8)
C(18)-C(19)	1.374(7)
C(23)-C(22)	1.352(11)
C(23)-H(23)	0.9300
C(22)-C(21)	1.346(14)
C(22)-H(22)	0.9300
C(21)-C(20)	1.394(14)
C(21)-H(21)	0.9300
C(20)-C(19)	1.418(10)
C(20)-H(20)	0.9300
C(19)-H(19)	0.9300
N(1)-H(1A)	0.8900
N(1)-H(1B)	0.8900
N(1)-H(1C)	0.8900
N(2)-H(2)	0.9800
O(1)-H(1D)	0.8200
O(2)-H(2A)	0.8501
O(2)-H(2B)	0.8500
C(8)-H(8A)	0.9700
C(8)-H(8B)	0.9700
O(3A)-C(24A)	1.299(15)
O(3B)-C(24B)	1.055(17)

O(1)-C(12)-C(13)	122.0(4)
O(1)-C(12)-C(11)	117.7(4)
C(13)-C(12)-C(11)	120.3(4)
C(12)-C(13)-C(14)	118.6(4)
C(12)-C(13)-H(13)	120.7
C(14)-C(13)-H(13)	120.7
C(15)-C(14)-C(13)	121.7(4)
C(15)-C(14)-H(14)	119.2
C(13)-C(14)-H(14)	119.2
C(14)-C(15)-C(10)	120.4(4)
C(14)-C(15)-H(15)	119.8
C(10)-C(15)-H(15)	119.8
C(15)-C(10)-C(11)	117.8(3)

C(15)-C(10)-C(5)	122.3(3)
C(11)-C(10)-C(5)	119.9(3)
C(12)-C(11)-C(10)	121.2(4)
C(12)-C(11)-H(11)	119.4
C(10)-C(11)-H(11)	119.4
C(10)-C(5)-C(9)	109.3(3)
C(10)-C(5)-C(6)	112.1(3)
C(9)-C(5)-C(6)	109.0(3)
C(10)-C(5)-C(4)	107.9(3)
C(9)-C(5)-C(4)	106.6(3)
C(6)-C(5)-C(4)	111.7(3)
C(7)-C(6)-C(5)	114.2(3)
C(7)-C(6)-H(6A)	108.7
C(5)-C(6)-H(6A)	108.7
C(7)-C(6)-H(6B)	108.7
C(5)-C(6)-H(6B)	108.7
H(6A)-C(6)-H(6B)	107.6
C(6)-C(7)-C(8)	114.1(3)
C(6)-C(7)-H(7A)	108.7
C(8)-C(7)-H(7A)	108.7
C(6)-C(7)-H(7B)	108.7
C(8)-C(7)-H(7B)	108.7
H(7A)-C(7)-H(7B)	107.6
N(2)-C(1)-C(8)	114.3(3)
N(2)-C(1)-C(9)	105.9(3)
C(8)-C(1)-C(9)	112.6(3)
N(2)-C(1)-H(1)	107.9
C(8)-C(1)-H(1)	107.9
C(9)-C(1)-H(1)	107.9
N(1)-C(9)-C(1)	108.8(3)
N(1)-C(9)-C(5)	112.3(3)
C(1)-C(9)-C(5)	110.2(3)
N(1)-C(9)-H(9)	108.5
C(1)-C(9)-H(9)	108.5
C(5)-C(9)-H(9)	108.5
C(3)-C(4)-C(5)	117.0(3)
C(3)-C(4)-H(4A)	108.1
C(5)-C(4)-H(4A)	108.1
C(3)-C(4)-H(4B)	108.1

C(5)-C(4)-H(4B)	108.1
H(4A)-C(4)-H(4B)	107.3
N(2)-C(3)-C(4)	111.7(3)
N(2)-C(3)-H(3A)	109.3
C(4)-C(3)-H(3A)	109.3
N(2)-C(3)-H(3B)	109.3
C(4)-C(3)-H(3B)	109.3
H(3A)-C(3)-H(3B)	107.9
N(2)-C(16)-C(17)	112.4(3)
N(2)-C(16)-H(16A)	109.1
C(17)-C(16)-H(16A)	109.1
N(2)-C(16)-H(16B)	109.1
C(17)-C(16)-H(16B)	109.1
H(16A)-C(16)-H(16B)	107.9
C(18)-C(17)-C(16)	108.8(4)
C(18)-C(17)-H(17A)	109.9
C(16)-C(17)-H(17A)	109.9
C(18)-C(17)-H(17B)	109.9
C(16)-C(17)-H(17B)	109.9
H(17A)-C(17)-H(17B)	108.3
C(23)-C(18)-C(19)	120.5(5)
C(23)-C(18)-C(17)	120.5(5)
C(19)-C(18)-C(17)	119.0(5)
C(22)-C(23)-C(18)	121.6(8)
C(22)-C(23)-H(23)	119.2
C(18)-C(23)-H(23)	119.2
C(21)-C(22)-C(23)	120.2(8)
C(21)-C(22)-H(22)	119.9
C(23)-C(22)-H(22)	119.9
C(22)-C(21)-C(20)	120.4(7)
C(22)-C(21)-H(21)	119.8
C(20)-C(21)-H(21)	119.8
C(21)-C(20)-C(19)	119.5(7)
C(21)-C(20)-H(20)	120.3
C(19)-C(20)-H(20)	120.3
C(18)-C(19)-C(20)	117.8(6)
C(18)-C(19)-H(19)	121.1
C(20)-C(19)-H(19)	121.1
C(9)-N(1)-H(1A)	109.5

C(9)-N(1)-H(1B)	109.5
H(1A)-N(1)-H(1B)	109.5
C(9)-N(1)-H(1C)	109.5
H(1A)-N(1)-H(1C)	109.5
H(1B)-N(1)-H(1C)	109.5
C(3)-N(2)-C(16)	111.7(3)
C(3)-N(2)-C(1)	113.5(3)
C(16)-N(2)-C(1)	112.0(3)
C(3)-N(2)-H(2)	106.4
C(16)-N(2)-H(2)	106.4
C(1)-N(2)-H(2)	106.4
C(12)-O(1)-H(1D)	109.5
H(2A)-O(2)-H(2B)	104.5
C(1)-C(8)-C(7)	115.2(3)
C(1)-C(8)-H(8A)	108.5
C(7)-C(8)-H(8A)	108.5
C(1)-C(8)-H(8B)	108.5
C(7)-C(8)-H(8B)	108.5
H(8A)-C(8)-H(8B)	107.5

Table S4. Anisotropic displacement parameters ($Å^2$x 10^3) for **23**·2HCl·MeOH·H_2O. The anisotropic displacement factor exponent takes the form: $-2\pi^2[h^2a^{*2}U^{11} + ... + 2 h k a^* b^* U^{12}]$

	U^{11}	U^{22}	U^{33}	U^{23}	U^{13}	U^{12}
C(12)	28(2)	46(2)	21(2)	-12(2)	-6(1)	3(2)
C(13)	42(2)	40(2)	27(2)	-10(2)	-4(2)	8(2)
C(14)	53(3)	31(2)	30(2)	-2(2)	-5(2)	-1(2)
C(15)	36(2)	38(2)	21(2)	0(2)	-4(2)	-4(2)
C(10)	24(2)	34(2)	17(1)	-2(1)	-4(1)	1(1)
C(11)	23(2)	35(2)	22(2)	-7(1)	-5(1)	0(1)
C(5)	19(2)	35(2)	18(2)	2(1)	-2(1)	-1(1)
C(6)	18(2)	41(2)	23(2)	3(1)	-3(1)	-6(2)
C(7)	16(2)	44(2)	34(2)	10(2)	0(1)	-1(2)
C(1)	19(2)	29(2)	20(1)	6(1)	2(1)	5(1)
C(9)	14(1)	28(2)	17(1)	3(1)	0(1)	1(1)
C(4)	22(2)	43(2)	19(2)	6(2)	-3(1)	2(2)
C(3)	22(2)	40(2)	23(2)	9(1)	-3(1)	4(1)
C(16)	26(2)	32(2)	33(2)	8(2)	3(2)	8(2)
C(17)	48(3)	41(2)	48(2)	20(2)	13(2)	14(2)
C(18)	39(2)	34(2)	37(2)	15(2)	5(2)	5(2)
C(23)	74(4)	49(3)	78(4)	20(3)	-7(3)	-23(3)
C(22)	129(8)	50(4)	89(5)	4(3)	4(5)	-28(5)
C(21)	161(9)	33(3)	82(5)	2(3)	48(6)	-4(4)
C(20)	77(5)	77(4)	110(6)	46(4)	35(4)	48(4)
C(19)	48(3)	51(3)	67(3)	20(2)	1(3)	16(2)
Cl(1)	29(1)	28(1)	24(1)	1(1)	-5(1)	1(1)
Cl(2)	19(1)	53(1)	53(1)	32(1)	6(1)	3(1)
N(1)	17(1)	26(1)	17(1)	1(1)	-2(1)	1(1)
N(2)	20(1)	31(2)	22(1)	8(1)	2(1)	3(1)
O(1)	25(1)	51(2)	38(2)	-22(1)	3(1)	-1(1)
O(2)	31(2)	37(2)	61(2)	5(1)	-3(2)	3(1)
C(8)	21(2)	33(2)	29(2)	8(2)	9(1)	6(1)
O(3A)	81(5)	90(5)	60(4)	13(3)	-22(4)	-28(4)
C(24A)	80(5)	85(5)	60(4)	13(4)	-22(4)	-29(4)
O(3B)	93(7)	62(5)	66(6)	8(4)	-10(5)	5(6)
C(24B)	94(7)	62(5)	68(6)	8(4)	-9(5)	5(6)

Table S5. Hydrogen coordinates (x 10^4) and isotropic displacement parameters ($Å^2$x 10^3) for **23**·2HCl·MeOH·H_2O.

	x	y	z	U(eq)
H(13)	458	10364	7260	43
H(14)	3280	10755	6852	46
H(15)	5305	9571	6566	38
H(11)	1553	7542	7018	32
H(6A)	6607	8475	6103	33
H(6B)	7559	8310	6633	33
H(7A)	8786	6855	6401	38
H(7B)	9163	7527	5934	38
H(1)	4499	5856	5688	27
H(9)	2735	6781	6283	24
H(4A)	6144	7168	7255	33
H(4B)	4078	6769	7181	33
H(3A)	7465	5927	6821	34
H(3B)	5895	5427	7155	34
H(16A)	7408	4584	6215	36
H(16B)	5506	4300	5940	36
H(17A)	6378	3871	6964	55
H(17B)	4336	3691	6736	55
H(23)	3909	2406	6100	80
H(22)	4880	975	5760	107
H(21)	7901	433	5889	111
H(20)	10045	1355	6358	106
H(19)	8994	2813	6744	66
H(1A)	2949	7300	5458	24
H(1B)	4569	7916	5579	24
H(1C)	2703	8132	5791	24
H(2)	3856	5338	6495	29
H(1D)	-1196	9059	7635	57
H(2A)	996	9068	5854	64
H(2B)	1359	9708	5481	64
H(8A)	6994	6769	5453	33
H(8B)	7916	5907	5751	33

Table S6. Torsion angles [°] for **23**·2HCl·MeOH·H_2O.

O(1)-C(12)-C(13)-C(14)	177.7(3)
C(11)-C(12)-C(13)-C(14)	-1.9(6)
C(12)-C(13)-C(14)-C(15)	-0.9(6)
C(13)-C(14)-C(15)-C(10)	2.1(6)
C(14)-C(15)-C(10)-C(11)	-0.4(5)
C(14)-C(15)-C(10)-C(5)	179.9(3)
O(1)-C(12)-C(11)-C(10)	-176.0(3)
C(13)-C(12)-C(11)-C(10)	3.6(5)
C(15)-C(10)-C(11)-C(12)	-2.4(5)
C(5)-C(10)-C(11)-C(12)	177.3(3)
C(15)-C(10)-C(5)-C(9)	-117.2(4)
C(11)-C(10)-C(5)-C(9)	63.1(4)
C(15)-C(10)-C(5)-C(6)	3.8(4)
C(11)-C(10)-C(5)-C(6)	-175.8(3)
C(15)-C(10)-C(5)-C(4)	127.2(4)
C(11)-C(10)-C(5)-C(4)	-52.4(4)
C(10)-C(5)-C(6)-C(7)	-176.1(3)
C(9)-C(5)-C(6)-C(7)	-54.9(4)
C(4)-C(5)-C(6)-C(7)	62.7(4)
C(5)-C(6)-C(7)-C(8)	44.1(4)
N(2)-C(1)-C(9)-N(1)	-168.0(3)
C(8)-C(1)-C(9)-N(1)	66.3(3)
N(2)-C(1)-C(9)-C(5)	68.5(3)
C(8)-C(1)-C(9)-C(5)	-57.2(4)
C(10)-C(5)-C(9)-N(1)	62.2(3)
C(6)-C(5)-C(9)-N(1)	-60.7(3)
C(4)-C(5)-C(9)-N(1)	178.6(3)
C(10)-C(5)-C(9)-C(1)	-176.4(3)
C(6)-C(5)-C(9)-C(1)	60.8(3)
C(4)-C(5)-C(9)-C(1)	-60.0(3)
C(10)-C(5)-C(4)-C(3)	164.7(3)
C(9)-C(5)-C(4)-C(3)	47.3(4)
C(6)-C(5)-C(4)-C(3)	-71.7(4)
C(5)-C(4)-C(3)-N(2)	-42.7(4)
N(2)-C(16)-C(17)-C(18)	172.2(4)
C(16)-C(17)-C(18)-C(23)	-86.7(5)
C(16)-C(17)-C(18)-C(19)	91.8(5)

C(19)-C(18)-C(23)-C(22)	-0.5(9)
C(17)-C(18)-C(23)-C(22)	178.0(6)
C(18)-C(23)-C(22)-C(21)	-0.4(11)
C(23)-C(22)-C(21)-C(20)	-0.4(12)
C(22)-C(21)-C(20)-C(19)	2.0(11)
C(23)-C(18)-C(19)-C(20)	2.0(8)
C(17)-C(18)-C(19)-C(20)	-176.5(5)
C(21)-C(20)-C(19)-C(18)	-2.7(9)
C(4)-C(3)-N(2)-C(16)	177.7(3)
C(4)-C(3)-N(2)-C(1)	49.9(4)
C(17)-C(16)-N(2)-C(3)	64.4(4)
C(17)-C(16)-N(2)-C(1)	-167.1(4)
C(8)-C(1)-N(2)-C(3)	62.1(4)
C(9)-C(1)-N(2)-C(3)	-62.5(3)
C(8)-C(1)-N(2)-C(16)	-65.5(4)
C(9)-C(1)-N(2)-C(16)	169.9(3)
N(2)-C(1)-C(8)-C(7)	-74.9(4)
C(9)-C(1)-C(8)-C(7)	46.1(4)
C(6)-C(7)-C(8)-C(1)	-39.1(4)
